# Dabrafenib and trametinib administration in patients with *BRAF* V600E/R or non-V600 *BRAF* mutated advanced solid tumours (BELIEVE, NCCH1901): a multicentre, open-label, and single-arm phase II trial

**DOI:** 10.1016/j.eclinm.2024.102447

**Published:** 2024-02-02

**Authors:** Tatsunori Shimoi, Kuniko Sunami, Makoto Tahara, Satoshi Nishiwaki, Shota Tanaka, Eishi Baba, Masashi Kanai, Ichiro Kinoshita, Hidekazu Shirota, Hideyuki Hayashi, Naohiro Nishida, Toshio Kubo, Nobuaki Mamesaya, Yayoi Ando, Natsuko Okita, Taro Shibata, Kenichi Nakamura, Noboru Yamamoto

**Affiliations:** a Department of Medical Oncology, National Cancer Center Hospital, Tokyo, Japan; b Department of International Clinical Development, National Cancer Center Hospital, Tokyo, Japan; c Department of Laboratory Medicine, National Cancer Center Hospital, Tokyo, Japan; d Department of Head and Neck Medical Oncology, National Cancer Center Hospital East, Kashiwa, Japan; e Department of Advanced Medicine, Nagoya University Hospital, Aichi, Japan; f Department of Neurosurgery, Graduate School of Medicine, The University of Tokyo, Tokyo, Japan; g Department of Oncology and Social Medicine, Graduate School of Medical Sciences, Kyushu University, Fukuoka, Japan; h Department of Therapeutic Oncology, Graduate School of Medicine, Kyoto University, Kyoto, Japan; i Department of Medical Oncology, Hokkaido University Hospital, Hokkaido, Japan; j Department of Medical Oncology, Tohoku University Hospital, Sendai, Miyagi, Japan; k Genomics Unit, Keio Cancer Center, Keio University School of Medicine, Tokyo, Japan; l Center for Cancer Genomics and Personalized Medicine, Osaka University Hospital, Osaka, Japan; m Center for Clinical Oncology, Okayama University Hospital, Japan; n Division of Thoracic Oncology, Shizuoka Cancer Center, Shizuoka, Japan; o Research Management Division, Clinical Research Support Office, National Cancer Center Hospital, Tokyo, Japan; p Biostatistics Division, Center for Research Administration and Support, National Cancer Center, Tokyo, Japan; q Department of Experimental Therapeutics, National Cancer Center Hospital, Tokyo, Japan

**Keywords:** Bayesian statistics, Platform clinical trials, BRAF inhibitors, MEK inhibitors, Rare cancers

## Abstract

**Background:**

*BRAF* V600 mutations are common in melanoma, thyroid, and non-small-cell lung cancers. Despite dabrafenib and trametinib being standard treatments for certain cancers, their efficacy across various solid tumours remains unelucidated. The BELIEVE trial assessed the efficacy of dabrafenib and trametinib in solid tumours with *BRAF* V600E/R or non-V600 *BRAF* mutations.

**Methods:**

Between October 1, 2019, and June 2022, at least 50 patients with measurable and seven without measurable diseases examined were enrolled in a subcohort of the BELIEVE trial (NCCH1901, jRCTs031190104). *BRAF* mutated solid tumour cases other than *BRAF* V600E mutated colorectal cancer, melanoma, and non-small cell lung cancer cases were included. Patients with solid tumours received dabrafenib (150 mg) twice daily and trametinib (2 mg) once daily until disease progression or intolerable toxicity was observed. The primary endpoint was overall response rate (ORR), and secondary endpoints included progression-free survival (PFS), 6-month PFS, and overall survival (OS). Bayesian analysis was performed using a prior distribution with a 30% expected response rate [Beta (0.6, 1.4)].

**Findings:**

Fourty-seven patients with measurable disease, mainly with the *BRAF* V600E mutation (94%), and three others with non-V600E *BRAF* mutations (V600R, G466A, and N486_P490del) were enrolled. The primary sites included the thyroid gland, central nervous system, liver, bile ducts, colorectum, and pancreas. The confirmed ORR was 28.0%; the expected value of posterior distribution [Beta (14.6, 37.4)] was 28.1%, although the primary endpoint was achieved, not exceeding an unexpectedly high response rate of 60% obtained using Bayesian analysis. The disease control rate (DCR) was 84.0%. The median PFS was 6.5 months (95% confidence interval [CI]; 4.2–7.2 months, 87.8% at 6 months). Responses were observed across seven tumour types. Median OS was 9.7 months (95% CI, 7.5–12.2 months). Additional patients without measurable diseases had a median PFS of 4.5 months. Adverse events (AEs) were consistent with previous reports, with 45.6% of patients experiencing grade ≥3 AEs.

**Interpretation:**

This study reported promising efficacy against *BRAF* V600-mutant tumours. Dabrafenib and trametinib would offer a new therapeutic option for rare cancers, such as high-grade gliomas, biliary tract cancer, and thyroid cancer.

**Funding:**

This study was funded by the 10.13039/100009619Japan Agency for Medical Research and Development (22ck0106622h0003) and a Health and Labour Sciences Research Grant (19EA1008).

**Research in context**

**Evidence before this study**

We conducted a comprehensive literature search on PubMed using the terms “BRAF,” “dabrafenib,” “trametinib,” “clinical trial,” and “solid tumour,” resulting in 48 publications. Combination therapy with dabrafenib and trametinib consisted of dabrafenib (150 mg) twice daily and trametinib (2 mg) once daily orally. Among these, only three clinical trials included patients with rare cancers, namely the ROAR trial, NCI-MATCH subprotocol and thyroid cancer cases other than non-small cell lung cancer or melanoma. On June 22, 2022, the Food and Drug Administration (FDA) granted accelerated approval for dabrafenib (Tafinlar, Novartis) in combination with trametinib (Mekinist, Novartis) to treat adult and paediatric patients with unresectable or metastatic solid tumours harbouring BRAF V600E mutation, who have experienced disease progression after prior treatment and have limited alternative therapeutic options.

The FDA's decision on the combination of dabrafenib and trametinib for the treatment of solid tumours is based on data from several studies, including the ROAR and NCI-MATCH trials, involving over 240 adult and paediatric patients. The findings on efficacy and safety from these studies have been reinforced by results from the COMBI-d, COMBI-v, and BRF113928 trials. However, specific data on nine differentiated thyroid cancer cases were derived from the ROAR and Phase 2 trials, leading to heightened anticipation for additional real-world efficacy and safety data.

**Added value of this study**

In this report, we present data from carcinomas not encompassed in the aforementioned trials, including malignant soft-tissue tumours, cancers of unknown primary, and breast cancer.

**Implications of all the available evidence**

We believe that the accumulation of efficacy data across diverse histologic types, as observed in the FDA studies, will provide valuable insights for the application of this therapeutic approach in clinical practice.

## Introduction

Activating mutations in the *BRAF* gene have received widespread attention in the past decade; nearly half of all melanomas have been shown to harbour mutations in *BRAF* at codon 600, which results in the constitutive activation of the MAPK pathway.^1^^,^^2^ Additionally, approximately 3% of all non-small cell lung cancer cases exhibit these mutations, and poor prognosis has been reported in these tumours.^3^ V600 mutations in *BRAF*, which activate the MAPK pathway, have been identified in 5–15% of cases of low-grade gliomas but are less frequent in high-grade gliomas, such as glioblastomas (approximately 3%).^4^ Moreover, in The Cancer Genome Atlas (TCGA) cohort for papillary thyroid cancer, 59% of 496 samples had activating *BRAF* mutations, most of which were V600E substitutions.^5^ Alterations in *BRAF* occur at a lower frequency in most other tumour types, with the incidence of its mutations estimated to be approximately 1–3% in all cancers.^6^ However, the successful inhibition of this pathway using BRAF/MEK inhibitors has yielded clinically relevant benefits, leading to the establishment of these agents as standard treatment options for malignant melanoma and non-small cell lung cancer.

The National Cancer Institute Molecular Analysis for Therapy Choice (NCI-MATCH) trial, developed by the ECOG-ACRIN Cancer Research Group and the National Cancer Institute (NCI), some cohorts explored the efficacy of administering dabrafenib and trametinib in patients with solid tumours in the United States. The Phase 2 trial of administering them (the NCI-MATCH trial subprotocol H) in patients with certain *BRAF*-mutated cancers, excluding melanoma and non-small cell lung, thyroid, and colorectal cancers, reported a median progression-free survival (PFS) of 11.4 months and a response rate of 38%.^7^ Conversely, in another NCI-MATCH trial, trametinib alone in solid tumours and lymphomas with BRAF non-V600 mutations did not show promising results, with a response rate of 3%.^8^ In non-V600 BRAF mutant melanoma, trametinib ± dabrafenib exhibited some efficacy.^9^^,^^10^

The Rare Oncology Agnostic Research (ROAR) basket trial evaluated the activity and safety of dabrafenib and trametinib combination therapy in patients with *BRAF* V600E-positive anaplastic thyroid cancer, biliary tract cancer, gastrointestinal stromal tumour, small bowel adenocarcinoma, non-seminomatous or non-germinomatous germ cell tumour, and mutated rare cancers, including hairy cell leukaemia, low-grade gliomas, high-grade gliomas, and multiple myeloma. The results showed that the response rates for high- and low-grade gliomas in biliary tract cancer were 33% and 51%, respectively.^11^^,^^12^ Based on these data, the FDA approved the combination therapy of dabrafenib and trametinib in August 2022 for solid tumours with *BRAF* V600E mutations.

The advent of precision medicine is largely due to the advancements in genome analysis technology, particularly next-generation sequencing (NGS), which enables comprehensive genome profiling (CGP) to simultaneously assess multiple genomic aberrations in cancer tissue samples. However, drug availability varies by country; previous reports from the study from Memorial Sloan Kettering Cancer Center with CGP test (MSK-IMPACT®) in the United States indicated that 8% of patients receive off-label drug recommendations based on CGP testing.^13^

Japan provides a universal health insurance system. However, off-label drug use is not reimbursed under this scheme. Exceptions are made for the drugs used in the unique Japanese clinical research system, such as registration-directed trials, advanced medical care, and patient-proposed healthcare services (PPHS).^14^^,^^15^ Despite the widespread availability of CGP testing for cancer to the general public in Japan since 2019, clinical trials providing patients with treatment options based on the results are scarce. In response to these social demands, similar to the NCI-MATCH trial, the National Cancer Center Hospital in Japan has launched a prospective study of PPHS with multiple targeted agents based on the results of gene profiling by multigene panel testing. This study, known as BELIEVE (NCCH1901, jRCTs031190104), represents the Japanese version of the NCI-MATCH and aims to identify signals of efficacy for therapies targeting molecular changes found in all tumour types.^16^^,^^17^ As of March 2023, there were 20 drug cohorts. In this study, we presented an analysis of a patient cohort treated with a combination of dabrafenib and trametinib.

## Methods

### Patient selection

The BELIEVE trial (The Prospective Trial of Patient-Proposed Healthcare Services with Multiple Targeted Agents Based on the Result of Gene Profiling by Multigene Panel Test; NCCH1901, jRCTs031190104) aims to identify indications of efficacy for therapies that target actionable molecular changes found in all tumour types. A comprehensive overview of the trial has been previously published.^17^, ^18^, ^19^

We conducted a multicentre, open-label, and single-arm phase II basket study of patients with solid tumours who were prescribed dabrafenib and trametinib based on the CGP results. However, melanomas and non-small cell lung cancer harbouring the *BRAF* V600E mutation were excluded from the study because of being a well-studied cancer type and upon the request from a pharmaceutical company, respectively. Cases of colorectal cancers harbouring the *BRAF* V600E mutation were excluded in a later amendment. The study included patients aged ≥15 years with an Eastern Cooperative Oncology Group performance status of 0 or 1 and normal organ function, as assessed during routine biochemistry and haematology tests. Patients were recruited from 12 designated core hospitals for cancer genomic medicine, namely community and academic cancer centres.

Patients with solid tumours who had completed standard therapy, which is considered the first choice of treatment in all guidelines according to the treatment methods, were considered eligible. Patients with and without measurable disease states were eligible for enrolment in the study. All cases were reviewed by the molecular tumour board at each participating institution based on the clinical practice guidelines for next-generation sequencing in cancer diagnosis and treatment (edition 2.1). Further, all cases have been confirmed (level of evidence A to D) to be at the level of evidence reported for this drug in humans.^20^

### Ethics

The study protocol (Supplementary protocol file) was approved by the Central Research Ethics Review Committee (CTA-C19013) and the Patient Application Evaluation Committee of the Ministry of Health, Labour and Welfare (MHLW) and was conducted following the ethical principles of the Declaration of Helsinki and all applicable local regulations. All patients provided written informed consent prior to enrolment.

### Procedures

Patients received oral dabrafenib (150 mg) twice and trametinib (2 mg) once daily until the disease progression or toxicity exceeded tolerable levels. Treatment interruption was permitted in patients unable to tolerate the specified doses until tolerability improved. Dosage adjustment was allowed based on the instructions on the package inserts of the prescription drugs.

The patients underwent whole-body contrast-enhanced computed tomography (CT) and/or contrast-enhanced magnetic resonance imaging (MRI). CT was performed routinely at 16 weeks and subsequently at the physician's discretion until disease progression. Although the researchers recommended CT imaging in the 8th week, it was not mandatory. Laboratory assessments during screening included clinical chemistry and haematological evaluations, which were performed monthly during treatment. Safety assessments were performed by monitoring adverse events (AEs) using the Common Terminology Criteria for Adverse Events (version 5.0), beginning from the administration of the first dose until 30 days after treatment discontinuation. The protocol stipulates that safety data should be summarised across all histological types and cohorts.

### Outcomes

The primary endpoint in each drug cohort was the confirmed overall response rate (ORR) in a case population with measurable disease, defined as a complete or partial response (CR or PR, respectively) using RECIST version 1.1, as assessed by the investigator up to 16 ± 1 weeks after the initiation of treatment. The secondary endpoints were PFS, duration of response, disease control rate (confirmed CR + confirmed PR + stable disease), overall survival (OS) in a case population with measurable disease, and safety in all cases. PFS was defined as the duration starting from the first dose of study treatment to documented disease progression or death from any cause, whichever occurred first. OS was defined as the duration starting from the first dose of the study treatment to death from any cause.

### Statistical considerations

The efficacy of each drug cohort is expected to be evaluated in a maximum of 50 patients with measurable lesions; up to 30 patients with non-measurable lesions may be enrolled.

As a primary analysis, Bayesian inference was drawn using a prior distribution with an expected response rate of 30% [Beta (0.6, 1.4)]. Although the response rate of the drug was expected at 30% and the clinical hypothesis was whether the enrolled patients achieved response to the drug, early termination was considered if the posterior distribution yielded significantly higher or lower value than the prior expectation, that is 60% and 20%, respectively, with high probability (>95%).^21^ An exact method based on binomial distribution (Clopper-Pearson method) was also used to estimate the response rate interval for frequentist theory and calculate 95% confidence intervals (two-sided).

The endpoints, progression-free survival, and OS, were also analysed by using the Kaplan–Meier method.

All statistical analyses were performed using SAS version 9.4. The trial was registered in the Japan Registry of Clinical Trials database (jRCTs031190104).

### Role of funding source

This study received funding from the Japan Agency for Medical Research and Development (JP20ck0106622) and a Health and Labour Sciences Research Grant (19EA1008). The funding source has been used for the operation of platform-based trials (e.g., EDC construction for clinical trials, administrative costs, transportation of drugs, etc.). Funders are not involved in study design, data collection, data analysis, interpretation, or reporting.

## Results

**Fig. 1:**
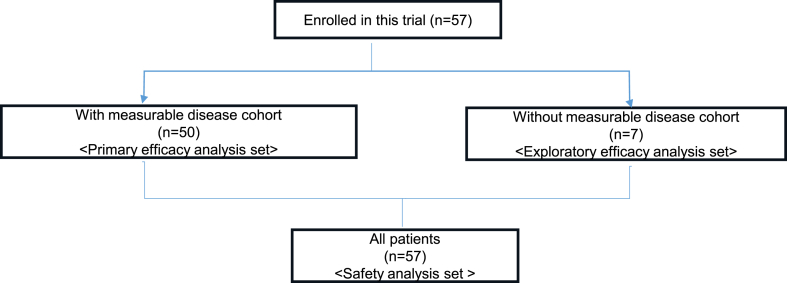
**CONSORT flowchart of participants**.

Fifty patients with measurable diseases and seven without measurable diseases examined between October 1, 2019, and June 2022, were enrolled. Fig. 1 summarises the CONSORT Flowchart of participant enrollment in this study. The characteristics of all 57 patients included in the primary analysis are summarised in Table 1. A total of 56% of the patients were females, with a median age of 56.5 years (16–77 years). All patients were of Asian descent. The most common primary sites in the main cohort were the thyroid gland (n = 15, 30%; including 10 differentiated and five undifferentiated/anaplastic histologies), central nervous system (CNS; n = 9; high-grade glioma, 19%), and biliary tract (n = 6, 12%; including four intrahepatic bile ducts, one extrahepatic biliary tract, and one ampulla of Vater).

**Table 1:** Baseline characteristics of the intention-to-treat population.

	Cohort with measurable disease (n = 50)	Cohort without measurable disease (n = 7)
Age, years, median	56·5 (16–77)	33 (25–42)
Female	28 (56%)	4 (57%)
Asian ethnicity	50 (100%)	7 (100%)
Performance status		
0	28 (56%)	2 (29%)
1	22 (44%)	5 (71%)
Primary sites		
Endocrine organ (thyroid cancer)	15 (30%)	0
Glial tumour of CNS	12 (24%)	4 (57%)
Liver and intrahepatic duct	4 (8%)	0
Colorectal	3 (6%)	1 (14%)
Non glial tumour of CNS and pineal gland	3 (6%)	0
Pancreas	3 (6%)	0
Gallbladder and extrahepatic duct	2 (4%)	0
Others	8 (16%)	2 (29%)
Previous antineoplastic regimens		
0	11 (22%)	3 (43%)
1	12 (24%)	2 (29%)
2	12 (24%)	1 (14%)
3	1 (2%)	0 (0%)
>3	4 (8%)	1 (14%)
ND	10 (20%)	0 (0%)
Prior surgery	43 (86%)	7 (100%)
Prior radiotherapy	26 (52%)	4 (57%)
Mutation status		
*BRAF* V600E	47 (94%)	7 (100%)
*BRAF* V600R	1 (2%)	0
*BRAF* G466A	1 (2%)	0
*BRAF* N486_P490del	1 (2%)	0

ND; not determined.

Forty-seven patients with the *BRAF* V600E mutation, one with the *BRAF* V600R mutation (another Class 1 *BRAF* mutation), and one each with G466A and N486_P490del mutations (Class 2 *BRAF* mutations) were included in the study. The patient with a solid tumour who had the N486_P490del mutation was the only patient with melanoma.

### Main cohort efficacy results

Fifty patients with measurable diseases from the main cohort were included in the primary efficacy analysis; two were not evaluated with appropriate imaging at 16 weeks.

**Fig. 2:**
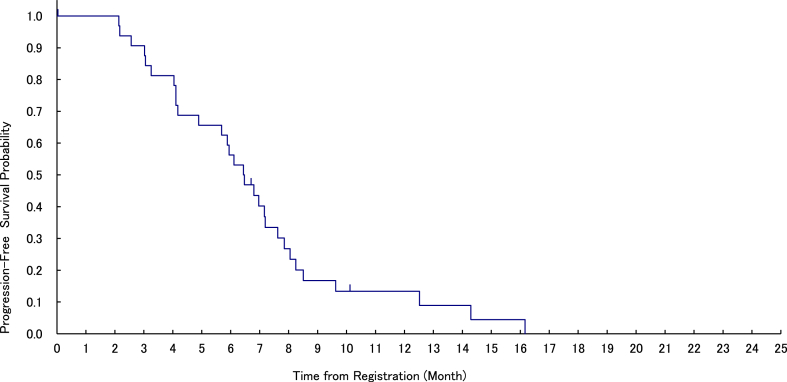
**Progression-free survival by investigator assessment (in the main cohort with measurable disease, n = 50)**. The median progression-free survival time is 6.5 months (95% confidence interval, 4.2–7.2 months). The progression-free rate at 6 months is 56.3% (95% confidence interval, 37.6–71.3%).

**Fig. 3:**
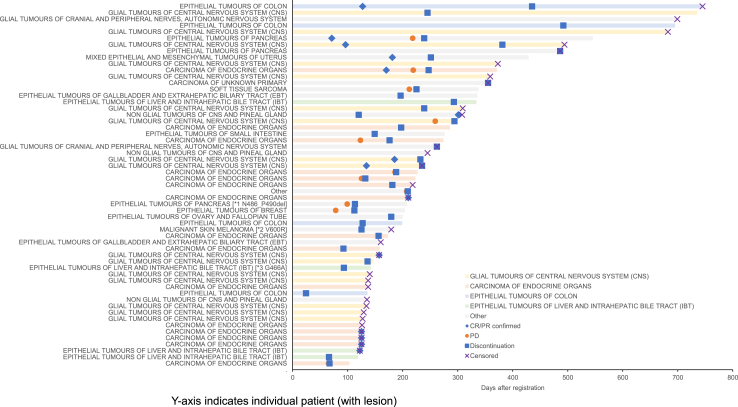
**Duration of treatment in patients who achieved partial response or stable disease**. Duration of treatment and overall survival in patients who achieved partial response (PR) or stable disease. CR/PR, complete response/partial response; PD, progressive disease.

The confirmed ORR was 28.0% (95% CI, 16.2–42.5%), and the median duration of response was 5.2 months (Range, 1.2–22.2 months). A total of 28 patients displayed stable disease (SD), with a disease control rate (DCR) of 84.0% (Table 2). Median follow-up duration was 7.0 months, median PFS was 6.5 months (95% CI, 4.2–7.2 months; Fig. 2), and the 6-month PFS rate was 56.3% (95% CI, 37.6–71.3%). Median OS was 9.7 months (95% CI, 7.5–12.2 months), despite the events not being mature (Supplementary Figure S1). At the data cut-off in April 2022, 13 patients (26.0%) were still on treatment. Most of the genomic abnormalities were of the BRAFV600E mutations, but other *BRAF* mutations included not evaluable (NE) in cases with *G466A* and N486_P490del and progressive disease (PD) in cases with *V600R*. Durable partial response (PR) were observed in various tumour types (Fig. 3). The rates by tissue were 33.3% (5/15 patients) for thyroid cancer, 33.3% (4/12 patients) for CNS tumours, and 12.3% (1/6 patients) for biliary tract cancer (Table 3).

**Table 2:** Best overall response^a^ by 16 weeks in the intention-to-treat and evaluable populations.

	Cohort with measurable disease (n = 50)	Cohort without measurable disease (n = 7)
Best response		
Complete response	0	0
Partial response	14 (28%)	0
Stable disease	28 (56%)	–
Non-CR/non-PD	–	5 (72%)
Progressive disease	6 (12%)	1 (14%)
Not evaluable	2 (4%)	1 (14%)
Overall response rate (complete response plus partial response)	14 (28%)	0
Disease control rate (complete response plus partial response plus stable disease or non-CR/non-PD)	42 (84%)	5 (72%)

a Investigator Accessed. CR, complete response; PD, progressive disease.

**Table 3:** Best overall response and progression-free survival by histological type.

	CR	PR	SD	PD	NE	Total	ORR	PFS median (95% CI)
Carcinoma of endocrine organs (thyroid cancer)	0	5	6	4	0	15	33.3%	5.7 (2.1–6.5)
Glial tumours of the central nervous system (CNS)^a^	0	4	8	0	0	12	33.3%	8.0 (7.6–12.5)
Epithelial tumours of the liver and intrahepatic bile tract (IBT)	0	1	0	1	2	4	25.0%	3.1 (2.2–9.6)
Epithelial tumours of the pancreas	0	1	2	0	0	3	33.3%	5.2 (3.3–7.2)
Epithelial tumours of the colon	0	1	2	0	0	3	33.3%	14.3 (4.2–16.2)
Non-glial tumours of the CNS and pineal gland	0	1	2	0	0	3	33.3%	NA (NA)
Epithelial tumours of the gallbladder and extrahepatic biliary tract (EBT)	0	0	2	0	0	2	0%	6.4 (NA)
Mixed epithelial and mesenchymal tumours of the uterus	0	1	0	0	0	1	100%	8.2 (NA)
Carcinoma of unknown primary site	0	0	1	0	0	1	0%	NA (NA)
Epithelial tumours of the ovary and fallopian tube	0	0	1	0	0	1	0%	5.9 (NA)
Epithelial tumours of the small intestine	0	0	1	0	0	1	0%	4.9 (NA)
Malignant skin melanoma	0	0	1	0	0	1	0%	4.1 (NA)
Soft tissue sarcoma	0	0	1	0	0	1	0%	7.0 (NA)
Epithelial tumours of the breast	0	0	0	1	0	1	0%	2.6 (NA)
Other	0	0	1	0	0	1	0%	6.8 (NA)
Total	0	14	28	6	2	50	28.0%	6.5 (4.2–7.2)

PFS, progression free survival; CR, complete response; PR, partial response; SD, stable disease; PD, progressive disease; NE, not evaluable; NA, not assessable.

a Glioblastoma and high-grade glioma.

### Exploratory cohort efficacy results

Seven patients without measurable diseases were classified into the exploratory analysis cohort for exploratory analysis of efficacy; one was not evaluated with appropriate imaging at 16 weeks. None of the patients responded to the treatment. Five patients had non-CR/non-PD, with a disease control rate (DCR) of 71.4% (Supplementary Table S2). Median follow-up was 11.8 months, median PFS was 4.5 months (95% CI, 0.8–8.6 months; Supplementary Figure S2), and the 6-month PFS rate was 33.3% (95% CI, 0.9–77.4%). Median OS was not estimated Supplementary Figure S2. The genomic abnormality in all patients was *BRAF V600E*.

### Safety

AE analysis included all the treated patients (n = 57). The AEs were comparable to those previously reported for dabrafenib and trametinib; new AEs were not identified. A total of 45.6% of the patients had grade ≥3 AEs.

The most frequent AEs were pyrexia (26.3%), anaemia (12.3%), anorexia (8.8%), elevated aspartate aminotransferase levels (8.8%), and hypoalbuminemia (8.8%). Dose reductions were observed 30 times in 10 patients. AE discontinuations were observed in three patients (Supplementary Tables S1 and S2).

In the ROAR study, pyrexia was found in 24%, decreased appetite in six (13%), and increased aspartate aminotransferase in 18% of the high-grade glioma cases.^11^

The main grade 3 AEs suspected to be related to treatment were decreased neutrophil count (7.0%) and pyrexia (5.3%). Grade 4 AEs, namely creatine phosphokinase increase, were observed in one case (Table 4). Grade 5 AEs were not observed.

**Table 4:** Adverse events (n = 57).

	Any causality			Related to treatment		
	No. of patients (%)			No. of patients (%)		
Adverse events reported more than 3 patients	Any Grade	Grade 3	Grade 4	Any Grade	Grade 3	Grade 4
Pyrexia	15 (26.3)	3 (5.3)	0	14 (24.6)	3 (5.3)	0
Anaemia	7 (12.3)	3 (5.3)	0	2 (3.5)	1 (1.8)	0
Neutropenia	6 (10.5)	2 (3.5)	1 (1.8)	5 (8.8)	4 (7)	0
Anorexia	5 (8.8)	1 (1.8)	0	1 (1.8)	0	0
Elevated aspartate aminotransferase	5 (8.8)	2 (3.5)	0	2 (3.5)	1 (1.8)	0
Elevated alanine aminotransferase	4 (7)	3 (5.3)	0	1 (1.8)	1 (1.8)	0
Elevated alkaline phosphatase	4 (7)	1 (1.8)	0	1 (1.8)	1 (1.8)	0
Elevated creatine phosphokinase	4 (7)	2 (3.5)	1 (1.8)	3 (5.3)	2 (3.5)	1 (1.8)
Hypoalbuminemia	4 (7)	0	0	0	0	0
Disease progression	4 (7)	0	4 (7)	0	0	0
Acne rash	4 (7)	0	0	4 (7)	0	0
Rash	4 (7)	0	0	4 (7)	0	0
Fatigue	3 (5.3)	0	0	1 (1.8)	0	0
Paronychia	3 (5.3)	0	0	1 (1.8)	0	0
Elevated γ-glutamyl trans peptidase	3 (5.3)	2 (3.5)	0	0	0	0
Leukopenia	3 (5.3)	1 (1.8)	0	2 (3.5)	0	0

Data are reported as n (%); events are reported for two or more patients.

## Discussion

The study met its primary endpoint with an ORR of 28%, which is similar to the prior setting in Bayesian statistics. In April 2022, the FDA approved the combination therapy of dabrafenib and trametinib for solid tumours with the *BRAF* V600E mutation. This trial was conducted before approval, and its results suggest that these therapies are promising.

The response rate reported in this study was lower than that for the standard treatment of diseases, such as melanoma and non-small cell lung cancer (NSCLC); however, an overall DCR of 84.0% was achieved. Importantly, disease control was sustained in several patients, indicated by a median response of 5.2 months, median PFS of 6.5 months, and PFS at 6 months of 54.3%. The median follow-up period in this study was very short (7 months), and no conclusions could be drawn about survival rates.

The results were particularly promising in the patient population, wherein the study was conducted on patients who had received other standard therapies and in whom treatment options were limited. The response rate to treatment of thyroid cancer was 33%. In prior phase II trials, a response rate of 30% was documented for the combination of dabrafenib and trametinib in differentiated thyroid cancer.^22^ This trial included anaplastic histology and reported a notable response rate of 33%.

The study also showed a 33.3% response in high-grade gliomas of the CNS for which there are few standard treatments, similar to the results of the recently published ROAR study (33%).^11^^,^^23^

For these tumour types where the prevalence of *BRAF* mutations is low, a phase 3 trial would be impossible to conduct, and thus, dabrafenib/trametinib should be a viable treatment option for this small subpopulation of patients.

In *BRAF* V600 mutant melanoma cell lines and tumours, preexisting genomic alterations and factors endogenously secreted by stromal and tumour cells explain primary resistance to BRAF inhibition. These mostly include PTEN loss, Cyclin D1 amplification, RAC1 mutations, HOXD8 mutations, MEK mutations, NF1 loss, and activation of the c-Jun/RHOB axis among others.^2^^,^^24^ In the BELIEVE trial, half of the patients with PD showed disease progression within 8 weeks. Co-mutation or genomic alterations are possible causes of ineffectiveness, even if the patient has *BRAF* V600E; therefore, these patients can harbour other genomic abnormalities. The histological type of cholangiocarcinoma was also evaluated in another cohort of the ROAR study, which predominantly comprised intraductal cholangiocarcinomas, with two cases of extrahepatic cholangiocarcinoma.^11^^,^^23^ In both instances of extrahepatic cholangiocarcinoma, the best overall response within 16 weeks was stable disease, indicating that a degree of efficacy can be achieved even in the presence of extrahepatic cholangiocarcinoma.

This study was conducted based on public needs, providing a treatment opportunity for patients post-CGP in Japan, where there is no compassionate-use system. Therefore, the study was performed in a setting close to clinical practice, and the treatment was administered at the discretion of the investigator based on the indication on the package insert. However, we emphasize the collection of ample genomic data and recommend conducting tests every 12 weeks, ideally after the 16-week mark.

Patients with genomic mutations other than *BRAF* V600E also showed no response in the current study; one patient had a class 2 mutation and was not evaluable for efficacy, and the V600R case was PD. This study has a few limitations. First, because the study included all solid tumours, there were not enough patients with mutations other than *BRAF* V600E to fully evaluate the efficacy of the combination of dabrafenib and trametinib in this patient population. In particular, this was because the genomic aberrations used in the transplants were determined by a molecular tumour board designated by approximately 45 hospitals in Japan. Exclusion was based on this biased enrolment toward *BRAF* V600E cases, for which there is more evidence, and other V600 mutations (K, D, M, etc.) were not included by chance. As a result, the effect of combination therapy with dabrafenib and trametinib in patients with non-V600 mutations cannot be thoroughly examined. Further investigations are necessary to determine the efficacy of dabrafenib and trametinib against genomic abnormalities other than the *BRAF* V600E mutation. Second, we did not collect tissue samples before the start of treatment or at the time of disease progression. Therefore, the primary and acquired resistance could not be examined. Finally, a strength of this study is that it includes some cancer types not reported in previous studies; however, the number of cases was small, with only three cases of malignant soft-tissue tumours, cancer of unknown primary, and breast cancer. Considering the small number of cases, it would not be appropriate to discuss whether the dabrafenib–trametinib combination therapy could be effective in these cancer types. Further treatment experience is needed to check whether this combination therapy is effective in all *BRAF* V600E mutation-positive solid tumours not included in previous clinical trials as well as in this study.

The dabrafenib–trametinib combination therapy is also used in the adjuvant setting in melanoma and has promising long–term activity.^25^, ^26^, ^27^, ^28^ This implies a potential role for this therapy in other cancer types in the adjuvant setting.

This study represents a significant advancement in the identification of patients suitable for molecular targeted therapy in *BRAF* V600-associated cancers. It serves as a foundation for future research dedicated to addressing these cancers, which currently lack effective standard treatment options. The added indications relevant to Japan can further define the potential benefits of dabrafenib and trametinib in this patient population.

## Contributors

The study was designed by the funder in collaboration with all the authors. T-shimoi, KS, YA, NO, T-shibata, KN, and NY contributed to the conceptualisation, methods, and visualisation of the results of the study. T-shimoi, KS, YA, NO, T-shibata, KN, and NY contributed to the data curation. Data were analysed by T-shimoi and T-shibata. T-shimoi, T-shibata and NY contributed to the underlying data verification. All authors contributed to the investigation. YA, NO, and KN administered the project. All authors had access to all the data reported in the study and contributed to data interpretation. The first draft of the manuscript was written by T-shimoi. All authors reviewed and edited the manuscript, and approved the final manuscript for publication.

## Data sharing statement

The BELIEVE study team is committed to sharing with qualified external researchers access to patient-level data and supporting clinical documents from eligible studies. These requests are reviewed and approved by an independent review panel based on scientific merit. All data provided are anonymised to respect the privacy of patients who have participated in the trial, in line with applicable laws and regulations.

This trial data availability as per the criteria and process described on https://www.clinicalstudydatarequest.com.

## Declaration of interests

Dr. Tahara reports personal fees from Novartis, during the conduct of the study; grants and personal fees from Ono Pharmaceutical, grants and personal fees from Bayer, personal fees from MSD, personal fees from BMS, personal fees from Merck Biopharma, personal fees from Pfizer, personal fees from Rakuten Medical, personal fees from Lilly, personal fees from Boehringer Ingelheim, personal fees from Eisai, personal fees from Chugai Pharmaceutical, personal fees from Daiichi-Sankyo, personal fees from Janssen Pharmaceutical, personal fees from Genmab, personal fees from Astra Zeneca, personal fees from Abbvie, personal fees from Astellas, outside the submitted work.

Dr. Yamamoto reports payment or honoraria for lectures, presentations, speakers bureaus, manuscript writing or educational events from Ono Pharmaceutical, Chugai, Daiichi-Sankyo, Eisai, Payment for expert testimony from Eisai, Takeda, Boehringer Ingelheim, Cimic, Chugai, Other financial or non-financial interests from Astellas, Chugai, Eisai, Taiho, BMS, Pfizer, Novartis, Eli Lilly, AbbVie, Daiichi-Sankyo, Bayer, Boehringer Ingelheim, Kyowa-Hakko Kirin, Takeda, ONO, Janssen Pharma, MSD, MERCK, GSK, Sumitomo Dainippon, Chiome Bioscience, Otsuka, Carna Biosciences, Genmab, Shionogi, TORAY, KAKEN, InventisBio, Rakuten Medical.

## Appendix A Supplementary data

renamed dfc92

Supplementary Tables S1 and S2

Translational abstract
