# Supplementary material for: Dabrafenib and trametinib administration in patients with BRAF V600E/R or non-V600 BRAF mutated advanced solid tumours (BELIEVE, NCCH1901): a multicentre, open-label, and single-arm phase II trial
Source: eClinicalMedicine. 2024 Feb 2;69:102447. doi: 10.1016/j.eclinm.2024.102447 (PMC10850114; doi:10.1016/j.eclinm.2024.102447)
Supplement: renamed dfc92 [file mmc1.pdf]

Patient-proposed healthcare services

Prospective trial of patient-proposed healthcare services with multiple targeted agent based on the result of gene profiling by multigene panel test

NCCH1901

The prospective trial of patient-proposed healthcare services with multiple targeted agent based on the result of gene profiling by multigene panel test.

BELIEVE

Study Protocol

Study Representative Physician	Noboru YAMAMOTO<br>Department of Experimental Therapeutics, National Cancer Center Hospital<br>5-1-1, Tsukiji, Chuo-ku, Tokyo, JAPAN 104-0045<br>Tel: 03-3542-2511（direct line 7319）<br>Fax: 03-3542-3815<br>Email：nbryamam@ncc.go.jp
Study secretariat	Tatsunori SHIMOI<br>Department of Medical Oncology, National Cancer Center Hospital<br>5-1-1, Tsukiji, Chuo-ku, Tokyo, JAPAN 104-0045<br>Tel: 03-3542-2511（direct line 2331）<br>Fax: 03-3542-3815<br>Email: tshimoi@ncc.go.jp
	Kuniko SUNAMI<br>Department of Pathology and Clinical Laboratories, National Cancer Center Hospital<br>5-1-1, Tsukiji, Chuo-ku, Tokyo, JAPAN 104-0045<br>Tel: 03-3542-2511（direct line 7751）<br>Fax: 03-3542-3815<br>Email: ksunami@ncc.go.jp

July 2, 2019	Protocol, ver. 1.0
July 10, 2019	Protocol, ver. 1.1

November 21, 2019	Protocol, ver. 2.0
December 19, 2019	Protocol, ver. 3.0
April 23, 2020	Protocol, ver. 3.1
November 25, 2020	Protocol, ver. 4.0
April 22, 2021	Protocol, ver. 4.1
August 26, 2021	Protocol, ver 5.0
December 21, 2021	Protocol, ver 6.0
March 30, 2022	Protocol, ver 7.0
Jun 22, 2022	Protocol, ver 7.1
March 14, 2023	Protocol, ver 8.0
May 22, 2023	Protocol, ver 8.1
September 1, 2023	Protocol, ver 9.0

0. Synopsis

0.1. Flowchart

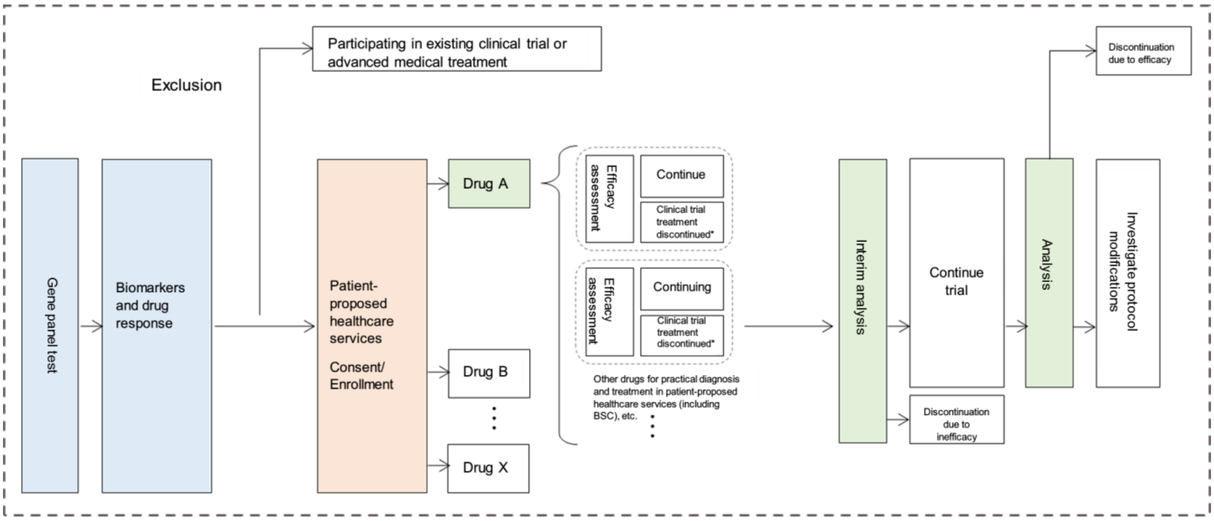

0.2. Purpose

For those patients who received a gene panel test that is covered by insurance in Japan or were evaluated for treatment and were found to have actionable genetic abnormalities, the objective is to administer off-label drugs corresponding to the respective genetic abnormalities based on the patient-proposed healthcare services system and to collect data on the treatment course.

Primary endpoint: Response rates in each drug cohort based on the best overall response in patients with measurable lesions up to 16 weeks after starting treatment

Secondary endpoints: Overall survival, progression-free survival, disease control rate, and incidence of adverse events

Perform subgroup analysis by cancer type, genetic mutation, and treatment as necessary.

0.3. Subjects

For those patients who received a gene panel test that is covered by insurance in Japan or were evaluated for treatment and were found to have actionable genetic abnormalities, and who wish to receive medicinal products through inclusion in this study based on the patient-proposed healthcare services system.

0.4. Treatment

This study includes medicinal products that are already covered by insurance in Japan for other malignant tumors, and does not include domestic unapproved drugs. In principle, the respective medicinal products are not included in this study if a sponsor-initiated clinical trial or an investigator-initiated clinical trial is being conducted to expand the indications, or if there is a plan to conduct such a trial soon. In addition, when a medicinal product that is newly covered by insurance for other malignant tumors is commercially available, etc., a confirmation with the medicinal product manufacturer/distributor is to be completed, and the list of medicinal products included in this study is to be updated twice a year as a guideline.

Joint consultations concerning actionable genetic abnormalities and medicinal products were conducted by three academic societies (the Japanese Society of Medical Oncology, the Japan Society of Clinical Oncology, and the Japanese Cancer Association), as reflected in "Clinical practice guidance for cancer diagnosis and treatment based on gene panel tests using a next-generation sequencer, etc. " and the "Evidence classification proposed by the expert panel standardization working group of the cancer genome-based healthcare promotion consortium steering committee" based on the determination of the expert panel investigating the result returns of the treatment options.

Based on the patient-proposed healthcare services system, the principal investigator or co-investigator will provide the patients who wish to be administered the medicines included in this study with an adequate explanation, including disadvantages such as adverse events, and such patients will then be enrolled in this study and treated after consent is obtained. The treatment dosage and administration of each medicinal product is as described in the package insert.

0.5. Expected number of subjects enrolled and study duration

The number of patients enrolled in this study will be affected by the frequency of actionable genetic abnormalities for each cancer type, the number of drugs to be included, and the availability from companies at no charge. However, it is ethically undesirable to expand the administration to a large number of patients with an inadequate exploratory examination of efficacy, so 50 patients with measurable lesions are to be measured per drug cohort, and an upper limit of 30 patients with no measurable lesions will be established, so that when that upper limit is reached, the need for revision of the protocol will be examined. If fewer than 50 patients with measurable lesions are enrolled at a time when 30 patients with no measurable lesions have been enrolled, enrollment will be continued with only patients having measurable lesions. Conversely, once 50 patients with measurable lesions have been enrolled, the enrollment of patients with no measurable lesions will end.

This study comprehensively handles a wide variety of cancer types and medications, and provides patients who are unable to participate in clinical trials, etc. with treatment options based on the patient-proposed healthcare services system, so the total number of enrollments is expected to be approximately several hundred cases per year.

Scheduled enrollment period: 5 years (scheduled from September 1, 2019 – August 31, 2024)
Tracking period: 3 years after completion of enrollment (Sept. 1, 2024 – Aug. 31, 2027)
Analysis period: 1 year (Sept. 1, 2027 – Aug. 31, 2028)
Total study period: 9 years (September 1, 2019 – August 31, 2028)

0.6. Contact information:

Study Representative
Physician Noboru YAMAMOTO
Department of Experimental Therapeutics, National Cancer Center Hospital
5-1-1, Tsukiji, Chuo-ku, Tokyo, JAPAN 104-0045
Tel: 03-3542-2511（direct line 7319）
Fax: 03-3542-3815
Email: nbryamam@ncc.go.jp

Study secretariat (inquiries relating to eligibility criteria and clinical criteria such as treatment, evaluation)

Tatsunori SHIMOI
Department of Medical Oncology, National Cancer Center Hospital
5-1-1, Tsukiji, Chuo-ku, Tokyo, JAPAN　104-0045
Tel: 03-3542-2511（direct line 2331）
Fax: 03-3542-3815
Email: tshimoi@ncc.go.jp

Kuniko SUNAMI
Department of Pathology and Clinical Laboratories, National Cancer Center Hospital
5-1-1, Tsukiji, Chuo-ku, Tokyo, JAPAN　104-0045
Tel: 03-3542-2511（direct line 7751）
Fax: 03-3542-3815
Email: ksunami@ncc.go.jp

Coordination office (inquiries relating to research operation)

Yayoi ANDO, Kanako KONDO, Sachie KAWABATA, Takako HITOMI, Natsuko OKITA, Kenichi NAKAMURA (manager)
Clinical Research Support Office, National Cancer Center Hospital
5-1-1, Tsukiji, Chuo-ku, Tokyo, JAPAN　104-0045
Tel: 03-3542-2511
Fax: 03-3542-3815
Email: NCCH1901_office@ml.res.ncc.go.jp

Table of Contents

0. SYNOPSIS 2

0.1. FLOWCHART 2
0.2. PURPOSE 2
0.3. SUBJECTS 2
0.4. TREATMENT 3
0.5. EXPECTED NUMBER OF SUBJECTS ENROLLED AND STUDY DURATION 3
0.6. CONTACT INFORMATION: 3

1. PURPOSE 8

2. BACKGROUND AND RATIONALE FOR RESEARCH PLAN 9

2.1. SUBJECTS 9
2.2. RATIONALE FOR ESTABLISHING THE RESEARCH PLAN 11
2.3. STUDY DESIGN 14
2.4. SUMMARY OF ADVANTAGES AND DISADVANTAGES EXPECTED FROM PARTICIPATION IN THIS STUDY 15
2.5. SIGNIFICANCE OF THIS RESEARCH 15
2.6. CONCOMITANT RESEARCH (INCLUDING SPECIMEN ANALYSIS RESEARCH) 16
2.7. ENROLLMENT TO PARTICIPATE IN PATIENT-PROPOSED HEALTHCARE SERVICES WITH MULTIPLE MEDICINES 16
2.8. RATIONALE FOR INCLUDING CHILDREN 15 YEARS OF AGE OR UNDER (STUDY PROTOCOL VER. 4.0) 16

3. CRITERIA AND DEFINITIONS USED IN THIS STUDY 17

3.1. GENE PANEL DEFINITIONS 17
3.2. ACTIONABLE GENETIC MUTATIONS 17
3.3. EVIDENCE LEVEL CLASSIFICATION 17
3.4. LABORATORY STANDARD VALUES IN CHILDREN UNDER 15 YEARS OF AGE 19

4. PATIENT SELECTION CRITERIA 19

4.1. ELIGIBILITY CRITERIA 19
4.2. EXCLUSION CRITERIA 20

5. ENROLLMENT 22

5.1. NEW PARTICIPATION PROCEDURE (IN THE CASE OF THE FIRST PATIENT IN EACH STUDY CENTER) 22
5.2. PATIENT ENROLLMENT (IN THE CASE OF THE SECOND AND LATER PATIENTS IN EACH STUDY CENTER) 22
5.3. PROCEDURE FOR THE COMPLETING ENROLLMENT FOR EACH DRUG 23

6. TREATMENT PLANNING AND TREATMENT CHANGE CRITERIA 24

6.1. PROTOCOL TREATMENT 24
6.2. CRITERIA FOR DISCONTINUING/TERMINATING THE PROTOCOL TREATMENT 25
6.3. POST-TREATMENT 26

7. EXPECTED ADVERSE EVENTS 27

7.1. ADVERSE REACTIONS EXPECTED WITH THE DRUG 27
7.2. EVALUATING ADVERSE EVENTS/ADVERSE REACTIONS 27

8. EVALUATION PARAMETERS, CLINICAL TESTING, AND EVALUATION SCHEDULING 29

8.1. PRE-ENROLLMENT EVALUATION PARAMETERS 29
8.2. TESTS AND EVALUATIONS DURING THE PROTOCOL TREATMENT PERIOD 30
8.3. TESTS AND ENDPOINTS WHEN THE PROTOCOL TREATMENT IS COMPLETED 31
8.4. FOLLOW-UP 31
8.5. STUDY CALENDAR 31

9. DATA ACQUISITION 33

9.1. CASE REPORT FORM（CRF）......33
9.2. CRF STORAGE......33

10. DISEASE, ETC. (ADVERSE EVENT) REPORTS......34

10.1. SERIOUS ADVERSE EVENTS AND EVENTS SUBJECT TO EMERGENCY REPORTS......34
10.2. REQUIREMENTS AND PROCEDURES FOR REPORTING TO THE STUDY REPRESENTATIVE PHYSICIAN......35
10.3. RESPONSIBILITIES OF THE STUDY REPRESENTATIVE PHYSICIAN/STUDY SECRETARIAT......36
10.4. RESPONSE TO THE PRINCIPAL INVESTIGATOR OF PARTICIPATING FACILITIES (INCLUDING THE RELEVANT FACILITY) 39
10.5. RESPONSE TO THE EFFICACY AND SAFETY EVALUATION COMMITTEE......39
10.6. REPORTS ON PREGNANCY AND OVERDOSE......39
10.7. SAFETY INFORMATION REPORTS TO THE COMPANY PROVIDING THE MEDICATION......40

11. EFFICACY ASSESSMENTS......40

11.1. EFFICACY ASSESSMENTS......40

12. STATISTICAL PARAMETERS......45

12.1. DEFINITION OF THE ANALYSIS SETS......45
12.2. DEFINITION OF THE ENDPOINTS......45
12.3. MAIN ANALYSIS AND JUDGMENT CRITERIA......46
12.4. INTERIM ANALYSIS......47
12.5. FINAL ANALYSIS......51
12.6. STUDY PERIOD AND NUMBER OF ENROLLMENTS......51
12.7. EARLY DISCONTINUATION......52

13. ETHICAL MATTERS......54

13.1. PATIENT PROTECTION......54
13.2. INFORMED CONSENT......54
13.3. PROTECTION OF PERSONAL INFORMATION AND PATIENT IDENTIFICATION......56
13.4. PROTOCOL COMPLIANCE......58
13.5. APPLICATION TO THE CERTIFIED CLINICAL RESEARCH REVIEW BOARD AND APPLICATION TO PATIENT-PROPOSED HEALTHCARE SERVICE EVALUATION COMMITTEE, NOTIFICATION OF THE IMPLEMENTATION PLAN......58
13.6. PERIODIC REPORT AND PERFORMANCE REPORT......58
13.7. PROCEDURES PERFORMED BY THE PRINCIPAL INVESTIGATOR AT EACH STUDY CENTER......59
13.8. RESEARCH CONDUCT APPROVAL FOR EACH STUDY CENTER......59
13.9. CHANGING THE PROTOCOL CONTENT......60
13.10. CONFLICT OF INTEREST (COI) RELATING TO THE RESEARCH STAFF......60
13.11. COMPENSATION......62
13.12. INTELLECTUAL PROPERTY......63
13.13. DISCLOSURE OF INFORMATION RELATING TO THIS RESEARCH......63

14. MONITORING AND AUDITS......64

14.1. PERIODIC MONITORING......64
14.2. AUDITS......65
14.3. MANAGING NONCOMPLIANCE......65

15. SPECIAL NOTES......68

15.1. ADDITIONAL STUDY CENTERS......68
15.2. DETERMINATION OF WHETHER OR NOT TO CONTINUE WITH PATIENT-PROPOSED HEALTHCARE SERVICE......68

16. RESEARCH ORGANIZATION......69

16.1. SOURCE OF FUNDING FOR THIS RESEARCH......69
16.2. STUDY REPRESENTATIVE PHYSICIAN......69
16.3. STUDY SECRETARIAT......69

16.4. PARTICIPATING FACILITIES (STUDY CENTERS) ... 69
16.5. MATTERS CONCERNING PERSONS ENGAGED IN CLINICAL RESEARCH OTHER THAN THE PRINCIPAL INVESTIGATOR ... 70

17. DEVELOPMENT OF RESEARCH RESULTS AND COMPLETION OF THE STUDY ... 72

17.1. PUBLICATIONS AND PRESENTATIONS AT PROFESSIONAL CONFERENCES ... 72
17.2. PRIMARY ENDPOINT REPORT/FINAL REPORT ... 72
17.3. COMPLETING THE STUDY ... 73

18. LITERATURE CITATIONS ... 73

19. APPENDICES ... 74

1. Purpose

For those patients who received a gene panel test that is covered by insurance in Japan or were evaluated for treatment and were found to have actionable genetic abnormalities, the objective is to administer off-label drugs corresponding to the respective gene abnormalities based on the patient-proposed healthcare services system and to collect data on the treatment course.

Primary endpoint: Response rates in each drug cohort based on the best overall response in patients with measurable lesions up to 16 weeks after starting treatment

Secondary endpoints: Overall survival, progression-free survival, disease control rate, and incidence of adverse events

Perform subgroup analysis by cancer type, genetic mutation, and treatment as necessary.

2. Background and Rationale for Research Plan

2.1. Subjects

2.1.1. Epidemiology

According to the latest “2017 Cancer Statistics,” the number of cancer cases in Japan was approx. 1,014,000 in 2017(1). Of these, approximately 53,000 cases have been reported of hematologic tumors such as malignant lymphoma, leukemia, and multiple myeloma, and the number of cases of solid tumors excluding these hematologic tumors is estimated to be 950,000 or more.

In addition, based on the same report, 16.2% of all patients with cancer in the regional cancer registry from 2006 to 2008 involve cancer associated with distant metastasis. Assuming that the number of cases of solid tumors associated with similar distant metastasis is more than 950,000, it is estimated that every year there are approximately 150,000 cases involving solid tumors in which new distant metastasis occurs. Patients who have solid tumors associated with such distant metastases are extremely difficult to cure and their prognosis is poor. Except for breast, uterus, prostate, and thyroid cancer, such patients have extremely poor 5-year relative survival rates of 18% or less.

This study is targeted at patients who received a gene panel test that is covered by insurance in Japan, or were performed as Evaluation treatment, but among those, only the ones in whom a solid tumor has developed. Solid tumors can be divided into common cancers and rare cancers. This study includes solid tumors in general for which standard treatment has been completed, but in particular rare cancers with metastasis/recurrence or cancers of unknown primary where there is no standard treatment are to be included from the time of diagnosis.

“Rare cancer” is defined in Europe as a “malignant tumor with annual incidence of fewer than 6 cases per 100,000 population” and in the US as a “malignant tumor with annual incidence of fewer than 15 cases per 100,000 population” （http://www.rarecare.eu/）(2). The Japan Ministry of Health, Labour and Welfare's “Rare Cancer Medical Services/Support Study Group” was established in March 2015 to address the issue of rare cancers as [1] there are generally fewer than 6 cases per 100,000 population, and [2] because the number is small, the problems with being diagnosed and receiving treatment are substantial compared to other type of cancers [2015 Rare Cancer Medical Services/Support Study Group Report]. The same analysis as reported by RARECARE in Europe was performed using the Japanese regional cancer registry, with the result that the proportion of rare cancers among all types of cancer in Japan was 15%(3).

Furthermore, various molecular biology-related searches have been conducted in recent years, and not only have rare tissue subtypes been histologically identified among cancer types that have hitherto been referred to as common cancers, but the rare fractions identified have also been defined in terms of molecular biology, and a tumor reclassification is in progress(4).

2.1.2. Cancer genome-based healthcare policy in Japan and cancer gene panel tests

The development of genome-based healthcare in recent years has promoted discussions of cancer genome-based healthcare in Japan centering on the Ministry of Health, Labour and Welfare (https://www.mhlw.go.jp/file/05-Shingikai-10901000 -Kenkoukyoku-Soumuka/0000169236.pdf). In this connection, genome information is analyzed and effectively accumulated to implement cancer genome-based healthcare that can provide medical treatment that is tailored to the patient's individual conditions based on the latest medical knowledge, and the construction of a system is shown to be utilized for the development of diagnosis and treatment with new medicines, etc. Among these, four necessary functions and roles have been worked out. These are: [1] designation of cancer genome-based healthcare core base hospitals and cancer genome-based healthcare cooperating hospitals, [2] establishment of cancer genome information management centers (C-CAT), [3] construction of a genome analysis and cancer knowledge database, and [4] promotion of strategic research and development. In fact, as of November 1, 2020, MHLW has designated 12 cancer genome-based healthcare core base hospitals, 33 hospitals cancer genome-based healthcare hospitals, as well as 161 cancer genome-based healthcare cooperating hospitals (5).

The background to the feasibility of implementing cancer genome-based healthcare is largely due to

the progress in genome analysis technology. The human genome project, which began in 1990, required 13 years for whole genome analysis of one person, but in 2016 it became possible to analyze a genome in less than one week using a next-generation sequencer (NGS). As of the beginning of 2019, two cancer gene panel tests have received regulatory approval, and are covered by insurance as of June 2019. Furthermore, two cancer gene panel tests are available to patients who have been enrolled for advanced medical treatments.

The cancer gene panel test can test for dozens to millions of genome abnormalities at once using a cancer tissue specimen. Research in clinical trials, etc. includes a companion test for genome abnormalities involved in the selection of a therapeutic agent for a malignant tumor (molecular target therapeutic agent) that has already been covered by insurance, and as a complementary test to assist in selection, and includes genome abnormalities related to the feasibility of treatment and those related to prognosis and diagnosis.

2.1.3. Standard treatment by disease stage and summary of prognosis, prognostic factors/predictors

As solid tumors contain a variety of cancer types, the mention of standard treatment and prognosis by stage is omitted in individual cancer types. For solid tumors with metastases/recurrences, median survival is often around 1-3 years. Median survival is often less than one year, particularly in patients with solid tumors who have had metastases/recurrences and who have completed standard treatment. Although clinical stages and pathological classifications are used to determine the treatment plan, it is still difficult in many cases to predict the therapeutic effect. In recent years with the development of molecular-targeted therapeutic agents, treatment selection has come to be performed based on cancer genome abnormalities as an effect predictor. Tests for these genome abnormalities are often regarded as biomarkers (companion tests, complementary tests) involved in the selection of molecular target therapeutic agents.

2.1.4. Basis for study population selection

To administer off-label drugs that correspond to the biomarkers for individual patients in this study, we will focus on patients for whom the results of gene panel testing by next-generation sequencer are available at enrollment time.

Because it is deemed necessary for the accuracy of the gene panel test itself to be guaranteed to a certain degree, it is a requirement that the gene panel test used in this study is covered by insurance or is implemented as an evaluation treatment. The biomarkers targeted by this research were jointly issued by three academic societies (the Japanese Society of Medical Oncology, the Japan Society of Clinical Oncology, and the Japanese Cancer Association), as reflected in “Clinical practice guidance for cancer diagnosis and treatment based on gene panel tests using a next-generation sequencer, etc.”(6) and the “Evidence classification proposed by the expert panel standardization working group of the cancer genome-based healthcare promotion consortium steering committee.”

Recently, with pembrolizumab having received insurance coverage for cross-organ microsatellite instability high (MSI-H) solid cancers(7) in the US and Japan, and with the ongoing development of NTRK inhibitors (larotrectinib, entrectinib) for use in cross-organ cancers with NTRK gene fusion gene abnormalities(8), etc., it has come to be seen that recent molecular-targeted therapeutic agents not limited by the type of cancer are being developed as combinations of biomarkers and their associated therapeutic agents. In this study, regardless of the type of cancer, medicines not covered by insurance are to be used for patients who have biomarkers that indicate the molecular-targeted medicines are expected to have therapeutic effects.

The dosage and administration of the drugs to be used for childhood cancers are often unknown. Nevertheless, the situation is the same for childhood cancers as for adults with rare cancers, as there are only a few patients and the drug dose per person is low, so there is little profit for pharmaceutical companies and the pediatric population has been excluded in many clinical trials. Therefore, it is important to gather information on experience with the use of medicines in the pediatric population, and it is necessary to pursue the development of pediatric cancer treatments, childhood cancewrs are included in this study.

Recently, the United States reported aggregated results of actionable abnormalities that were analyzed by performing cancer gene panel tests on 12,670 specimens obtained from 11,369 cancer patients(9). According to the report, approximately 38% of patients had clinically significant genetic abnormalities (actionable mutations). According to the breakdown, 7% of biomarkers corresponded to drugs already approved by the US Food and Drug Administration (FDA), 2% of biomarkers corresponded to standard treatments, 9% of biomarkers corresponded to drugs already approved for other tumors, and 18% are abnormalities that are expected to correspond to future biomarkers. Although the types of gene panel tests differ in Japan, we assume that the percentage of biomarkers that can be found to correspond to drugs already approved for other tumors, which are the target of this study, will be approximately 9% of the patients who received the cancer gene panel test.

Although the drugs used in this study are those for which pharmaceutical approval or insurance applicability has been obtained for any cancer type, we assume there is almost no data on administration for the cancer type targeted for medical treatment. In addition, because there are no standard treatments or standard treatments have already been performed, such patients will be included as subjects in this study at the stage where there is no effective treatment. Therefore, if examples of a certain response to administration of an off-label drug corresponding to a biomarker can be confirmed in this research, it will provide a basis for the existence of a treatment effect that does not depend on cancer type, and it is expected to lead to the development of further treatments including sponsor-initiated clinical trials or investigator-initiated clinical trials.

2.2. Rationale for Establishing the Research Plan

2.2.1. Previous research overseas in making biomarker-based treatment selections

According to the US FDA, a biomarker is defined as “a parameter that is objectively measured and evaluated as an indicator of a normal process, pathological process, or pharmacological response to treatment.”

The definition of “biomarker” in this study includes gene mutations/fusion genes/gene amplifications/gene deficiencies that can be searched through a gene panel test that is covered by insurance in Japan, or for which an evaluation for treatment has been carried out.

The concept of “personalized medicine” has been proposed, and further significant progress in life science research in recent years has led to the active detection of inherent genetic abnormalities in individual patients and the corresponding molecular-targeted therapies. The substantial implementation of integrative omics research including genomic analysis is gradually enabling personalized medicine in, for example, the major/minor BCR-ABL fusion gene in imatinib, the HER2 gene amplification in trastuzumab, the EGFR gene activation mutation in gefitinib, the ALK fusion gene in crizotinib, the KRAS/NRAS gene activation mutation in cetuximab, and the like. Although these involve patient stratification based on a single piece of omics information, it is possible with the developments in genetic analysis technology to screen for many molecular abnormalities at one time using NGS, etc., and the molecular biological characteristics of each tumor can be more precisely and accurately understood, at least to some extent, in what is referred to as “high-precision medicine/optimized medicine.”

Several clinical trials based on gene screening have been conducted overseas. Representative examples include “basket-type trials” (a type of trial in which the subjects are determined based not on the cancer type but on biomarkers), including the Molecular Analysis for Therapy Choice (NCI-MATCH) and Molecular Profiling-based Assignment of Cancer Therapeutics (MPACT) of the US National Cancer Institute (NCI), and “umbrella-type trials” (a type of trial in which biomarkers are measured, and patients are enrolled into multiple trials based on these results), including the Investigation of Serial Studies to Predict Your Therapeutic Response with Imaging and Molecular Analysis 2 (I-SPY 2), the Biomarker-integrated Approaches of Targeted Therapy for lung cancer elimination (BATTLE), and the Lung Master Protocol (Lung-MAP). In particular, only the Targeted Agent and Profiling Utilization Registry Study (TAPUR study) is recruiting patients regardless of the type of cancer.

This trial can be said to be a mixed-type study with elements of both the umbrella- and basket-types in the sense that subjects are determined based on biomarkers in a wide target range of solid tumors without limiting the cancer types.

Table 2.2.1a: Basket Trial and Umbrella Trial Examples

	Status	No. of test centers	Design	Trial period	Sub-trials	Target disease	[1] Primary endpoint [2] Secondary endpoints
LUNG-MAP	Open to enrollment	≥ 700	Umbrella Phase II/III	≥ 8 years	4-7	Advanced squamous cell lung cancer	[1] PFS [2] RR, adverse events
BATTLE-1	Completed	1	Umbrella Phase II Adaptive	9 yrs	4	Advanced NSCLC	[1] 8-week DCR [2] RR, OS, TTP, adverse events, biomarkers, PK
BATTLE-2	Open to enrollment	2	Umbrella Phase II Adaptive	6 yrs	4	Advanced NSCLC	[1] 8-week DCR
I-SPY 2	Open to enrollment	23	Umbrella Phase II Adaptive	≥ 5 years	≥ 8	Local advanced breast cancer	[1] 26-week pCR [2] biomarkers, RFS, OS, adverse events
NCI MATCH	Open to enrollment	Approx. 2400	Basket Phase II	Unknown	20-25	Advanced solid cancers or lymphoma	[1] RR [2] PFS
NCI MPACT	Open to enrollment	1	Basket Phase II	4 yrs	4	Advanced solid cancer	[1] 16-week RR and/or PFS
FOCUS4	Before beginning	Unknown	Umbrella Phase II/III	Unknown	5	Advanced/metastatic Colorectal cancer	[1] PFS
TAPUR	Open to enrollment	113	Basket Phase II			Advanced solid cancer B-cell lymphoma Multiple myeloma	[1] 16-week ORR/SD [2] PFS, OS, Duration of treatment, adverse events

Table 2.2.1b: Examples of the Diagnostic and Treatment Methods in Basket Trials and Umbrella Trials

	Diagnostic and treatment methods
LUNG-MAP	Archival or fresh tumor biopsy specimens: NGS, IHC or other assay method as required
BATTLE-1	Fresh tumor biopsy specimens: EGFR mt/cn, KRAS/BRAF mt, VEGF/VEGF-R expression, RXR/cyclin D1 and CCND1 cn
BATTLE-2	Fresh tumor biopsy specimens: Predictive biomarker assays for KRAS mt, EGFR, PI3K/AKT, and MEK inhibitors
I-SPY 2	Fresh tumor biopsy specimens: ER with MammaPrint and IHC; and ERBB2 with PgR, IHC, FISH or TargetPrint
NCI MATCH	Fresh tumor biopsy specimens: NGS, IHC or FISH as required, searching for approximately 200 genes
NCI MPACT	Fresh tumor biopsy specimens: gene mt and amplification associated with DNA repair, PI3K, and RAS/RAF pathways
FOCUS4	Tumor biopsy specimens: MMR and PTEN with IHC, BRAF, PIK3CA, KRAS, NRAS mt, epiregulin mRNA
TAPUR	NGS, IHC or other assay method as required

2.2.2. Actionable genetic abnormalities

Genetic abnormalities that can be analyzed by gene panel testing include base substitution mutation, insertion/deletion mutation, copy number abnormality, and rearrangement, and amplification and fusion mainly result in activation. Whether or not a mutation is significant depends on which base changes have occurred. Genetic abnormalities that can aid in these therapeutic strategy decisions can be classified by the respective level of evidence. For example, in the evidence classification outlined in the “Clinical practice guidance for next-generation sequencing in cancer diagnosis and treatment (Ver. 2.1)” currently published by the Japanese Society of Medical Oncology, the Japanese Society of Clinical Oncology and the Japanese Cancer Association, the evidence level is defined to A to F, and those classified as evidence levels A to C relate to consideration of the use of clinical trials and advanced medical treatments, etc. for evidence-based treatment choices (10). In addition, for genetic abnormalities with an evidence level of D, “The scientific basis is insufficient, but evaluations such as clinical trials, advanced medical treatments, off-label use of drugs on the NHI drug price listing, in patient-proposed healthcare services, etc., and concomitant medical care expenses not covered by insurance, etc. will be considered under the consensus of the expert panel.”

Therefore, in this study, "a genetic abnormality for which the result return as a treatment option is determined appropriate by the expert panel" is defined as an "actionable genetic abnormality."

2.2.3. Medicines that can be used in this study

This study includes medicinal products that are already covered by insurance for use in other malignant tumors. It does not include domestic unapproved drugs. The specific drug list is shown in the Appendix.

Inquiries are directed to the manufacturers/distributors of the corresponding drugs to see if a sponsor-initiated clinical trial or an investigator-initiated clinical trial is being conducted to expand the indications, or if such a trial is planned, and if so, in principle, the respective medicinal products are not to be included in this study. In addition, inquiries are also made when a medicinal product that is newly covered by insurance for other malignant tumors becomes commercially available to see whether it should be included in this study. The drug list will be updated based on this information and the results of the interim analysis,, and each time the Study Representative/Study Secretariat will revise the contents of the protocol (13.9.).

Drugs can be provided free of charge or can be paid for by the patient. Companies that manufacture or distribute the drug, or intend to manufacture or distribute it, are required to manage conflicts of interest under the Clinical Research Act as "a company, etc. related to the present research" (13.10.).

2.2.4. Drug selection for individual patients

The selection of a treatment corresponding to the actionable genetic abnormality will be determined by the investigator or co-investigator in consultation with the patient based on the examination results of the expert panel. With these results, and based on the patient-proposed healthcare services system, once the principal investigator or co-investigator has provided the patients who wish to be administered the medicines included in this study with an adequate explanation, including the administration schedule for each drug, and disadvantages such as adverse events, the final drug selection will be made and consent will be obtained from the patients for their participation in this study and for the drug selected. When there are multiple drug candidates, the selection will be made in consideration of patient’s concomitant diseases, the cancer type and tissue distribution of the corresponding drug, costs, and patient's inclination, etc.

2.2.5. Post-treatment

In the present study, the post-treatment after discontinuing the protocol treatment is not particularly defined, but the following post-treatment can be assumed.

1. Participation in patient-proposed healthcare services for medications for other different genetic abnormalities obtained from the initially submitted specimens
2. Participation in patient-proposed healthcare services for the same type of medication for the same genetic abnormalities

3. Conducting some form of chemotherapy for practical diagnosis and treatment
4. Participation in other clinical trials and studies
5. Follow-up/best supportive care, etc.

2.3. Study design

2.3.1. Research summary

For those patients who received a gene panel test that is covered by insurance in Japan, or were evaluated for treatment and were found to have actionable genetic abnormalities, the objective is to administer off-label drugs corresponding to the respective genetic abnormalities based on patient-proposed healthcare services system and to collect data on treatment course.

In this study, off-label drugs are used for which insurance coverage has not been obtained. For this reason, it corresponds to specific clinical research conducted under the Clinical Research Act based on the patient-proposed healthcare services system. The medicines are provided free of charge from the distributors based on the contract, or the drug costs will be paid by the patient. If the drug supply is received without cost, the company providing the drug can receive the information on patient background, treatment effects, and safety data that is collected in this study based on the contract.

In this study, the National Cancer Center Hospital will assume responsibility for the Study Representative Physician and the overall coordination office, and the cancer genome-based healthcare core base hospitals will participate as multi-institutional joint research facilities recognized as collaborative medical institutions for the patient-proposed healthcare services.

2.3.2. Design of planned next trial

This study will enable the collection and analysis of cross-organ cross-sectional clinical data for populations carrying each biomarker. If a promising result is obtained for a drug's therapeutic effect on the biomarkers in this patient-proposed healthcare service approach, a sponsor-initiated clinical trial or an investigator-initiated clinical trial will be conducted in consultation with the drug manufacturer/distributer, or a proposal will be made to the "Study Group on Unapproved and Off-label Drugs of High Medical Need" with the aim of leading to a subsequent expansion of the indications and insurance coverage for these medicines. If necessary, it might be possible to evaluate the efficacy against specific molecular targets in larger scale trials.

2.3.3. Rationale for establishing the endpoints

This study targets a patient population not included in trials, and there is no data on a combined therapeutic effect or prognosis for each of these medicines. Accordingly, in this study, it is significant to examine the background factors, course of treatment, efficacy, etc., of the target population from various perspectives according to cancer type, biomarkers, and medication. For this reason, the endpoints in this study are as follows.

- Primary endpoint: Response rates in each drug cohort based on the best overall response in patients with measurable lesions up to 16 weeks after starting treatment
- Secondary endpoints: Overall survival, progression-free survival, disease control rate, and incidence of adverse events

Subgroup analysis will be performed by cancer type, genetic mutation, and treatment depending on the number of patients registered. The types and frequency of treatment regimens will also be explored by cancer type/gene abnormality and biomarkers.

2.3.4. Clinical hypothesis

The main research hypothesis of this study was: "For patients who have been subjected to genetic profiling by gene panel testing, a patient-proposed healthcare service approach will match patients with one of the medicines that targets biomarkers, and the response drug will be obtained for the patients who

have been enrolled." This study explores this hypothesis.

2.3.5. Central pathology review

In this study, if it is confirmed that the pathology diagnosis criteria for the respective study center have been met and the case is eligible, no central pathology review will be performed. Moreover, the protocol will be revised if a central pathology review is required depending on the circumstances of the study center.

2.3.6. Central efficacy review

No central review of efficacy (tumor response) will be performed in this study.

2.4. Summary of advantages and disadvantages expected from participation in this study

2.4.1. Expected advantages

This study, based on the patient-proposed healthcare service system, is intended for patients for whom there is no standard treatment or for whom standard therapy has already been given, and since such patients have only had systemic treatment with low levels of evidence, patients are expected to benefit from the addition of new treatment options through participation in this study.

2.4.2. Expected risks and disadvantages

Possible disadvantages of participating in this study include adverse events and losing the opportunity of getting enough palliative care. At the time of entry into this study, a patient should be given sufficient information, including information about these disadvantages, and the patient's consent should be obtained.

2.5. Significance of this research

"Conventional medicine" refers to the confirmation of the name of a disease based on general medical information (medical interviews, physical findings, biochemical tests, histopathology examinations, etc.), and the implementation of the corresponding standard treatment. In this case, the condition of the disease varies from patient to patient and little consideration is given to the individual constitution of the patient, and while a "standard treatment" with the associated side effects is indicated, that is not necessarily correct.

A mechanism to develop a drug that can be expected to have therapeutic effects based on biomarkers, as in this research, is extremely important from the viewpoint of satisfying these unmet medical needs. The significant progress in life science research in recent years has made it possible to detect a large number of genetic abnormalities (mutations, amplifications, fusions, etc.) at one time with NGS, etc. at extremely low cost and quickly. Biomarker-based clinical research enables the targeting of rare cancers using these technologies, which will lead to the establishment of a system for providing "optimized medicine" (precision medicine) to individual patients.

By conducting this study, we can provide an opportunity for the use of promising drugs in patients with cancer types for which therapeutic development is challenging. From the standpoint of strictly pursuing the scientific nature of clinical trials, the basic approach is to narrow down the target disease and simultaneously design and carry out a large number of studies on a single medicine, although it is virtually impossible to conduct therapeutic development with such an approach in disease areas where therapeutic development is challenging. Although this research can only provide exploratory results, the burden of patient enrollment and research implementation can be reduced by adopting a research form that will serve the recipients in areas where treatment development is challenging. This leads to an increased likelihood of providing promising drugs to patients with diseases in areas in which therapeutic development is not progressing. Also, an approach that examines multiple treatment options in a single test and assigns patients to treatments that appear to be the most effective through consideration of the significance of the biomarkers can promote multiple drug development via a common platform, and can more quickly provide patients with safer and more effective treatments.

On the other hand, so as not to impede drug development based on a system closer to pharmaceutical approval such as a sponsor-initiated clinical trial or an investigator-initiated clinical trial being conducted in collaboration with a drug development company, or a clinical trial scheduled to be conducted in the near future, patients who are members of the target population in the aforementioned trials will not be included as subjects in the current study, which will thus be operated so as not to conflict with the company's development policy.

Through this research, we aim to establish a regulatory scheme for drug approval through the development of organ-specific and cross-organ, biomarker-based treatments. Also, by implementing an attractive basis in this research, we will create a better environment in which pharmaceutical companies in Japan can more readily pursue therapeutic development.

2.6. Concomitant research (including specimen analysis research)

At the planning stage of this research, there is no plan for concomitant research. If, in a study other than the present research, a newly collected specimen or an existing specimen is to be used in future work, patient consent to include this use will be newly obtained.

2.7. Enrollment to participate in patient-proposed healthcare services with multiple medicines

Patients who participate in treatment within another drug cohort that is included in this study will be given the additional explanation, give consent again, and be enrolled again.

2.8. Rationale for adding children 15 years of age or under (Study Protocol Ver. 4.0)

The cancer gene panel test is now covered by insurance for pediatric patients and is currently used in routine medical care. According to a report from the U.S., 43% of patients with malignant solid tumors between the ages of 1 and 22 years who underwent gene panel testing had actionable genetic abnormalities (11), and providing biomarker-based therapy to pediatric patients in Japan will also become essential. In recent years, the simultaneous development of molecular target drugs for children and adults has been recommended on the basis of scientific evidence (12). Expanding this study, which has hitherto been conducted in patients aged 16 years and older, to include pediatric patients will increase the likelihood of being able to provide treatment options for rare cancers, including pediatric cancers, and cancer types for which treatment development is proving difficult in Japan, and will serve as a framework for future drug development.

3. Criteria and definitions used in this study

3.1. Gene panel definitions

This study targets the gene panel tests listed below that are covered by insurance in Japan, or have been used in treatment assessments. If a new gene panel test is approved during the study period, the content of the protocol will be revised (13.9) to accommodate it.

Covered by insurance (including during the treatment evaluation period)

- OncoGuide™ NCC Oncopanel system (approval code 23000BZX00398000)
- FoundationOne®CDx cancer genome profile (approval code 23000BZX00403000)
- FoundationOneLiquid® CDx cancer genome profile (approval code 303000BZX74000)
- Guardant360® CDx liquid biopsy test (approval code 30300BZX00345000)
- GenMineTOP Cancer Genome Profiling System (approval code 30400BZX00155000)

Advanced medical treatment B

- Multiplex gene panel test (OncoGuide™ NCC Oncopanel system)
  Requesting study center: National Cancer Center Hospital
- Multiplex gene panel test (Todai OncoPanel)
  Requesting study center: Tokyo University Medical School Hospital
- Multiplex gene panel test (Oncomine™ Target Test)
  Requesting study center: Osaka University Medical School Hospital
- Multiplex gene panel test (TruSight Oncology 500)
  Requesting study center: Okayama University Hospital
- Multiplex gene panel test (FoundationOne®CDx cancer genome profile)
  Requesting study center: Kyoto University Hospital
-

3.2. Actionable genetic mutations

In this study, a "genetic abnormality with an evidence level of D or higher (A, B, C, D) for which the treatment option result return is determined to be appropriate" by the expert panels of each cancer genome-based healthcare designated core base hospital and cancer genome-based healthcare hospital in accordance with the “Guidelines for the Development of Cancer Genome-Based Healthcare Core Base Hospitals,” or equivalent genetic abnormality, is defined as an "actionable genetic abnormality."

However, since A is usually a “biomarker (gene abnormality) approved as a companion diagnostic drug for the relevant cancer type,” drugs corresponding to A are not target drugs in this study.

3.3. Evidence level classification

Genetic abnormalities and the corresponding drugs expected to be targeted in this study are assumed to be classified according to criteria A–D for the evidence level classifications regarding the treatment efficacy in Table 3.3. (Excerpt from “Clinical practice guidance for next-generation sequencing in cancer diagnosis and treatment (Ver. 2.1))”)

Table 3.3 Revised Draft of the Criteria for Classifying the Level of Evidence on Therapeutic Effects (see: "Clinical practice guidance for next-generation sequencing in cancer diagnosis and treatment (Ver. 2.1, Ver. 1.0)," Japanese Society of Medical Oncology, the Japanese Society of Clinical Oncology, and the Japanese Cancer Association))

Criteria	Ver. 2.1	Criteria details	Ver. 1.0
Drug exists with domestic approval for the relevant cancer type	A	Drug exists with domestic approval for cancer type and biomarkers relevant to the indication	1A
Drug exists with FDA approval for the relevant cancer type	A	Drug exists with FDA approval for cancer types and biomarkers relevant to the indication	1B
Guideline is described for the relevant cancer type	A	Guideline is described for the use of the drug for cancer types and biomarkers relevant to the indication	1B
Consensus among experts and with meta-analyses/clinical trials with high statistical reliability for the relevant cancer type	B	Consensus among experts and with support from meta-analyses/clinical trials with high statistical reliability for the use of the drug for cancer types and biomarkers relevant to the indication	2A
Drug exists with domestic or FDA approval for other cancer types	C	Drug exists with domestic or FDA approval for other cancer types and for biomarkers relevant to the indication	2B
Consensus among experts and with meta-analyses/clinical trials with high statistical reliability for other cancer types	C	Consensus among experts and with support from meta-analyses/clinical trials with high statistical reliability for the use of the drug for other cancer types and for biomarkers relevant to the indication	—
Efficacy demonstrated in small-scale clinical trials regardless of cancer type	C	Efficacy demonstrated in small-scale clinical trials for the use of the drug for biomarkers relevant to the indication regardless of cancer type	—
Efficacy demonstrated in case reports regardless of cancer type	D	Efficacy demonstrated in case reports for the use of the drug for biomarkers relevant to the indication regardless of cancer type	3A
Efficacy (*in vitro* or *in vivo*) reported in preclinical trials	E	Efficacy (*in vitro* or *in vivo*) reported in preclinical trials for the use of the drug for biomarkers relevant to the indication regardless of cancer type	3B
Known to be related to malignant transformation	F	Relevant biomarkers are known to be related to malignant transformation	4
Known to be related to drug resistance	R	Relevant biomarkers are known to be related to drug resistance	—

3.4. Laboratory standard values in children under 15 years of age

For standard values of clinical tests in children under 15 years of age, use the “Pediatric Laboratory Test Reference Ranges” (Attached Table 1).

For items not listed in the “Pediatric Laboratory Test Reference Ranges,” use the JCOG Common Standard Reference Ranges (Attached Table 2). For items not listed in either of the above, use in-house reference values.

4. Patient selection criteria

4.1. Eligibility criteria

Patients who satisfy all of the following eligibility criteria will be enrolled as eligible cases

1） Patients who are diagnosed with a solid tumor by histologic diagnosis (including cancers of unknown primary)

※ If the histopathology testing is performed by another facility, the pathology specimen must be ordered and confirmed to meet the criteria in the pathology diagnosis of the study center

2） Patients with incurable, unresectable, progressive lesions (metastatic and/or local progression), which can be any of the following i) or ii) (any number of pretreatment regimens):

i） No standard treatment (or no treatment according to standard treatment) is available

ii） When there is a standard treatment (or a treatment according to standard treatment) available, the relevant standard treatment has been interrupted for lack of efficacy or toxicity

3） There are no age requirements for patients at the time of enrollment. However, in the case of oral preparations patients must be capable of receiving the dosage form as stated in the package insert.

4） Within 28 days prior to enrollment, patients must have a tumorous lesion confirmed by contrast CT[*1] or MRI[*1] (head[*2], chest, abdomen, pelvis; slice thickness ≤ 5 mm) (presence of any type of measurable lesion[*3])

*1: Simple CT or MRI is acceptable if allergy to the contrast agent, renal dysfunction, or bronchial asthma are present

*2: Only if a brain tumor or brain metastasis is present

*3：See details in section 11.1.2 of the Protocol

5） Patients who received a gene panel test that is covered by insurance in Japan or were evaluated for treatment and were determined to have actionable genetic abnormalities

6） The patient has documents to prove the examination results of the expert panel examining actionable genetic abnormalities and treatment options based thereon (including copies of reports, medical records, and conference proceedings)

7） A medical record is recorded on the selection of a therapeutic drug (the drug proposed by the patient) through consultation between the patient and the principal investigator or co-investigator.

8） Regulatory approval for the therapeutic agent(s) has not been obtained for the relevant disease (the therapeutic agents are off-label drugs for the patient)

9） The therapeutic agent is not the subject of a sponsor-initiated clinical trial or an investigator-initiated clinical trial or for advanced medical treatments being performed at study centers in Japan. However, if there are justifications such as geographical reasons why it is difficult for the patient to participate in the abovementioned clinical trials, that patient may participate in the present study. In such a case, state the reason in the electronic data capture (EDC) at the time of patient enrollment.

※ Follow the “C-CAT Clinical Trial Quick Reference Table” provided to Cancer Genome-Based Healthcare Core Base Hospitals” and reference procedures determined separately to check for the existence of clinical trials or advanced medical treatments that use the relevant medicine.

10） Patients will consent to all of the following:

・ Registration of the patient information in the Cancer Genome Information Management Center (C-CAT) and the use thereof for this research

・ Providing data collected for this study to the pharmaceutical companies that provided the drug without charge

11） Patients must not have carcinomatous meningitis or symptomatic brain metastases

12）Patients must not have pericardial fluid, pleural fluid, or ascites fluid that requires regular puncture
13）Patients must have a Performance Status (ECOG) of 0 or 1. (If the patient is younger than 15 years of age and evaluation by ECOG is difficult, the performance status scale/score conversion table (Appendix) should be used to confirm that the patient’s performance status is equivalent to 0 or 1 on the ECOG scale.)
14）At the time of enrollment, patients must not have been administered anti-cancer drugs (chemotherapy, molecular-targeted therapy, immunotherapy, endocrine therapy, etc.) nor have undergone surgery with general anesthesia (not including bone resorption modifiers such as bisphosphonates and denosumab for bone metastases)
15）At the time of enrollment, patients must not have received radiation therapy or radiopharmaceuticals (except radiopharmaceuticals for diagnostic purposes)
16）The following points [1]-[6] in the clinical examination conducted within 14 days before enrollment must be satisfied. However, patients must not have been administered granulocyte colony-stimulating factor (G-CSF preparations) or have received a transfusion within 14 days before the blood draw

① Neutrophil count ≥ 1000/mm3
② Platelet count ≥ 10×104/mm3
③ AST（GOT）≤ 100 U/L（≤ 150 U/L in cases with liver metastases）
④ ALT（GPT）≤ 100 U/L（≤ 150 U/L in cases with liver metastases）
⑤ Total bilirubin ≤ 2.0 mg/dL
⑥ Serum creatinine ≤ 1.5 mg/dL
However, even if the serum creatinine is ≥ 1.5 mg/dL, the patient will still be eligible if the eGFR ≥ 60 mL/min/1.73 m2.

Use the criteria below for children (under 15 years of age)
a) AST≦3x the upper limit of the clinical reference range*1
b) ALT≦3x the upper limit of the clinical reference range*1
c) Total bilirubin≦1.5x the upper limit of the clinical reference range*1
d) Serum creatinine≦1.5x the upper limit of the clinical reference range*1

*1： Use the “Pediatric Clinical Reference Ranges” (Appendix)

17）Patients must be eligible for Japanese public medical insurance and the patients themselves or, if the patient is under 18 years of age, the patient’s legal representative (in principle, the legal guardian*) must have given written consent for study participation. However, even if the patient understands the content of the explanation and wishes to consent but it is difficult for him/her to sign due to neurological symptoms, etc., if the patient himself/herself so wishes, it is possible to have a substitute sign the confirmation of the patient's consent (a “substitute signatory” refers to a person designated by the subject himself/herself, such as the subject's spouse, adult child, parents, adult siblings, adult grandchildren, grandparents, relatives living together, or those who are considered to be close relatives).

* This refers to a person designated by the subject himself/herself, as a rule the parents, or guardian in the case of difficulties, the subject’s spouse, adult children, adult siblings, adult grandchildren, grandparents, relatives living together, or those who are considered to be close relatives).

4.2. Exclusion criteria

1） Patients whose participation in the research is considered to be difficult because of a psychiatric disorder or psychiatric symptoms that impair daily life.
2） Patients with active infections that require systemic treatment.
3） Patients with a complicating active digestive tract ulcer.

4） Patients with complicating interstitial lung disease or pulmonary fibrosis diagnosed by imaging findings or clinical findings, or with a history thereof.
5） Patients with imaging tests showing an interstitial shadow, or with inflammatory changes in the lungs such as active radiation pneumonitis or infectious pneumonia.
6） Patients who test positive for any of HIV antibodies, HTLV-1 antibodies, HBs antigens, HCV antibodies (however, even if positive for HCV antibodies, patients with no HCV-RNA detected will not be excluded). All of these require confirmation via blood test.
7） Patients who are negative for HBs antigens, positive for HBs antibodies or HBc antibodies, and positive for HBV-DNA quantification (the patient may be enrolled if the result is below the detection sensitivity). However, if the history of hepatitis B vaccination can be confirmed in writing, HBV-DNA quantification is not required.
8） Female patients who are pregnant, breastfeeding (cannot be enrolled even if breastfeeding is discontinued), or can become pregnant.
9） Any patient deemed ineligible by the principal investigators or co-investigators (including when the attending physician deems the patient to be unsuitable due to individual drug characteristics).

5. Enrollment

5.1. New participation procedure (in the case of the first patient in each study center)

1） Procedures under the Clinical Research Act

The Study Representative Physician/Study Secretariat will apply to the Certified Clinical Research Review Board for a change in the research plan (addition of a study center). If approval is obtained, the application is submitted for deliberation at the Patient-Proposed Healthcare Service Evaluation Committee. Based on the results of the deliberation, the addition will be published in the jRCT after obtaining the approval of the director of the relevant study site.

2） Applications based on the patient-proposed healthcare service system

Study centers wishing to participate in this research should ensure that they meet the requirements of “15.1.1. Requirements for study centers,” and fill out and send in the Patient-Proposed Healthcare Service Notification Form.

The Coordination Office will confirm the content of the notification form, and after obtaining approval from the Study Representative Physician and the Study Secretariat, the Central Hospital Patient-Proposed Healthcare Service Committee of the National Cancer Center Hospital, which is a clinical research core hospital under medical law, will be asked to examine whether or not the medical institution should participate in this research. If approval is obtained from the Central Hospital Patient-Proposed Healthcare Service Committee, the Coordination Office will notify the local Public Welfare (Branch) Office within seven days of the approval. Thereafter, the Minister of Health, Labour and Welfare will send a notification on the decision to add the relevant medical institution to the study centers. The Coordinating Office will confirm that the Minister of Health, Labour and Welfare has issued notification of the decision and will contact the principal investigator of the study center.

3） Procedures for conducting this study

Prior to the start of patient enrollment, it is necessary to conclude a written contract that defines the roles of both parties in this research between the National Cancer Center Hospital, which is the main research institute, and the study center

Each study center will start enrollment of the first patient upon completion of the three procedures above (see “5.2 Patient Enrollment”).

5.2. Patient enrollment (in the case of the second and later patients in each study center)

When the patient is the second or later patient in each study center, patient enrollment will be performed according to the procedure shown below

5.2.1. Enrollment procedure

The principal investigator or co-investigator will confirm that the subject patient meets all the eligibility criteria and does not meet any of the exclusion criteria, and will then enroll the patient using electronic data capture (EDC).

The URL and procedures will be notified separately in the “EDC Input Manual.”

Patient enrollment number 1901- Y/N - ○○○ - ･･･

[Trial number]-[presence of target lesion]-[Medication name]-[Serial number]

(Contact person for questions relating to patient selection criteria)

Study secretariat
Tatsunori SHIMOI
Department of Medical Oncology, National Cancer Center Hospital
5-1-1, Tsukiji, Chuo-ku, Tokyo, JAPAN 104-0045
Tel: 03-3542-2511 (direct line 2331)
Fax: 03-3542-3567

Email：tshimoi@ncc.go.jp

Kuniko SUNAMI
Department of Pathology and Clinical Laboratories, National Cancer Center Hospital
5-1-1, Tsukiji, Chuo-ku, Tokyo, JAPAN 104-0045
Tel: 03-3542-2511 (direct line 7751)
Fax: 03-3542-3567
Email: ksunami@ncc.go.jp

(Contact person for questions relating to patient enrollment and EDC)

Data Management Supervisor
Data Management Office, Data Management Section, National Cancer Center Hospital
5-1-1, Tsukiji, Chuo-ku, Tokyo, JAPAN 104-0045
Tel: 03-3542-2511 (direct line 2615)
Fax: 03-3542-3567
Email: NCCH1901_office@ml.rel.ncc.go.jp
Weekdays 9am–5pm (excluding Saturday, Sunday, holidays, and New Year holiday)

5.2.2. Cautions during enrollment

1） Eligibility confirmation is performed on the enrollment screen.
2） Enroll the patient after confirming that he/she is not the subject of a sponsor-initiated clinical trial or an investigator-initiated clinical trial or for advanced medical treatments at a study center in Japan. However, the patient may participate in the present study if there is a justifiable reason that it is difficult to participate in these other clinical trials, such as geographical reasons. In such a case, state the reason in the EDC.
3） When the input data are insufficient, the enrollment will not be accepted until all requirements are satisfied.
4） After eligibility has been confirmed in EDC, an enrollment number will be issued and enrollment will be completed.
5） Patients will not be disenrolled (deleted from the database) except in the case of withdrawal of consent, including refusal for the data to be used for research.
Contact the Study Secretariat promptly when an incorrect enrollment is found

5.3. Procedure for the completing enrollment for each drug

If it is anticipated that the planned number of enrollments will be achieved in each drug cohort, the Study Representative/Study Secretariat will inform the Principal Investigator of the achievement of the number of enrollments and issue precautions for future patient enrollment.

6. Treatment planning and treatment change criteria

6.1. Protocol treatment

Treatments and treatment changes should be performed as described in this chapter unless patient safety is threatened. Promptly start the protocol treatment as soon as the drug is received. Use the drug selected according to 2.2.4 as the protocol treatment. Start the treatment after an appropriate interval from the previous treatment according to the package insert.

6.1.1. Chemotherapy

1） Treatment regimen

The treatment regimen (dosage and administration) for each medicinal product is as described in the latest edition of the package insert.

Combination with cytotoxic chemotherapeutic drugs is not permitted in this study even if combination with other drugs is permissible according to the package insert. When single agents are used in combination with other medicines, follow the dosage and administration from the package insert. The drugs that can be used in combination in this study are defined according to “19. Appendix: List of medicines.”

For each drug, changes such as delays (prolongation of dosing interval, delaying administration more than what is prescribed), pauses (temporary suspension or withdrawal that can resume if certain conditions are met), and supportive care and concomitant use contraindications, etc. should comply with the package insert.

The package inserts can be obtained from the search page of the Japan Pharmaceuticals and Medical Devices Agency.

Pharmaceutical product information search page: http://www.pmda.go.jp/PmdaSearch/iyakuSearch/

2） Calculating the administered dose

For pharmaceuticals for which the dose is defined by body weight or body surface area, the initial dose is calculated using body weight or body surface area recorded at enrollment.

As a rule, the actual dose of each medicine is rounded off to two significant figures.* The amount administered should not exceed the maximum dose specified in the package insert.

For pediatric patients less than 15 years of age, body surface area should be calculated using the Mosteller formula.

Body surface area ［㎡］=√(height［cm］×body weight ［kg］/3,600)

* Two significant figures: calculate with reference to the following example

When the amount is calculated in terms of body surface area or weight, proceed as follows:

Example 1) 134 mg = 1.34×10^{2} → round down the 4 → 130 mg is the actual dose

Example 2) 0.85 mg = 8.50×10^{-1} → round down the 0 → 0.85 mg is the actual dose

6.1.2. Consultations on treatment

Contact the Study Secretariat with any questions about the treatment.

(Contact person for questions relating to treatment)

Study secretariat
Tatsunori SHIMOI
Department of Medical Oncology, National Cancer Center Hospital
5-1-1, Tsukiji, Chuo-ku, Tokyo, JAPAN 104-0045
Tel: 03-3542-2511 (direct line 2331)
Fax: 03-3542-3567
E-mail: tshimoi@ncc.go.jp

6.2. Criteria for discontinuing/terminating the protocol treatment

6.2.1. Definition of terminating the protocol treatment

Since the protocol treatment is to be continued until the protocol treatment discontinuation criteria are met, there is no definition for terminating the protocol treatment. If the upper limit of the total administration period for each drug is stated in the package insert, observe this limit.

6.2.2. Criteria for discontinuing the protocol treatment

The protocol treatment should be discontinued if any of the following is true.

1） When it is determined that the protocol treatment is ineffective according to any of the following
- ① If a marked increase in the lesion was observed after starting treatment, and it was determined necessary to change treatment.
  If it is determined that continuing the protocol treatment is clinically appropriate even if it is determined as “progressive disease” (PD) by the response evaluation from imaging, the protocol treatment is continued and not discontinued.
- ② If a deterioration of clinical symptoms determined to be due to disease progression is observed

2） If the protocol treatment cannot be continued due to an adverse event
- ① When a Grade 4 adverse event is observed (except for the following events)
  - ･ Hematologic toxicity: anemia, hypocellular marrow, lymphocyte count decrease, neutrophil count decrease, leukocytopenia, platelet count decrease, or CD4 lymphocytopenia according to the “Japanese translation JCOG version of the NCI Common Terminology Criteria for Adverse Events v 5.0 (CTCAE v5.0)" (hereinafter, CTCAE v5.0-JCOG) (13)
  - ･ Non-hematologic toxicity: Alkaline phosphatase increase, GGT increase, high cholesterol, hypertriglyceridemia, hyperuricemia, and transient electrolyte abnormality according to the CTCAE v5.0-JCOG

  For an adverse event which was not related to the protocol treatment, if the event was improved or recovered and the restarting the protocol treatment was considered to be profit for the patietns, the Principal Investigator or the Co-investigator can discuss the restarting with the Study Representative/Study Secretariat.
- ② If it was determined that the Principal Investigator or Co-investigator would need to discontinue the protocol treatment due to an adverse event that does not fall under [1] above

3） If the patient requests that the protocol treatment be discontinued because an association with the adverse event cannot be ruled out
- ･ Use this classification if the association with the adverse event cannot be denied

4） If the patient requests that the protocol treatment be discontinued because of the reason(s) that an association with the adverse event cannot be ruled out
- ① In case of refusal by the patient before starting protocol treatment after enrollment
- ② If the association with adverse events cannot be ruled out because of the relocation of the person or family member during protocol treatment

5） Death during the protocol treatment
- ･ Death before deciding to discontinue protocol treatment for other reasons

6） Additional reasons include exacerbation before the start of treatment after enrollment (could not start the protocol treatment due to rapid exacerbation), a protocol violation was found, an ineligibility was discovered due to a change in the pathologic diagnosis after enrollment and treatment was changed, etc., or it is determined that continuation of the protocol treatment is difficult due to social reasons or safety management problems

The protocol discontinuation date is the death date in case of 5), or the day when the Principal Investigator or the Co-investigator determines the subject to be ineligible after enrollment; otherwise it is the day when the Principal Investigator or the Co-investigator determines that the protocol treatment should be discontinued.

6.3. Post-treatment

In the present study, the post-treatment after discontinuing the protocol treatment is not particularly defined, but the following post-treatment can be assumed.

1. Participation in patient-proposed healthcare services for medications for other different genetic mutations obtained from the initially submitted specimens
2. Participation in patient-proposed healthcare services for the same type of medication for the same genetic abnormalities
3. Conducting some form of chemotherapy for practical diagnosis and treatment
4. Participation in other clinical trials and studies
5. Follow-up observation/best supportive care, etc.

7. Expected adverse events

7.1. Adverse reactions expected with the drug

The adverse reactions expected for each drug in this study are different. For this reason, refer to the latest edition of the package insert for the adverse reactions expected for the drugs used in protocol treatment.

7.2. Evaluating adverse events/adverse reactions

Use the "Japanese translation JCOG version of the NCI Common Terminology Criteria for Adverse Events v 5.0 (CTCAE v5.0)" (hereinafter, CTCAE v5.0-JCOG) to evaluate the adverse events/adverse reactions in this study. Regarding the parameters for which grades are defined as the laboratory standard value in the CTCAE v5.0-JCOG, the "JCOG Common Standard Reference Range" is used instead of the in-house standard value for each medical institution. Refer to the JCOG website (http://www.jcog.jp/doctor/tool/kijun.html) for details on the "JCOG Common Reference Range." For children under 15 years of age, abnormalities in laboratory test values listed in the CTCAE Grade Definition Table for Pediatric Clinical Reference Ranges (Appendix) should be graded by using the Appendix, and the event should be recorded in the eCRF Adverse Event Form.

7.2.1. Grading adverse events

When grading adverse events, each event is given the grade closest to the defined grades 0–4. In addition, if a specific treatment is described for a grade, grade it based on its clinical necessity. For example, when the patient's pleural fluid is increasing and the patient refuses oxygen inhalation and chest drainage despite these being indicated in this situation. In these cases, the grade should be assigned based on the medical determination of what should be done, not what was actually done.

7.2.2. Causal relationship between adverse events and treatment

When determining cause and effect relationships between adverse events and treatment, these are classified into five categories: "definite, probable, possible, unlikely, and not related." A determination of any one of "definite, probable, possible" is defined as "causality present," and a determination of "unlikely, not related" is defined as "causality absent" (see: Table 7.2.2).

If, based on the adverse event's grade, the adverse event is classified as a serious adverse event for which there is an obligation to provide an emergency report as defined in "10.1. Serious adverse events and events subject to emergency reports", it should be reported to the Study Representative Physician in accordance with "10.2. Requirements and procedures for reporting to the Study Representative Physician."

Table 7.2.2 Determination criteria for causal relationships between adverse events and treatment

	Determination	Rationale for determination
Cause and effect relation-ship present	Definite	The AE is clearly related to the intervention<br>It is clear that the adverse event was caused/made more severe by the protocol treatment, and it is determined that there is little possibility of being due to exacerbation of the underlying disease or by other factors (comorbidities, other medications/treatments, coincidence)
	Probable	The AE is likely related to the intervention<br>The adverse event is unlikely to have occurred/been made more severe as a result of exacerbation of the underlying disease or by other factors (comorbidities, other medications/treatments, coincidence), and the possibility of being due to the protocol treatment is determined to be high
	Possible	The AE may be related to the intervention<br>It is more plausible to consider that the adverse event is caused/made more severe by protocol treatment, and while it might be due to exacerbation of the underlying disease or by other factors (comorbidities, other medications/treatments, coincidence), that possibility is determined to be low
Cause and effect relation-	Unlikely	The AE is doubtfully related to the intervention<br>Even though the adverse event could be considered to be caused/made more severe by protocol treatment, it is determined more likely to be due to exacerbation of the underlying disease or other factors (comorbidities, other

ship absent		medications/treatments, coincidence)
	Not related	The AE is clearly NOT related to the intervention<br>The adverse event is apparently caused/made more severe by exacerbation of the underlying disease or other factors (comorbidities, other medications/treatments, coincidence), and it is determined that there is no possibility of being due to the protocol treatment

8. Evaluation parameters, clinical testing, and evaluation scheduling

8.1. Pre-enrollment evaluation parameters

8.2. Pre-enrollment evaluation parameters (any time before enrollment)

The results and findings of tests conducted prior to obtaining consent will also be available as data.

1） Sex
2） Age (Date of birth)
3） Height, body weight (carried out within 14 days before enrolment in children under 15 years of age)
4） Medical treatment history
・Operation history (any surgery at the primary site, any surgery for metastasis/recurrent disease)
・Radiotherapy history (any perioperative treatment, any metastasis/recurrent disease)
・Pharmaceutical treatment history (number of regimens, any perioperative treatments, any metastasis/recurrent disease)
5） History of cancer/multiple cancers (any cases, and related cancer types)
6） Family medical history
7） Cancer type
・Histopathology exams*: date of specimen collection, histologic classification (according to the Rare Care classification)
* The timing of implementation does not matter as long as it is occurs before enrollment. However, if any histopathology findings come from another facility, a pathology specimen should be obtained and a pathology diagnosis be redone at the study center.
・Classified as first occurrence/recurrence
・Any metastasis present/metastatic lesion
8） Gene panel test
・Test name/specimen collection date/examination date
・Genetic abnormality (related to the relevant drug selection)
Gene name
Type of abnormality: [1] gene mutation, [2] fusion gene, [3] gene deficiency, [4] gene amplification
[1] CDS change/amino acid change/allele frequency/differentiation of somatic cells/germ cells
[2], [3] Ratio of copies
[4] Details (if the partner gene or fusion point is known)
・TMB-high/MSI-high
・Misc. (Total number of exon mutations (tumor mutation burden)/total exon mutation rate/total non-exon/number of mutations/non-exon total mutation rate/total number of mutations in all regions/total mutation rate in all regions)
9） C-CAT number

8.2.1. Tests performed within 28 days before enrollment

The results and findings of tests conducted prior to obtaining consent can also be used as data.

1) Imaging examination (contrast-enhanced CT or MRI: select the modality according to the cancer type)

8.2.2. Tests performed within 14 days before enrollment

The following measurements are made to confirm safety.

1) ECOG Performance Status（PS）(If the patient is younger than 15 years of age and evaluation by ECOG is difficult, the performance status scale/score conversion table (Appendix) should be

used to confirm that the patient's performance status is equivalent to 0 or 1 on the ECOG scale.)

2) Hematology test: leukocyte count, differential leukocyte count (neutrophils, lymphocytes), hemoglobin, and platelet count
3) Blood biochemistry test: albumin, total bilirubin, AST, ALT, creatinine, sodium, potassium, LDH, calcium (albumin correction*)

   * When serum albumin level is ≤ 4.0 g/dL, use the following correction(12)

   Corrected calcium (mg/dL) = serum calcium level (mg/dL) + (4-serum albumin level)

8.2.3. Tests performed within 7 days before starting treatment

The following measurements are made to confirm safety.

1) ECOG Performance Status（PS）(If the patient is younger than 15 years of age and evaluation by ECOG is difficult, the performance status scale/score conversion table (Appendix) should be used to confirm that the patient's performance status is equivalent to 0 or 1 on the ECOG scale.)
2) Hematology test: leukocyte count, differential leukocyte count (neutrophils, lymphocytes), hemoglobin, and platelet count
3) Blood biochemistry test: albumin, total bilirubin, AST, ALT, creatinine, sodium, potassium, LDH, calcium (albumin correction*)

   * When serum albumin level is ≤ 4.0 g/dL, use the following correction(12)

   Corrected calcium (mg/dL) = serum calcium level (mg/dL) + (4-serum albumin level)
4) If the last imaging examination (contrast-enhanced CT or MRI: select the modality according to the cancer type) are performed more than 28 days before, it is desirable to conduct the assessment again by the start of the treatment (the assessment at the start of treatment is available).

8.3. Tests and evaluations during the protocol treatment period

8.3.1. Safety endpoints

In this study, protocol treatments are carried out with various medicines, so there is no uniform safety endpoint, but the patient's safety is evaluated with due consideration by the doctor in charge according to the description in the respective drug package insert, and tests are conducted as appropriate. The evaluations are based on "7.2 Evaluating adverse events/adverse reactions," and when they correspond to "10.1 Serious adverse events and events subject to emergency reports" they are reported to the Study Representative Physician according to "10.2 Requirements and procedures for reporting to the Study Representative Physician." Otherwise, enter into the EDC the worst grade ≥ 2 adverse events that have occurred from the start of treatment to 30 days after treatment discontinuation or the date of subsequent treatment initiation, wchichever comes first. And the assessment of them to 30 days after treatment discontinuation or the date of initiation of subsequent treatment, wchichever comes first. If adverse events are considered to be related to protocol treatment or they are severe regardless of relation to protocol treatment, follow-up will be continued as long as possible until the adverse event is considered resolved, resolved, or stable.

※Instruction of safety information shall be in accordance with the separately specified procedures.

8.3.2. Efficacy endpoints

Perform the imaging evaluation 16±1 weeks after the start of treatment for evaluating the primary endpoint. Moreover, whether or not the imaging evaluation is performed by the 16th week, the frequency of when the imaging evaluation is performed is variable. When a complete response (CR) or partial response (PR) in the overall efficacy is seen for the first time, an additional imaging examination will be conducted in the 4th week thereafter for confirmation. The evaluation is performed according to "11.1. Efficacy evaluation."

During the protocol treatment period, it is desirable to conduct an assessment by contrast CT or

contrast MRI using the same modality and imaging conditions used in the pre-enrollment assessment.

8.4. Tests and endpoints when the protocol treatment is completed

Record the following information if treatment is discontinued.

- Treatment discontinuation date (the date when it was determined that the protocol treatment was to be discontinued), and the reason for discontinuation
- Adverse events between the start of treatment and within 30 days after treatment was completed
- Imaging exam for tumor evaluation

8.5. Follow-up

From the time of the first patient enrollment in this study to when the study is completed, follow-ups are performed every 6 months for a period of 3 years to confirm patient survival (in case of death, confirm the date and cause of death), and to confirm any exacerbation (presence and date of any exacerbation, or the date no exacerbation was confirmed). Confirmations of outcome can be done by phone or letter.

8.6. Study calendar

Parameter	Screening	Protocol treatment			Protocol after treatment is completed
		At start of treatment	During treatment	At completion of treatment	
Consent	◎				
Patient background	◎*1				
Treatment details (Name of drug)		◎			
Date treatment started		◎			
ECOG-PS	◎*2	◎			
Date treatment discontinued				◎	
Date exacerbation confirmed					◎
Gene panel test	◎*1				
Hematological tests	◎*2	◎	○	○	
Blood biochem. tests	◎*2	◎	○	○	
Tumor response evaluation （CT/MRI）*3	◎*4	○*5	◎*6	◎	
Adverse Events				◎*7	
Follow-up					◎

◎: Required, ○: Optional
*1: Any time before enrollment
*2: Within 14 days before enrollment
*3: Select modality according to cancer type
*4: Within 28 days before enrollment
*5: Within 28 days before the start of treatment (including the date treatment started)
*6: Required 16±1 weeks after the start of treatment (otherwise as appropriate)
*7: From the start of protocol treatment to 30 days after protocol treatment discontinuation or the date of subsequent treatment initiation, wchichever comes first. (assess the events to 30 days after treatment discontinuation or the date of initiation of subsequent treatment, wchichever comes first). If adverse events are considered to be related to protocol treatment or they are severe regardless of relation to protocol treatment, follow-up will be continued as long as possible until the adverse event is considered

resolved, resolved, or stable.

*8: Within 14 days after treatment discontinuation date

9. Data acquisition

9.1. Case Report Form（CRF）

9.1.1. Data reports (including changes and corrections)

Researchers can rapidly enter clinical data into an eCRF via the Electronic Data Capture (EDC) system.

Inputs from collaborators can be transcribed from the medical record. The procedures for eCRF input and correction are according to the “EDC Input Manual.”

9.1.2. Types of eCRF

System name	CLASSIC RAVE
System development	Medidata Solutions Co., Ltd.

9.2. CRF storage

Data management in this study will be performed by the data management coordinator (Data Management Office, Data Management Section, Clinical Research Support Office, National Cancer Research Center Hospital).

Data entered into the eCRF is stored on the EDC server under the responsibility of the Data Management Officer.

Diagnosis and treatment records and various medical records (electronic medical record data), “materials related to the relevant information (case report forms, records prepared by the study subjects, records of corrections, etc.),” and “other materials (notifications of results such as research plan revisions made by the head of the study institution, implementation status reports, adverse event reports, research plans, and informed consent forms, etc.) are stored in accordance with the procedures established by each study center.

The original materials in these documents are as follows.

1) Diagnosis and treatment records
2) Imaging exam films (including electronic data)
3) Consent forms
4) The following data described in CRFs
   ① Eligibility of enrolled subjects
   ② Descriptions related to adverse events (seriousness, grade, causal relationships with medical devices, and outcomes)
   ③ Comments

The direct access to these original materials is granted as follows.

The Principal Investigator is responsible for monitoring related to clinical research, audits, and the Certified Clinical Research Review Board, and during regulatory investigations, provides direct access to all clinical research-related records, including the original data.

10. Disease, etc. (adverse event) reports

According to the provisions of this chapter based on the "Clinical Research Act" (Law No. 16 of 2017), the "Ordinance for Enforcement of the Clinical Research Act" (Ministry of Health, Labour and Welfare Ordinance No. 17 of 2018), and related notifications, "Serious adverse events ("diseases, etc." under the Clinical Research Act) that occur are to be reported to the Certified Clinical Research Review Board and the Minister of Health, Labour and Welfare.

Since this research is based on a patient-proposed healthcare services system, based on "the considerations and handling of proposals, etc. in the implementation of patient-proposed healthcare services as stipulated in the "Health Insurance Act and the Law for Assuring Healthcare for the Elderly" (in force as of March 26, 2018, based on Health Policy Bureau (HPB) notification no. 0326.3, Pharmaceutical Safety Bureau (PSB) notification no. 0326.2, and Health Insurance Bureau (HIB) notification no. 0326.2) and the detailed regulations thereof, when serious adverse events occur, these must be reported to the Local (Branch) Bureaus of Health and the Medical Economics Division, Health Insurance Bureau, of the Ministry of Health, Labour and Welfare.

10.1. Serious adverse events and events subject to emergency reports

Serious adverse events are those that fall under any of the following. Death

1） Diseases, etc. that can lead to death
2） Diseases, etc. that require hospitalization or prolongation of hospitalization for treatment
3） Disability
4） Diseases, etc. that can lead to disability
5） Diseases, etc. that are serious according to 1) to 5)
6） Diseases or abnormalities that can lead to congenital diseases in later generations

Of these events, those that fall into any of the following categories are defined as serious adverse events for which there is an obligation to provide an emergency report.**1) Death**

① [1] Any death that occurs after enrollment and before starting the protocol treatment
② Any death during the protocol treatment or within 30 days of the last treatment (whether or not there is a causal relationship with the protocol treatment)
③ Death ≥ 31 days after the last treatment if a causal relationship with the protocol treatment cannot be ruled out (definite, probable, possible)

2) Diseases, etc. that can lead to death

① Grade 4 adverse event occurring during the protocol treatment or within 30 days from completing the treatment
② Grade 4 adverse event occurring ≥ 31 days after the last treatment if a causal relationship with the protocol treatment cannot be ruled out (definite, probable, possible)

3) Unexpected[*2] diseases, etc. that require hospitalization or prolongation of hospitalization for treatment[*1]

① Grade 3, 2, or 1 adverse event occurring during the protocol treatment or within 30 days from completing the treatment and the treatment for the adverse event requires a hospitalization or a prolongation[*] of a hospitalization of ≥ 24 hours
② Grade 3, 2, or 1 adverse event occurring ≥ 31 days after the last treatment and the treatment for the adverse event requires a hospitalization or a prolongation[*] of a hospitalization of ≥ 24 hours if a causal relationship with the protocol treatment cannot be ruled out (definite, probable, possible)

[*1] The phrase "hospitalization or prolongation of hospitalization" indicates only when a hospitalization/prolongation of a hospitalization of ≥ 24 hours is medically necessary, and an emergency report need not be submitted if outside this reporting scope in the following ways.

- Tumor-associated symptoms due to worsening of primary disease

- Hospitalization/prolongation of hospitalization for follow-up of adverse events that have disappeared or resolved
- Hospitalization/prolongation of hospitalization for the purpose of reducing the burden on a patient who has come from a remote location

Other hospitalization/prolongation of hospitalization with no medical necessity

*2 "Unexpected" means events not listed in "7. Expected adverse events" or in the latest package insert of the relevant medication.

Unexpected 4) Disability or 5) disease, etc. that can lead to disability

Permanent or significant disability or dysfunction (except myelodysplastic syndrome (MDS), secondary cancers, etc.), or at risk thereof

6) Unexpected diseases, etc. that are serious according to 1) to 5)

7) Unexpected diseases or abnormalities that can lead to congenital diseases in later generations

The following events are not subject to emergency reporting. However, any that are judged to correspond to the serious adverse events in 1) to 7) above should be reported according to the procedure of 10.2.

・Hematotoxicity: "Anemia," "Bone marrow hypocellular," "Decreased lymphocyte count," "Decreased neutrophil count," "Decreased leukocyte count," "Decreased platelet count," and "Decreased CD4 lymphocyte count" in the "Japanese translation of NCI-Common Terminology Criteria for Adverse Events v5.0 (CTCAE v5.0)" (CTCAE v5.0-JCOG) (11)

・ Non-hematological toxicity: "alkaline phosphatase increase," "GGT increase," "high cholesterol," "hypertriglyceridemia," and transient electrolyte abnormalities in the CTCAE v5.0-JCOG

10.2. Requirements and procedures for reporting to the Study Representative Physician

10.2.1. Urgent reports

In the case of the occurrence of a serious adverse event for which there is an obligation to submit an emergency report as stipulated in 10.1., the Co-investigator will promptly report this to the Principal Investigator. If the Principal Investigator cannot be reached, the Co-investigator must assume the responsibility of the Principal Investigator. The Principal Investigator will make reports according to the following procedures. Care must be taken not to include the patient's name or medical record number, etc. when submitting a report.

1） **Death or diseases, etc. that can lead to death as provided in 10.1. 1), 2)**

Primary report:

The Co-investigator will promptly report the occurrence of a serious adverse event to the Principal Investigator. Having received the report, the Principal Investigator will as soon as possible enter the prescribed items in the "NCCH1901 adverse event report (for facility use)" and in the "Report on a disease, etc." to be delivered to the Certified Clinical Research Review Board as defined under the Clinical Research Act Enforcement Regulations within 72 hours after learning of the occurrence of the adverse event, and will contact the Study Representative Physician/Study Secretariat by email.

Secondary report:

Within 7 days after learning of the occurrence of the adverse event, the Principal Investigator will append detailed information on the adverse event to the "NCCH1901 adverse event report (for facility use)" and to the "Report on a disease, etc." and "Detailed description form" to be delivered to the Certified Clinical Research Review Board as defined under the Clinical Research Act Enforcement Regulations, and will submit the same to the Study Representative Physician/Study Secretariat by email. If necessary, attach copies of examination data, images, autopsy results reports, etc. If a report to the Minister of Health, Labour and Welfare is required under the Clinical Research Act as per 10.3.2. 2), this should be added to "Attached Form 2-1" as well.

2） **10.1 Unexpected events involving 3) diseases, etc. that require hospitalization or prolongation of hospitalization for treatment or for other adverse events determined to be medically important conditions ((10.1. 4)-7)**

The Co-investigator will promptly report the occurrence of a serious adverse event to the Principal

Investigator. Within 10 days after learning of the occurrence of the adverse event, the Principal Investigator will append detailed information on the adverse event to the "NCCH1901 Adverse event report (for facility use)" and to the "Report on a disease, etc." and "Detailed description form" to be delivered to the Certified Clinical Research Review Board as defined under the Clinical Research Act Enforcement Regulations, and will submit the same to the Study Representative Physician/Study Secretariat by email. If necessary, attach copies of examination data, images, autopsy results reports, etc.

3) **Supplemental report**

If new information is obtained after making the above report, the Principal Investigator will report as needed and append this information to the "Report on a disease, etc." and "Detailed description form" addressed to the Certified Clinical Research Review Board as defined under the Clinical Research Act Enforcement Regulations.

Table 10.2.1 Adverse events subject to urgent reporting and timelines for reporting to the Study Representative Physician/Study Secretariat

Causal relationship	Grade 1-3, hospitalization Other medically severe conditions*1		Grade 4		Death	
	Expected	Unexpected*2	Expected	Unexpected	Expected	Unexpected
Yes	Not reported	Initial: within 10 days<br>Supplemental: as needed	Primary report: within 72 h<br>Secondary report: Within 7 days<br>Supplemental report: as needed			
No	<Only when occurring during the protocol treatment or within 30 days from completing the treatment>					
	Not reported	Initial: within 10 days<br>Supplemental: as needed	Primary report: within 72 h<br>Secondary report: Within 7 days<br>Supplemental report: as needed			

*1 4) Disability; 5) Disease, etc. that can lead to disability; 6) Diseases, etc. that are severe according to 1) to 5); and 7) Congenital diseases or abnormalities in later generations as stipulated in 10.1.

*2 "Unexpected" indicates events not described under "7. Expected adverse events" and events not described in the latest package insert for the relevant medication.

10.2.2. Report to the Study Center Director

If the occurrence of an adverse event is the subject of an emergency report, and after being reported to the Study Representative Physician it is reported to the Certified Clinical Research Review Board as having been determined to have a causal relationship (see Table 10.3.2.-1), the Principal Investigator will report this to the corresponding Study Center Director as per the regulations of the corresponding medical institution.

10.3. Responsibilities of the Study Representative Physician/Study Secretariat

10.3.1. Determining if there is a need to suspend enrollment and issue an urgent notification to the facility

The Study Representative Physician/Study Secretariat who received a report from the Principal Investigator will determine the urgency, importance, degree of impact, etc. of the report content, and will temporarily suspend enrollment and/or will take measures such as issuing emergency communication to publicize the information to participating facilities as necessary.

10.3.2. Reporting to the Certified Clinical Research Review Board, the Minister of Health, Labour and Welfare, the Local (Branch) Health and Welfare Bureau, and the Medical Economics Division,

Health Insurance Bureau, of the Ministry of Health, Labour and Welfare.

1）**Reporting to the Certified Clinical Research Review Board under the Clinical Research Act**

The Study Representative Physician/Study Secretariat will comprehensively examine the validity of the causality of the adverse event in the urgent report from the facility based on the package insert and other materials, and whether the event was expected or not, and will prepare Unified Format 8 and report to the Certified Clinical Research Review Board within the following periods after learning of the occurrence of the adverse event.

Reporting timelines

After learning of the occurrence of the adverse event, the Study Representative Physician/Study Secretariat will report to the Certified Clinical Research Review Board within the following periods.

Table 10.3.2.-1) Adverse events subject to urgent reporting and timelines for reporting to the Certified Clinical Research Review Board

Causal relation ship	Grade 1-3, hospitalization Other medically severe conditions		Grade 4		Death	
	Expected	Unexpected	Expected	Unexpected	Expected	Unexpected
Yes	Not reported	Within 15 days	Within 15 days	Within 7 days	Within 15 days	Within 7 days
No	Not reported	Not reported	Not reported	Not reported	Not reported	Not reported

2） **Reporting to the Minister of Health, Labour and Welfare under the Clinical Research Act**

If it is determined that the adverse event reported from the institution is causally related to the protocol treatment and is determined to be unexpected, the Study Representative Physician/Study Secretariat will prepare a report on diseases, etc. to be delivered to the Minister of Health, Labour and Welfare as defined under the Clinical Research Act Enforcement Regulations based on jRCT, and will report to the Minister of Health, Labour and Welfare.

Reporting timelines

After learning of the occurrence of the adverse event, the Study Representative Physician/Study Secretariat will report to the Minister of Health, Labour and Welfare within the following periods.

Table 10.3.2.-2) Adverse events subject to urgent reporting and timelines for reporting to the Minister of Health, Labour and Welfare

Causal relation ship	Grade 1-3, hospitalization Other medically severe conditions		Grade 4		Death	
	Expected	Unexpected	Expected	Unexpected	Expected	Unexpected
Yes	Not reported	Within 15 days	Not applicable	Within 7 days	Not applicable	Within 7 days
No	Not reported	Not reported	Not reported	Not reported	Not reported	Not reported

3） **Reporting on the patient-proposed healthcare services system to the Local (Branch) Health and Welfare Bureau, and the Medical Economics Division, Health Insurance Bureau, of the Ministry of Health, Labour and Welfare.**

When an adverse event in an urgent report from a facility is determined by the Study Representative Physician/Study Secretariat to correspond to any of the following, that adverse event will be reported

using the form specified in the patient-proposed healthcare services system (Form No. 2) to the Local (Branch) Health and Welfare Bureau, and the Medical Economics Division, Health Insurance Bureau, of the Ministry of Health, Labour and Welfare.

Reporting timelines

After learning of the occurrence of the adverse event, the Study Representative Physician/Study Secretariat will report to the Local (Branch) Health and Welfare Bureau, and the Medical Economics Division, Health Insurance Bureau, of the Ministry of Health, Labour and Welfare within the following periods.

Table 10.3.2.-3 Adverse events subject to urgent reporting and timelines for reporting to the Local (Branch) Health and Welfare Bureau, and the Medical Economics Division, Health Insurance Bureau, of the Ministry of Health, Labour and Welfare

Causal relationship	Grade 1-3, hospitalization Other medically severe conditions		Grade 4		Death	
	Expected	Unexpected	Expected	Unexpected	Expected	Unexpected
Yes	Not reported	Within 15 days	Within 7 days	Within 7 days	Within 7 days	Within 7 days
No	Not reported	Within 15 days	Within 7 days	Within 7 days	Within 7 days	Within 7 days

4） **Supplemental report**

After receiving a secondary report or supplemental report from the Principal Investigator, the Study Representative Physician/Study Secretariat will append the additional information from the primary report and the opinion based thereon to a “Report on Diseases, etc.” to be delivered to the Certified Clinical Research Review Board as defined under the Clinical Research Act Enforcement Regulations, and this secondary report or supplemental report will be reported to the Local (Branch) Health and Welfare Bureau, and the Medical Economics Division, Health Insurance Bureau, of the Ministry of Health, Labour and Welfare in the same manner as was done with the primary report.

10.3.3. Notification to the Principal Investigator of each facility

When the Study Representative Physician/Study Secretariat reports to the Certified Clinical Research Review Board, the Principal Investigators of all participating research facilities will be notified in a document (by email) of the details of the review and recommendation. In addition, if there is an urgent need to be informed of the details, the Study Representative Physician/Study Secretariat will notify the Principal Investigators without waiting for the review by the Certified Clinical Research Review Board. Moreover, when a report is made to the Minister of Health, Labour and Welfare, the Study Representative Physician/Study Secretariat will notify the Principal Investigators of all participating research facilities of this subject matter.

If a report has not been made to the Certified Clinical Research Review Board or the Minister of Health, Labour and Welfare, the Principal Investigator of the facility making the report will be notified in a document (by email) of the determination of the Study Representative Physician/Study Secretariat.

10.4. Response to the Principal Investigator of participating facilities (including the relevant facility)

When an adverse event is the subject of a “Report on Diseases, etc.” to the Certified Clinical Research Review Board, the Principal Investigator from the facility participating in this research will report to the Study Center Director according to instructions from the Study Representative Physician/Study Secretariat as per the regulations of the corresponding medical institution.

10.5. Response to the Efficacy and Safety Evaluation Committee

When an adverse event is the subject of an urgent report, the Study Representative Physician/Study Secretariat is to determine whether or not the trial is to be continued, and contacts the Efficacy and Safety Evaluation Committee to request an examination to determine whether or not to continue the trial.

10.6. Reports on pregnancy and overdose

If the patient or partner of the patient is found to be pregnant, or if an overdose of study drug is identified, the principal investigator (or the co-investigator from whom instruction was received) will contact the Coordination Office immediately after obtaining the information. Even if no adverse events are present, the report on pregnancy and overdose should be completed as specified in the “Procedure for Handling

Safety Information" and submitted to the Coordination Office. The Coordination Office will also share information with study drug providers as appropriate.

"Overdose" refers to a case where the dose administered exceeds the dose specified in the package insert by 10%.

10.7. Safety information reports to the company providing the medication

According to the details of the contract, the co-investigator/principal investigator or the Study Representative Physician/Study Secretariat will report adverse event information to the department in charge at the pharmaceutical company that handles the relevant medication (by email, fax, etc.).

11. Efficacy assessments

When any treatment is registered in this study, the tumor response effect assessment will be done according to procedures in "The New Guidelines for the Response Evaluation Criteria In Solid Tumors (RECIST guidelines) revised version 1.1 (JCOG Japanese translation revised version 1.1"(15).

Although the determination interval of overall efficacy is not determined, a comprehensive evaluation is always performed 16±1 weeks after the start of treatment. Furthermore, in this study, we do not collect the details of the efficacy assessments, but collect in the eCRF only the results of the best overall efficacy (11.1.10.) that were confirmed in each treatment.

11.1. Efficacy assessments

11.1.1. Baseline evaluation

In accordance with "8.1. Pre-registration evaluation items," specifics of the neoplastic lesions are identified before enrollment with head, chest, abdomen, and pelvis contrast CT imaging (slice thickness ≤ 5 mm; MRI is also acceptable for head scans), with each lesion classified as a "measurable lesion" or "non-measurable lesion."

The tumor diameter measurement is performed on the cross-sectional image of the CT or MRI scan, and measurements from sagittal or coronary sections from the three-dimensional image reconstruction are not used. The baseline assessment is performed using the most recent imaging exam within 28 days prior to enrollment. If the imaging exam is redone after enrollment and before the treatment starts, the most recent imaging exam results are to be used.

11.1.2. Definition of measurable lesion

Measurable lesions are those that fall under any of the following.

[1] Lesion other than a lymph node lesion (non-lymph node lesion) with a maximum diameter of ≥ 10 mm by CT or MRI

[2] Lymph node lesion with a short diameter ≥ 15 mm by CT (a lymph node lesion with a short diameter of ≥ 10 mm and < 15 mm is a non-target lesion, and a lymph node with short diameter of < 10 mm is not a lesion)

Any lesions other than those described above are non-measurable lesions.

Be aware that the following lesions are non-measurable lesions regardless of the examination method or the size of the lesion.

- Bone lesions (except osteolytic bone metastases with measurable soft tissue components)
- Cystic lesions (except metastatic cystic lesions when there are no other measurable non-cystic lesions)
- Lesions with a history of local treatment such as radiotherapy
- Pial meningeal lesion
- Ascites, pleural effusion, pericardial effusion
- Lymphangiopathy of the skin and lungs
- Abdominal masses and enlargements of abdominal organs that are palpable but not measurable by imaging methods

11.1.3. Selection of target lesion(s) and baseline recording

Among the measurable lesions observed at the time of enrollment, up to 5 target lesions are selected (2 per organ*) in descending order of diameter (long diameter for non-lymph node lesions and short diameter for lymph node lesions). During this process, selections are made taking into consideration that organs having measurable lesions should be included as uniformly as possible and with a focus on reproducible repeated measurements (avoid lesions that are difficult to measure even if the diameter is large).

For the selected target lesions, describe in the chart the lesion site (code), examination method, examination date, long diameter of non-lymph node target lesions, short diameter of lymph node target lesions in order from head to foot, along with the sum of the diameters of all target lesions (hereinafter, “lesion diameter sum”).

* How to count organs

Organize all the left and right organs (lung, kidney, etc.) as a single organ

All lymph nodes are one organ each regardless of site

11.1.4. Baseline recording of non-target lesions

Lesions that were not selected as target lesions, whether or not measurable, are all recorded as non-target lesions, and the lesion site, examination method, and examination date are recorded in the chart. Multiple non-targeted lesions within the same organ can be recorded as one lesion (e.g., multiple enlarged pelvic nodes, multiple liver metastases).

11.1.5. Tumor response evaluation

16±1 weeks after the start of treatment and at the time of treatment discontinuation, and regardless of whether or not treatment will be prolonged, an evaluation of the target lesions and non-target lesions is to be made according to “8.2. Tests and evaluations during the protocol treatment period” in the same manner as during enrollment, and the diameters of the target lesions and any disappearance or exacerbation of non-target lesions are to be recorded in the chart.

Even if the overall efficacy is determined to be "PD," the protocol treatment is not to be discontinued if it is determined that continuation of the protocol treatment is clinically appropriate, and to continue the protocol treatment even after the overall efficacy is determined as PD, the efficacy determination during this period should be performed according to the following.

11.1.6. Criteria for determining efficacy on the target lesions

- **Complete Response（CR）:**
  All non-lymph node target lesions have disappeared and all lymph node target lesions have a small diameter < 10 mm.
  If a lymph node target lesion is selected at baseline, the efficacy on the target lesion can be CR even if the lesion diameter sum is not 0 mm.
- **Partial Response（PR）:**
  Reduction of 30% or more in the target lesion diameter sum compared to baseline
- **Progressive Disease（PD）:**
  An increase of 20% or more in the target lesion diameter sum compared to the smallest lesion diameter sum over the course (when the minimum value occurs at baseline, that will be the smallest lesion diameter sum over the course), and an increase in absolute value of the lesion diameter sum by ≥ 5 mm
- **Stable Disease（SD）:**
  There is no decrease corresponding to PR and no increase corresponding to PD
- **Not all Evaluated（NE）:**
  If an examination cannot be performed for any reason, or if it cannot be judged as CR, PR, PD, or SD

$$\text{Sum reduction ratio} = \frac{(\text{Pre-treatment sum}) - (\text{Sum at evaluation})}{(\text{Pre-treatment sum})} \times 100\%$$

$$\text{Sum increase ratio} = \frac{(\text{Sum at evaluation}) - (\text{Minimum sum})}{(\text{Minimum sum})} \times 100\%$$

① The measured value is to be recorded as long as the diameter of the target lesion can be measured (for example, < 5 mm), but if it is determined that the diameter of the target lesion is "too small to measure" regardless of the CT slice thickness, the determination is that the tumor lesion does not remain when the diameter is 0 mm, and that the tumor does remain when the diameter is 5 mm.

② If the reduction ratio satisfies the condition for PR and at the same time the increase ratio satisfies the condition for PD, PD is assumed.

③ If one lesion separates during treatment, add the diameter of each to the diameter sum.

④ If multiple lesions coalesce during treatment and the border cannot be identified, the diameter of the coalesced lesion is added to the lesion diameter sum. Even if the lesions are in contact with each other, the diameter of each lesion is added to the lesion diameter sum if the lesion boundaries can be identified.

11.1.7. Criteria for determining efficacy on the non-target lesions

- **Complete Response（CR）:**
  All non-lymph node non-target lesions have disappeared and all lymph node non-target lesions have a small diameter < 10 mm.
- **Non-CR/non-PD:**
  When one or more non-target lesions remain (including remaining lymph node non-target lesions with a small diameter of ≥ 10 mm)
- **Progressive Disease（PD）:**
  “Apparent exacerbation” of existing non-target lesions (including recurrences).
  In the case of measurable lesions: even if the efficacy on the target lesion is SD or PR, and an increase in the overall tumor volume is determined as an “apparent exacerbation” based on changes in non-target lesions, a significant exacerbation of non-target lesions should be observed to a sufficient degree to discontinue treatment. When the efficacy on the target lesion is SD or PR, an increase in the tumor volume of the non-target lesions that is far more than the decrease of the tumor volume is regarded as “apparent exacerbation”; otherwise it is Non-CR/non-PD.
  In the case of having only non-measurable lesions: an indication of “apparent exacerbation” is when an increase in non-target lesions is judged to clearly exceed a tumor volume equivalent to a 20% increase in diameter or a 73% increase in tumor volume
- **Not all Evaluated（NE）:**
  If an examination cannot be performed for any reason, or if it cannot be determined as CR, PR, PD, or SD

11.1.8. Appearance of new lesions

If a lesion not present at baseline is observed after the start of treatment, it is considered as the appearance of a “new lesion.”

However, qualifying as a “new lesion” requires that it cannot be a change in the image due to the difference in imaging method from the examination performed during the baseline evaluation or a change in image modality, or a change on the image due to a pathological condition other than a tumor. For example, a cystic lesion formed within a lesion due to necrosis of a liver metastasis is not a new lesion. Lesions newly identified by examination of sites not required at baseline (pre-enrollment evaluation) are considered new lesions. The diameter of the new lesion (maximum diameter of non-lymph node lesions,

short diameter of lymph node lesions) is not added to the lesion diameter sum.

If one lesion disappears and then later reappears, the measurement is continued without making it a "new lesion." However, the efficacy at the time when the lesion reappears differs depending on the condition of other lesions. When a lesion reappears after CR, the overall efficacy is determined to be PD at the time of reappearance. On the other hand, if a lesion that has once disappeared then reappears when the overall efficacy is PR or SD, the diameter of that lesion will be added to the diameter sum of the remaining lesions to calculate the efficacy. That is, in a state in which many lesions remain, even if one lesion apparently reappears after "disappearing," this alone does not make a determination of PD, and it is when the sum of all the lesion diameters meets the PD criterion that the case is determined as PD. This is because there is recognition that most lesions do not truly "disappear" but are only delineated by the resolution limitations of the imaging modality used.

When there is a possibility that the lesion is a new lesion but cannot be confirmed, it is not considered a new lesion, and it will continue to be evaluated at the next imaging examination. When it is confirmed that the lesion is a new lesion from re-examination of the image exam results, the appearance of the new lesion is defined as the date of the image examination when the new lesion was confirmed.

11.1.9. Overall response

The overall response is determined according to Table 11.1.9a below based on the combination of the efficacy on the target lesions, the efficacy on the non-target lesions, and whether or not any new lesions have appeared. The overall response in the absence of target lesions at baseline is determined according to Table 11.1.9b according to the efficacy on non-target lesions, and whether or not any new lesions have appeared.

Table 11.1.9a Overall response at each time point: when target lesions are present (doesn't depend on whether or not non-target lesions are present)

Target lesion	Non-target lesion	New lesion	Overall efficacy
CR	CR	No	CR
CR	Non-CR/non-PD	No	PR
CR	NE	No	PR
PR	Non-PD or NE	No	PR
SD	Non-PD or NE	No	SD
NE	Non-PD	No	NE
PD	Any	Yes or no	PD
Any	PD	Yes or no	PD
Any	Any	Yes	PD

Table 11.1.9b Overall response at each time point: when only non-target lesions are present

Non-target lesion	New lesion	Overall efficacy
CR	No	CR
Non-CR/non-PD	No	Non-CR/non-PD
Defective evaluation	No	NE
PD (clear worsening)	Yes or no	PD
Any	Yes	PD

11.1.10. Best Overall Response

The overall response is "good" in the order of CR > PR > SD > PD > NE, and the best overall response is determined according to the following criteria. When it corresponds to the definitions of multiple classifications, it is classified as the better one in the order of CR > PR > SD > PD > NE. However, when there are no target lesions at baseline, the best overall response does not use PR, and uses non-CR/non-PD instead of SD.

- **Complete Response (CR):**

When an overall response of CR is obtained two or more times continuously at intervals of 4 weeks (28 days) or more.
Furthermore, the day when the second overall response of CR is confirmed and the best overall effect of CR is determined is the day of confirmed CR.

- **Partial Response (PR):**
  When an overall response of PR or better (CR or PR) is obtained two or more times continuously at intervals of 4 weeks (28 days) or more.
  Furthermore, the day when the second overall response of PR is confirmed and the best overall effect of PR is determined is the day of confirmed PR.
- **Stable Disease (SD):**
  In the case where neither CR nor PR is obtained as the best overall response, and there is at least one overall response of SD or better.
  However, the shortest period of SD is 6 weeks from the start of protocol treatment.
- **Progressive Disease (PD):**
  When the overall response is PD without corresponding to a best overall response of either CR, PR, or SD.
- **Not Evaluable (NE):**
  When all of the overall responses are NE.

12. Statistical parameters

12.1. Definition of the analysis sets

We define the following analysis sets used in this study: regular monitoring, interim analysis, main analysis, additional analysis, and final analysis.

12.1.1. All enrolled cases

Among patients enrolled according to "5.1. Enrollment procedures," the population excluding duplicate enrollments and mis-enrollments is considered as "all enrolled cases."

12.1.2. Full Analysis Set (FAS)

The Full Analysis Set (FAS) is established as the largest analysis population among all enrolled patients, excluding patients (non-eligible cases) who have been confirmed post facto not to meet the "4._Patient selection criteria" after having received one or more medications. An FAS for the entire study and an FAS for each drug cohort are established.

12.1.3. Safety Analysis Set (SAS)

Out of all registered cases, the population of patients to whom medication has been administered one or more times is defined as the Safety Analysis Set (SAS). An SAS for the entire study and an SAS for each drug cohort are established.

12.2. Definition of the endpoints

12.2.1. Primary endpoint

The primary endpoint is the response rate based on the best overall effect up to 16±1 weeks after the start of treatment among patients with measurable lesions in the FAS for each drug cohort. Subjects who do not achieve CR/PR by 17 weeks after the start of treatment, and if CR or PR is confirmed for the first time thereafter, are not included in the primary endpoint numerator.

12.2.2. Secondary endpoints

1) Overall survival (OS)

The period from the date of enrollment to the date of death due to any cause

- In the surviving cases, the last survival confirmation date is considered to terminate the case (a survival confirmation can also be made via telephone communication, but it must be recorded in the chart).
- For cases lost to follow-up, termination of the case is made on the last day survival was confirmed before being lost to follow-up.

2) Progression-free survival (PFS)

Calculated as the number of days from the enrollment date as the start date to the date on which exacerbation is determined or to the date of death due to any cause, whichever is earlier.

- "Progression" includes both PD (progression) based on the imaging exam in "11.1.10. Best overall effect" and exacerbation (progression) of the underlying disease (clinical exacerbation) that cannot be confirmed by an imaging exam. The date on which the imaging examination was performed is taken as the exacerbation date if the exacerbation is determined based on the imaging exam, and the clinical judgment date is taken as the exacerbation date in the case of clinical exacerbation. If the tumor size becomes extremely small, etc., a determination of PD will be made according to the criteria for determining efficacy, but even if it is clinically judged as "apparently not exacerbated," a determination of PD according to the efficacy criteria will have priority (in this case, whether or not to continue administration of the study drug will prioritize the clinical judgment). Moreover, even if a determination of PD is not made according to the criteria for determining efficacy, if there is a clear judgment of clinical exacerbation, the

clinical judgment is given priority.

- In surviving cases that are not determined to be exacerbation, the case is terminated on the last day (the date of final confirmation of progression-free survival) when there is no clinical exacerbation (confirmed progression-free by image inspection and specimen inspection is not mandatory, and a confirmation of clinical non-progression can be made in an outpatient clinic, etc. Telephone communication only is not acceptable. If information about exacerbation or no progression is obtained during a hospital transfer or a medical institution referral, etc., the document(s) providing the medical information that describe the basis of the diagnosis must be received and retained. In this case as well, telephone contact only is not acceptable).
- Events and terminations are treated the same as in the case of chemotherapy discontinuation due to reasons such as toxicity or patient refusal, etc., and also when another treatment is added as a post-treatment. In other words, the case is not terminated at the time when treatment is discontinued or on the day that the post-treatment starts.
- When the diagnosis of exacerbation is based on imaging diagnosis, the event is not the exam date for “suspicion based on imaging” but rather the “exam date” for an imaging exam for which "confirmation" is obtained at a later date. If the case is determined to be clinically exacerbated regardless of diagnostic imaging, the event is considered to be the day on which the case is determined to be exacerbated.
- The occurrence of secondary cancer (including metachronous double cancer and metachronous multiple cancer) is neither an event nor a termination, and it is considered as progression free survival until other events are observed.

3) Disease control rate

The proportion of patients within the FAS not classified as PD or NE in “11.1.10. Best overall effect” as determined by the facility.

4) Incidence of adverse events

Percentage of patients who developed each adverse event in SAS.

In addition, the frequency of the worst grade according to CTCAE v5.0-JCOG is determined for each adverse event.

12.3. Main analysis and judgment criteria

12.3.1. Primary endpoint analysis

In this study, the primary endpoint is the response rate based on the best overall effect up to 16±1 weeks after the start of treatment among patients with measurable lesions in the FAS for each drug cohort. This study is not intended to test hypotheses set in advance, but from the viewpoint of feasibility under the patient-proposed healthcare services, with each drug cohort expected to have up to 50 patients who have measurable lesions as subjects. The main analysis will be conducted for each drug cohort when those 50 patients have been enrolled and the 50th patient has completed the efficacy evaluation up to the time point of 16±1 weeks. For patients who were determined to have measurable lesions at the time of enrollment but for whatever reason the determination was changed to no measurable lesions after treatment started, the best overall response is given as NE.

In this study, it is decided to use Bayesian inference, and if the probability that the posterior distribution of the response rate exceeding the threshold response rate of 60% exceeds 95%, it is determined that the medicine can be expected efficacious. If the corresponding drug is provided free of charge by a company, a report is to be prepared summarizing the information on the drug, and the results of the research shared with the corresponding company, which can then explore the pathway to regulatory approval including clinical trials.

We adopt a beta (0.6, 1.4) with an expected value of 30% as the prior distribution.

In line with the above analysis, 95% confidence intervals (two-sided) are also calculated using an accurate method from frequentist theory (Clopper and Pearson method) based on a binomial distribution for the interval estimates of the response rate.

Furthermore, an interim analysis (12.4.) will be conducted according to the separately established policy based on the data from the time of regular monitoring conducted twice a year.

Even if more than 50 eligible patients with measurable lesions can be enrolled, the same analysis and response as for the main analysis will be performed, and an updated version of the Final Report will be created as an additional analysis of the primary endpoint for each 50 patients, and the results will be examined.

12.3.2. Analysis of efficacy secondary endpoints

Among the secondary endpoints, the efficacy endpoints are overall survival, progression-free survival, and disease control rate. The efficacy analysis set is the FAS for each drug cohort, and analysis is performed for each drug cohort at the time of main analysis or the subsequent primary endpoint analysis. Survival curves, median survival time, and annual survival rates are estimated for each survival time using the Kaplan-Meier method. The 95% confidence intervals (two-sided) are estimated using the Brookmeyer and Crowley method for the respective median survival times. The 95% confidence intervals (two-sided) are calculated using Greenwood's formula for interval estimation of the annual survival rates. An accurate method (Clopper and Pearson method) based on a binomial distribution is used for interval estimates for the disease control rate.

12.3.3. Analysis of safety secondary endpoints

Among the secondary endpoints, the safety endpoints are the incidence of adverse events. The safety analysis set is SAS for each drug cohort, and analysis for each drug cohort is performed at the time of main analysis or at the time of additional analysis of the primary endpoint thereafter. The frequencies of the worst grade for each adverse event are summed when estimating the incidence of adverse events. An accurate method (Clopper and Pearson method) based on a binomial distribution is used for 95% confidence interval estimates of the incidence. Furthermore, the safety in the FAS is to be analyzed as needed.

12.4. Interim analysis

According to the appearance of biomarkers, there is a high possibility of difficulties in conducting research which might be caused by the difference between the prior plans and the actual research progress compared to the usual clinical trials, such as the possibility of the prospective accumulation period being uncertain due to the small number of patients. It is a problem from an ethical standpoint that a particularly ineffective treatment might be continued without being stopped by unsatisfactory patient accrual. It is important to incorporate into the research plan a method to deal with the uncertainty and difficulties in conducting the research.

Since the Bayesian design allows a more flexible statistical evaluation than does the hypothesis test of the frequentist theory, in addition to the main analysis in this study, the Bayesian aspect is also monitored in the intermediate analysis.

When ≥ 15 patients are enrolled for each drug cohort, response rates based on the best overall response are assessed based on a Bayesian analysis up to 16±1 weeks after treatment during semiannual regular monitoring (14.1 “Periodic monitoring”), and the need for discontinuation due to inefficacy or discontinuation due to efficacy is assessed to determine whether the drug cohort is considered to be continuable.

The information required for discontinuation due to inefficacy or discontinuation due to efficacy is shown in Table 12.4.

We adopt a beta (0.6, 1.4) with an expected value of 30% as the prior distribution relating to response rates in this trial. If the data actually obtained is significantly below or above this prior expectation, the necessity of discontinuation due to inefficacy or discontinuation due to efficacy, respectively, will be considered by using a threshold of 20% as an indication of “much below prior expectation” and a threshold of 60% is set as an indication of “significantly exceeding prior expectations.” If the probability of exceeding the 20% threshold is less than 5% when calculating the posterior distribution of the response rate, the necessity of early discontinuation due to inefficacy is examined. Moreover, f the probability of exceeding

the 60% threshold is ≥ 95% when calculating the posterior distribution of the response rate, the necessity of early discontinuation due to efficacy is examined.

If necessary, subgroups by combining drugs with biomarkers and cancer types can also be considered.

Table 12.4: Decision table for early discontinuation (discontinuation due to inefficacy or discontinuation due to efficacy) in each drug cohort

<Denominator> Number of cases that can be evaluated for response	<Numerator> Number of cases to consider discontinuation due to inefficacy (The expected value of the prior distribution is 30%, and the maximum number of response cases in which the probability that the posterior distribution exceeds the 20% threshold is less than 5%)		<Numerator> Number of cases to consider discontinuation due to efficacy (The expected value of the prior distribution is 30%, and the minimum number of response cases in which the probability that the posterior distribution exceeds the 60% threshold is ≥ 95%)	
5	-	-	-	-
6	-	-	-	-
7	-	-	7	1.000
8	-	-	8	1.000
9	0	0.000	9	1.000
10	0	0.000	10	1.000
11	0	0.000	10	0.909
12	0	0.000	11	0.917
13	0	0.000	12	0.923
14	0	0.000	12	0.857
15	0	0.000	13	0.867
16	0	0.000	14	0.875
17	0	0.000	15	0.882
18	1	0.056	15	0.833
19	1	0.053	16	0.842
20	1	0.050	17	0.850
21	1	0.048	17	0.810
22	1	0.045	18	0.818
23	1	0.043	19	0.826
24	1	0.042	19	0.792
25	1	0.040	20	0.800
26	2	0.077	21	0.808
27	2	0.074	21	0.778
28	2	0.071	22	0.786
29	2	0.069	23	0.793
30	2	0.067	23	0.767
31	2	0.065	24	0.774
32	2	0.063	25	0.781
33	3	0.091	25	0.758
34	3	0.088	26	0.765
35	3	0.086	27	0.771
36	3	0.083	27	0.750
37	3	0.081	28	0.757
38	3	0.079	29	0.763

39	3	0.077	29	0.744
40	4	0.100	30	0.750
41	4	0.098	31	0.756
42	4	0.095	31	0.738
43	4	0.093	32	0.744
44	4	0.091	33	0.750
45	4	0.089	33	0.733
46	4	0.087	34	0.739
47	5	0.106	35	0.745
48	5	0.104	35	0.729
49	5	0.102	36	0.735
50	5	0.100	37	0.740
51	5	0.098	37	0.725
52	5	0.096	38	0.731
53	6	0.113	39	0.736
54	6	0.111	39	0.722
55	6	0.109	40	0.727
56	6	0.107	41	0.732
57	6	0.105	41	0.719
58	6	0.103	42	0.724
59	7	0.119	43	0.729
60	7	0.117	43	0.717

12.5. Final analysis

In this study, the main analysis is performed for each medicine, and additional analysis on the primary endpoint can be carried out more than once for some medicines. For this reason, the final analysis of the medicine will be conducted when patient enrollment for each drug is completed under the framework of this research plan. After completing the follow-up on the last enrolled patient for the corresponding drug, analysis is performed on all endpoints after the data are confirmed.

Finally, when the entire study is completed, a summary report is to be compiled that presents a Final Report, citing the final analysis results for each drug cohort.

12.6. Study period and number of enrollments

The number of enrollments in this study is not set based on clinical hypothesis. A contract is to be concluded between the National Cancer Research Center and the pharmaceutical companies that provide each drug, setting a certain upper limit on the number of enrollments for each drug. To allow for a certain level of exploratory examination for each medicine, this upper limit is determined based on a consideration of 50 cases (enrolled cases with measurable lesions), and when this number of registrations is reached, a consultation will be held with the Patient-Proposed Healthcare Service Evaluation Committee to consider the necessity of changing the plan. When the efficacy is to be determined by carrying out a Bayesian analysis at the time of main analysis, the effective sample size (ESS) is 49.1 when 50 cases are enrolled, and the effect of the prior distribution (beta (0.6, 1.4)) is small.

As it is ethically undesirable to expand administration to a large number of patients when exploring the efficacy is difficult, it is recommended that 30 patients with no measurable lesions be used as a guide. If fewer than 50 patients with measurable lesions are enrolled at a time when 30 patients with no measurable lesions have been enrolled, enrollment will be continued with only patients having measurable lesions. Conversely, once 50 patients with measurable lesions have been enrolled, the enrollment of patients with no measurable lesions will end.

However, even before the upper limit is reached, it is ethically unacceptable to continue administration of a drug that is ineffective for treatment. In addition, if there is a hope for effectiveness, it is necessary to proceed to the planning of sponsor-initiated clinical trials or investigator-initiated clinical trials at an early stage with the objective of expanding the indication and qualifying the medicine for insurance coverage. For this reason, a separately defined interim analysis is to be conducted, and decision rules are to be set that consider discontinuation due to inefficacy or discontinuation due to efficacy for each drug cohort. As a result, while establishing a framework to provide promising drugs to patients who have no existing therapies and have not yet been subjects in other targeted clinical trials, etc., continuation of unethical treatments and continuation of patient-proposed healthcare service that are pointless will be avoided.

On the other hand, setting the abovementioned number of enrolled cases is a setting for performing certain evaluations persistently, and when the pharmaceutical companies continue to provide drugs based on information at the time of regular monitoring, intermediate analysis results, and main analysis results, etc., the number of enrollments changes as appropriate.

The planned enrollment period for this study is 5 years, and the follow up period is 3 years after the enrollment for all patients is completed. Enrollment for each drug starts and ends within the scheduled enrollment period. In light of the social attitudes such as the availability of off-label medications, it will be considered before the end of the five-year scheduled enrollment period whether to change the protocol content to extend the enrollment period.

For reference, according to TOP-GEAR analysis of solid cancers at the National Cancer Center Hospital up to the present, 2.77 gene mutations per specimen have been detected on average. The frequency of candidate gene abnormalities is expected to be approximately 10-40%. It is estimated that 10-15% of these will be recorded as actionable mutations.

Therefore, although the number of patients enrolled in this study is affected by the frequency of biomarker expression for each cancer type, several hundred cases/year are expected depending on the number of medicines included. However, because there is an upper limit on the number of enrollments depending on the drug, enrollment might be completed early. The number of annual enrollments in the entire study depends on the number of drugs included, as this study includes multiple drugs

simultaneously.

Scheduled enrollment period: 5 years (scheduled from September 1, 2019 – August 31, 2024)
Tracking period: 3 years after completion of enrollment (Sept. 1, 2024 – Aug. 31, 2027)
Analysis period: 1 year (Sept. 1, 2027 – Aug. 31, 2028)
Total study period: 9 years (September 1, 2019 – August 31, 2028)

12.7. Early discontinuation

12.7.1. Early discontinuation of a drug cohort

In this study, early discontinuation for a drug might be performed in the following cases.

If the drug list included in this study changes as a result of early discontinuation, the Study Representative Physician/Study Secretariat will change the protocol content (see 13.9.).

1） Early discontinuation according to the interim analysis (discontinuation due to inefficacy/discontinuation due to efficacy)
2） Early discontinuation due to adverse events
3） Early discontinuation for other reasons

1) Early discontinuation according to the interim analysis (discontinuation due to inefficacy/discontinuation due to efficacy)

In this study, based on the criteria described in “12.2. Interim analysis,” ≥ 15 patients with measurable lesions were enrolled for each drug cohort, and as mentioned above, based on the response ratio from periodic monitoring performed semiannually, a determination is to be made based on Table 12.4 whether or not to consider early discontinuation (discontinuation due to inefficacy).

In addition, if there is a hope for effectiveness, it is necessary to proceed to the planning of sponsor-initiated clinical trials or investigator-initiated clinical trials at an early stage with the objective of expanding the indication and qualifying the medicine for insurance coverage (discontinuation due to efficacy).

2) Early discontinuation due to adverse events (discontinuation due to inefficacy)

If the adverse event is outside the permissible range, patient enrollment will be temporarily suspended, and this will be reported to the Efficacy and Safety Evaluation Committee who will consider early discontinuation of the drug cohort.

Specifically, when two or more treatment-related deaths are reported for each medicine, the rate of treatment-related deaths for that medicine exceeds 2% (2/80 = 2.5%), and the Efficacy and Safety Evaluation Committee will deliberate on whether new patients can be enrolled and whether treatment can continue for the patients already enrolled relating to the corresponding drug cohort. Furthermore, when it is possible to enroll more than 80 patients in a drug cohort, the Efficacy and Safety Evaluation Committee will similarly deliberate when it becomes clear that the treatment-related death rate exceeds 2%. In addition, even if the above conditions are not met, the cases in which the Study Representative Physician/Study Secretariat determines that the serious adverse event occurrence profile reported in the periodic monitoring report deviates significantly from the content described in the attached document, the Efficacy and Safety Evaluation Committee will deliberate on whether new patients can be enrolled and whether treatment can continue for the patients already enrolled relating to the corresponding drug. These details are to be reported to the Medical Economics Division, Health Insurance Bureau, of the Ministry of Health, Labour and Welfare.

If a recommendation for early discontinuation is issued by the Efficacy and Safety Evaluation Committee, the Study Representative Physician/Study Secretariat will review the content of the recommendation and decide whether to proceed with early discontinuation.

3) Early discontinuation for other reasons

If it is judged that continuation of the drug cohort is difficult due to reasons other than 1) and 2) above, the Study Representative Physician/Study Secretariat will report that to the Efficacy and Safety Evaluation Committee. In the case of an early discontinuation recommendation issued by the Efficacy and Safety Evaluation Committee, the early discontinuation will proceed.

12.7.2. Discontinuation of the study

Since this study corresponds to a specific clinical study under the Clinical Research Act, the Study Representative Physician will submit a written notice of discontinuation to the Certified Clinical Research Review Board within 10 days from the day when the study early discontinuation decision is made, and a discontinuation notification will be submitted to the Minister of Health, Labour and Welfare. The Study Representative Physician is to promptly inform the Principal Investigator in writing of the details of the decision to discontinue the study early, and the Principal Investigator who receives a report on the early discontinuation of the study is to inform the Study Center Director in writing without delay of the details of the early discontinuation of the study.

In addition, since this study is a patient-proposed healthcare service, when the study is discontinued, it will be reported to the Patient-proposed Healthcare Service Evaluation Committee to request a determination as to whether or not to continue the patient-proposed healthcare service.

13. Ethical matters

13.1. Patient protection

All researchers related to this study will conduct the research according to the "Helsinki Declaration" (Translated by the Japan Medical Association),[1)] the "Clinical Research Act" (Law No. 16 of 2017),[2)] the "Clinical Research Act Enforcement Regulations" (Ministry of Health, Labour and Welfare Ordinance No. 17 of 2018) and related notices, and the "Health Insurance Act and the Law for Assuring Healthcare for the Elderly" (in force as of March 4, 2016, based on Health Policy Bureau (HPB) notification no. 0304.3, Pharmaceutical Safety Bureau (PSB) notification no. 0304.1, and Health Insurance Bureau (HIB) notification no. 0304.18) and the detailed regulations thereof.[3)]

1） http://dl.med.or.jp/dl-med/wma/helsinki2013j.pdf
2） http://www.mhlw.go.jp/stf/seisakunitsuite/bunya/0000163417.html
3） http://www.mhlw.go.jp/stf/seisakunitsuite/bunya/0000114800.html

Prior to the start of this study, the Principal Investigator is to receive the approval of the Study Center Director after hearing the opinion of the Certified Clinical Research Review Board regarding the implementation of this study, and the implementation plan[*1] must be submitted to the Minister of Health, Labour and Welfare. In addition, it must be discussed and approved by the Patient-Proposed Healthcare Service Evaluation Committee.

*1 "Implementation plan" refers to the "Plan according to Form No. 1 as stipulated in Article 39 of the Clinical Research Act Enforcement Regulations (Ministerial Form No. 1)"

13.2. Informed consent

13.2.1. Explanation to patients

Prior to the enrollment, the Principal Investigator and Co-investigators will deliver to the patient himself/herself the explanatory document approved by the Certified Clinical Research Review Board, and will verbally explain the following content.

1. Introduction: this research involves a clinical research study and patient-proposed healthcare service
2. Treatment of your disease: explanation of the name and pathology of the disease
3. Cancer genome-based healthcare in Japan
4. Gene panel testing and subsequent treatment
5. Purpose of the clinical study
6. Duration and number of participants in the clinical study
7. Subjects and methods in the clinical study: details of the protocol treatment
8. Side effects: expected adverse events, complications, extent and frequency of sequelae, and countermeasures when they occur
9. Discontinuation of the study treatment
10. Expected benefits and expected disadvantages from participating in clinical research
11. Treatment when not participating in clinical research
12. Costs
13. Compensation for health damage
14. Status progress and results of this clinical research: including methods for disclosing information about the study
15. Medical institutions participating in this clinical research
16. Funding of and conflicts of interest in this clinical research: the existence and details of involvement of drug manufacturers and distributors, etc.
17. Handling of intellectual property rights
18. Privacy protection (personal information): including explanations about the acceptance of facility audit visits such as how "the head of the medical institution can grant permission to medical personnel from other medical institutions to read medical histories directly for quality control"

19. Secondary use of data and additional research
20. Ethical review of this clinical research: name of the Certified Clinical Research Review Board and name of the committee concerned with receiving inquiries and complaints and the relevant contact information
21. Voluntary consent and withdrawal of consent
22. Requests when participating in this clinical research
23. Contact information

13.2.2. Consent

The study is to be explained and the patient given enough time to think about and confirm that he/she has well understood the content of the study, and then is asked to participate in the study. If the patient agrees to participate in the study, this will be reviewed for approval by an Certified Clinical Research Review Board, and the patient himself/herself will sign the relevant forms using "19. Appendix: Explanatory Document/Informed Consent Form." The Principal Investigator or Co-investigator should confirm that the written consent contains the name of the physician who gave the explanation, the date of the explanation, the name of the patient who has received the explanation, and the date of consent. If the patient cannot sign because of neurological symptoms, etc., it is possible to obtain a signature from a witness (a substitute signatory) with the patient's consent. In addition, when the patient is a minor, consent and signature are obtained from a representative (the legal guardian as a rule).

Two copies of the written consent will be prepared, one handed to the patient and one to be kept by the facility coordinator. The originals should be stored in the medical records or in a retention area defined by the medical institution.

13.2.3. Informed assent

Even if consent for participation in this study has been obtained from the patient's legal representative, the patient should receive explanations commensurate to his/her ability to understand, and the assent of the patient himself/herself should be obtained wherever possible. The physician in charge will explain the study to the patient and confirm the patient's willingness to participate in the study, with the following categories as a guide and using appropriate assent documents.

1） 15 to 17 years of age: provide explanations to the patient and his/her legal representative using informed consent documents for adults. The patient should sign and date the assent section of the consent document for adults, and the legal representative should sign and date the legal representative section of the same document.

2） Roughly 7 to 14 years of age (elementary to middle school students): the patient should be informed with an appropriate assent document, and should sign and date the assent document by the patient. In addition to this, informed consent documents for adults should be used to provide explanations to the legal representative, and the legal representative should sign and date the legal representative section of the informed consent documents for adults.

3） Children under 7 years of age (preschoolers): only the legal representative's consent is required. Use of assent documents is not required.

13.2.4. Responding to inquiries and consultations after consent is given

In principle, if a patient or his/her family members have inquiries about this research after enrollment, the Principal Investigator or Co-investigator of the patient's medical institution will respond. If the response method is unclear, depending on the content of the consultation, responses may be obtained in consultation with the Study Representative Physician, Study Secretariat, Data Management Supervisor, or the Coordination Office.

13.2.5. Consent withdrawal

If the patient proposes to cancel his/her consent for study participation after consent for study participation has been obtained, the consent will be withdrawn. When the patient is a minor, if the patient and his/her representative (the legal guardian as a rule) propose to cancel the patient's consent for study participation after consent for study participation has been obtained, the consent will be withdrawn. If the

intentions of the two parties are different, as a rule the patient's intention is given priority, and the circumstances of the decision will be noted in the medical records. Consent withdrawal means withdrawal of consent to participate in the study, and it is distinguished from a refusal to continue the protocol treatment (see [1] below). If a withdrawal of consent is declared, clarify either of the following [2] or [3] and promptly contact the Study Secretariat.

In the case of withdrawal of consent described under [2], the data management supervisor will discontinue later follow-up requests according to the protocol. In case [3], the patient's data is to be deleted from the database once it is confirmed that all consent has been withdrawn.

Procedures for discontinuing follow-up requests to the patient and deletion of patient data will be separately determined in the procedure manual, and reports are to be made to the Study Representative Physician and Study Secretariat that the respective tasks have been completed.

① Patient refusal: refusal to continue the protocol treatment (follow up continues).

② Consent withdrawal: withdrawal of consent to participate in the study, which renders impossible all subsequent treatment according to the protocol and further follow-up. Research uses of the data obtained prior to consent withdrawal is permitted.

③ Complete consent withdrawal: withdrawal of consent to participate in the study, and disallowance of the use of all data from the study participation period including information at the time of enrollment.

13.3. Protection of personal information and patient identification

It is recognized that private information such as personal information and medical care information should be strictly protected and treated carefully under the principle of respect for personal privacy, and thorough management measures will be taken to strive to protect privacy.

13.3.1. Laws and standards

The following laws and regulations are to be complied with.

- Clinical Research Act (Law No. 16 of 2017), Clinical Research Act Enforcement Regulations (Ministry of Health, Labour and Welfare Ordinance No. 17 of 2018) and related notifications
- Personal Information Protection Law (Law No. 57, in force as of May 30, 2003; final revision: Law No. 44, in force as of April 1, 2022)
- Helsinki Declaration (Translated by the Japan Medical Association)
- Health Insurance Act and the Law for Assuring Healthcare for the Elderly (in force as of March 4, 2016, based on Health Policy Bureau (HPB) notification no. 0304.3, Pharmaceutical Safety Bureau (PSB) notification no. 0304.1, and Health Insurance Bureau (HIB) notification no. 0304.18) and the detailed regulations thereof

13.3.2. Purpose of use of personal information, parameters used, and methods of use

1) Purpose of use

The purpose of this study is “o build evidence to provide appropriate treatment to more patients.” The patient's personal information is used for the purpose of “properly managing the acquired information to obtain correct results from clinical research.”

2) Parameters used

The following information used to identify individuals is considered to be the minimum required for patient identification and referral.

Date of birth (or year and month of birth), medical record number (patient number), enrollment number

That is, there is no information other than the above, such as the patient's name, that can identify individuals from the study center to the data management supervisor, and any such notification that is made erroneously will be discarded regardless of the recording or storage medium, or stored after performing appropriate processing such as masking and making it unreadable.

3) Methods of use

Patient's personal information is collected by researchers at medical institutions and entered into

various CRF forms in EDC.

In addition, the exchange of inquiries between data management personnel and researchers at medical institutions regarding various CRFs that include personal information, etc. for confirming the accuracy of the information collected can only be done via EDC or by mail or personal delivery. When exchanging an inquiry by email, use is made only of the enrollment number, with a higher level of anonymity, and date of birth, etc. are not used.

13.3.3. Creating records of specimens, information, etc. provided to participating facilities

The Principal Investigator at each facility will create records of the patients enrolled in this study. The following matters are stipulated by the Ministry of Health, Labour and Welfare ordinance (Clinical Research Act Enforcement Regulations, Article 53).

- Matters that identify the subjects of clinical research
- Matters that relate to medical treatment and examination for subjects of clinical research
- Matters concerning participation in clinical research
- In addition to the abovementioned, documents required to conduct the clinical research

13.3.4. Retention of specimens, information, etc.

Specimens and information, etc. from enrolled patients relating to this research will be stored in accordance with “Article 53 of the Clinical Research Act Enforcement Regulations (MHLW Ordinance No. 17 of 2018).” The retention period for the records relating to this research and the retention period for the original materials at the participating facilities will be 5 years from the day when this research was completed. It is recommended to retain these for as long as possible after the deadline. If the specimens and information related to this research are to be disposed of after the retention period has passed, they are anonymized and disposed of.

The retention period for data collected by the data management supervisor is semipermanent in view of the possibility of long-term tracking and secondary research use.

If the patient and his/her representative (the legal guardian as a rule) propose to cancel the patient’s consent for study participation after consent for trial participation has been obtained, the consent will be withdrawn. If the intentions of the two parties are different, then the patient's intention is given priority.

13.3.5. Management of anonymization and the correspondence table

Information that can clearly identify persons individually, such as patient names, is not collected, but rather individuals are identified using enrollment numbers. etc. (anonymization). So that information such as the patient's name, etc. by which the patient can be clearly identified individually, and the registration numbers in the correspondence table (not necessarily in the form of “table”) can be present in position in each study center, and thus ensure the identification of enrolled patients, appropriate management is to be performed according to the policies of the study center.

13.3.6. Secondary use of data

Secondary use of the data obtained in this research (such as in a meta-analysis) domestically or overseas is only possible with approval following an appropriate research review. However, any provision of data externally (for meta-analysis, etc.) must be done in a form in which the individual cannot be identified. For such secondary use to be possible, consent must be obtained in writing in the informed consent document. If secondary use is to be made of data, a notice to that effect must be posted or made available on a readily accessible home page, etc. to ensure that patients have the possibility of rejecting it.

13.3.7. Safety Management Director

The Data Management Supervisor is to decide on a Privacy Protection Management Director and a Privacy Protection Supervisor who will implement various security measures to minimize the risk of information leakage when using personal information, etc.

13.3.8. Responding to disclosures, etc. of patient information

In principle, the only person who should ask the patient himself/herself to disclose, etc. privacy-related information is a researcher of the patient's medical institution (Principal Investigator or Co-investigator).

13.3.9. Receiving inquiries

General inquiries and complaints regarding privacy protection, etc. will be accepted by mail, email, or fax as below.

Contact information: Privacy Protection Supervisor

Mailing address: 5-1-1, Tsukiji, Chuo-ku, Tokyo, JAPAN 104-0045
Clinical Research Support Office, National Cancer Center Hospital
Email: NCCH1901_office@ml.res.ncc.go.jp
FAX: 03-3542-3374

13.3.10. Provision of data, etc. to third parties

Research data may be provided to companies for use in documents for the purpose of preparation for the Study Group on Unapproved and Off-label Drugs of High Medical Need or for sponsor-initiated clinical trials or investigator-initiated clinical trials. It is to be provided in a form in which individuals cannot be identified. Informed consent must be obtained as described above for the informed consent document when this information is to be provided.

13.4. Protocol compliance

Researchers participating in this study will follow this protocol as long as doing so does not compromise patient safety and human rights.

13.5. Application to the Certified Clinical Research Review Board and application to Patient-Proposed Healthcare Service Evaluation Committee, notification of the implementation plan

At the time of conducting this research, approval must have been obtained from the Certified Clinical Research Review Board and the Patient-Proposed Healthcare Service Evaluation Committee, and permission to conduct the research obtained from the Study Center Director. In addition, prior to the start of research, the implementation plan[*1] is to be submitted to the Minister of Health, Labour and Welfare, and the trial information must be published on the Japan Registry of Clinical Trials （jRCT）[*2]. The Principal Investigator is responsible for the application to the Certified Clinical Research Review Board, for submitting the implementation plan to the Minister of Health, Labour and Welfare, and for registering the trial in jRCT.

*1 Ministerial Form No. 1 as stipulated in Article 39.1 of the Clinical Research Act Enforcement Regulations

*2 A database (Japan Registry of Clinical Trials) maintained by the Ministry of Health, Labour and Welfare as stipulated in Article 24.1 of the Clinical Research Act Enforcement Regulations https://jrct.niph.go.jp/

13.6. Periodic report and performance report

1） Periodic reporting as a clinical research method

The Study Representative Physician submits a periodic report on the progress of the research, the occurrence of adverse events, and management of conflicts of interest, etc. to the Director of the Study Center to which he/she belongs, and also reports to the Certified Clinical Research Review Board. Calculating from the jRCT publication date, reporting is performed every year within 2 months after the termination of the corresponding period.

When a report is made to the Certified Clinical Research Review Board, the Study Representative Physician will promptly provide information to that effect to the Principal Investigators of the other participating medical institutions. The Principal Investigators who have received the information will

promptly report the content of the provided information to the Director of the Study Center to which they belong.

The Study Representative Physician will report to the Minister of Health, Labour and Welfare on the status of implementation of specific clinical research. Through the periodic reports made to the Certified Clinical Research Review Board, a report on whether or not to continue the specific clinical research can be made within 1 month as calculated from the day the results were obtained.

2） Periodic reports on patient-proposed healthcare services

The Study Representative Physician compiles the results from the Study Centers and reports to the Local (Branch) Health and Welfare Bureau.

3） Performance reports on patient-proposed healthcare services

The Study Representative Physician will submit performance report results to the Medical Economics Division, Health Insurance Bureau, of the Ministry of Health, Labour and Welfare every 3 months for a 6-month period calculated from the jRCT publication date, and thereafter every 6 months. The content to be reported is to be the number of patient enrollments, endpoint-related items, adverse events, and other items deemed necessary at the discretion of the Study Representative Physician. In addition, if adverse event occurrences or event occurrences are observed after the protocol treatment has been completed, these will be reported to the Medical Economics Division, Health Insurance Bureau, of the Ministry of Health, Labour and Welfare as performance reports at the discretion of the Study Representative Physician.

13.7. Procedures performed by the Principal Investigator at each study center

After obtaining approval from the Certified Clinical Research Review Board, the Principal Investigator at each study center will use the complete set of documents received from the Study Representative Physician (a notification letter on the results of the review by the Certified Clinical Research Review Board and documents provided to the Certified Clinical Research Review Board) to obtain a Research Conduct Approval from the Study Center Director to which he/she belongs. After obtaining it from the Director, the Principal Investigator will promptly send a copy of the Research Conduct Approval from the relevant medical institution to the Study Representative Physician by email.

In addition, the Principal Investigator who has received the provided information on the details of the implementation plan submitted to the Minister of Health, Labour and Welfare by the Study Representative Physician will promptly report to the Study Center Director to which he/she belongs that the implementation plan has been submitted.

13.8. Research Conduct Approval for each study center

The regulations of each medical institution are to be followed for the procedure for obtaining a Research Conduct Approval from the Director of the affiliated medical institution.

When the medical institution's Research Conduct Approval is sent by email to the Study Representative Physician, the Principal Investigator sends a copy. The original Research Conduct Approval is retained by the Principal Investigator and a copy is retained by the Study Representative Physician.

The informed consent documents for patients approved by the Certified Clinical Research Review Board cannot be changed except for the contact information for the facility and items specified in advance. A common protocol must be used for all medical institutions, so it is not acceptable for the protocol content to be changed by a medical institution. If it becomes necessary to change the content of the protocol or informed consent document, the protocol and informed consent document used in all medical institutions must be changed. When the Study Center Directors request modifications of the protocol/informed consent document, a consultation is to be had with the Study Representative Physician or Study Secretariat.

13.9. Changing the protocol content

13.9.1. Procedure for changing the protocol content

Since this study is a specific clinical study to be conducted as a patient-proposed healthcare service, changes in the protocol content are to be examined and approved by the Certified Clinical Research Review Board prior to any changes entering into force, and the changed content must be reviewed and approved by the Patient-Proposed Healthcare Service Evaluation Committee, and must be reported to the Minister of Health, Labour and Welfare.

After approval by the Certified Clinical Research Review Board and the Patient-Proposed Healthcare Service Evaluation Committee, approval of the changed content must be obtained from the Study Center Directors of each medical institution. If permission is obtained, the Principal Investigator for each medical institution is to send a copy of the Director's permit to the Principal Investigators of each medical institution. After obtaining permission from the Study Center Directors of all the medical institutions, the changed protocol content can go into effect (during this time, the patient enrollment will not be stopped unless necessary). The actual effective date will be announced by the Study Representative Physician, and after the effective date, all participating institutions will conduct the study according to the revised content approved by the Certified Clinical Research Review Board.

If a protocol or informed consent document is amended or revised, the Study Representative Physician is to promptly distribute the most recent protocol or informed consent document to the staff engaged in the clinical research. Those engaged in the clinical research must always conduct the research according to the most recent protocol.

13.9.2. Decisions about when the original protocol content threatens patient safety

If it is found that the safety of the patient might be threatened by the protocol content prior to the change, such as inadequate treatment criteria, etc., the protocol content should promptly be changed. In that case, patient enrollment is temporarily suspended if necessary, but since it takes a certain amount of time for the protocol change to take effect, a systematic protocol deviation to improve the safety of the patient being treated is permissible. If a protocol deviation is made, that fact is to be listed in the monitoring report.

13.9.3. Informed consent to be re-obtained from the patient for a protocol change

With any change in the details of the study, the Principal Investigator and Co-investigators are to provide the enrolled patients with appropriate explanations (corresponding to changes in the protocol treatment, follow-up, etc.). In addition, if the Certified Clinical Research Review Board submits an opinion that re-consent by the enrolled patient is necessary, written consent is to be re-obtained.

13.10. Conflict of interest (COI) relating to the research staff

13.10.1. Managing COI relating to the research staff

The COI relating to this study will be managed in accordance with the "Guidance for conflict of interest management relating to the Clinical Research Act" (Health Policy Bureau (HPB) notification no. 0302.1 of March 2, 2018, from the Director, Research and Development Division, Health Policy Bureau, Ministry of Health, Labour and Welfare) * (hereinafter, the "Guidance") .

* http://www.mhlw.go.jp/stf/seisakunitsuite/bunya/0000163417.html

The format used for COI management is to be the most recent version of the Guidance.

- Conflict of Interest Management Standards: Form A
- Involved Companies Report: Form B
- Researcher Conflict of Interest Self-report: Form C
- Conflict of Interest Situation Confirmation Report: Form D
- Conflict of Interest Management Plan: Form E

1) Procedure for new application

Conflict of Interest Management Standards

① According to the Guidance, Conflict of Interest Management Standards (Form A) is to be adopted

Request for confirmation of conflict of interest

② Before applying to the Certified Clinical Research Review Board, the Study Representative Physician/Study Secretariat is required to manage the conflicts of interest from the information on medicines and medical devices specified as protocol treatment (refer to "19. Appendix: List of medicines"), identify the companies, etc. involved in the research that require conflict of interest management, and fill out Form B for the corresponding companies.

③ The Study Representative Physician/Study Secretariat is to send a complete set of the forms to the Principal Investigator at each study center, request confirmation of the content of Form B, and request the preparation of Form C and Form E. In addition, a complete set of the forms will be sent from the Study Representative Physician/Study Secretariat to those staff who correspond to the "Manager and Contact Person for Statistical Analysis" for the "Implementation Plan" and the "staff who perform research other than the Principal Investigator/Co-investigator" with the request that Form C and Form E be completed.

Confirmation of conflict of interest (participating facility)

④ The Principal Investigator for each study center will decide which Co-investigators are involved in this research. The Principal Investigator and Co-investigators will be the declarers of conflicts of interest for this study.

⑤ The Principal Investigator is to confirm whether there are any matters that fall under Q2 to Q5 of Form B among the forms received from the Study Representative Physician and the Study Secretariat, and to notify the Study Representative Physician/Study Secretariat of any items that must be reported within 1 week.

⑥ The Principal Investigator is to enter the information from the Study Representative Physician/Study Secretariat in the [Certifier of Conflict of Interest Self-report (Form C) required] * field of Form C, and request that the Co-investigators fill out Form C.

※ The information entered in Form C is automatically entered in Form E. This entered information must be consistent with the physician information described in "List of Co-investigators" (Clinical Research Act Unified Format 1) to be submitted together with Form E to the Certified Clinical Research Review Board, and any physician not listed there cannot be involved in this research.

⑦ The Principal Investigator and Co-investigators are to fill in the necessary information about the <u>relationships with the companies, etc. listed in advance in Form C</u>, and submit Form C to the Conflict of Interest Confirmation Department of their affiliated medical institution. The Principal Investigator is to submit Form A at the same time.

⑧ The Principal Investigator receives the provided conflict of interest confirmation results (Form D) for himself/herself and the Co-investigators from their affiliated medical institution.

⑨ The Principal Investigator is to confirm the content of Form A, Form B, and Form D for himself/herself and all of the Co-investigators, then create Form E and notify the Study Representative Physician/Study Secretariat.

Confirmation of conflict of interest (other than participating facilities)

⑩ The corresponding people will be the declarers of conflicts of interest in this study for the "Manager and Contact Person for Statistical Analysis" and the "staff who perform research other than the Principal Investigator/Co-investigator."

⑪ Among the forms received from the Study Representative Physician/Study Secretariat, the "Manager and Contact Person for Statistical Analysis" and the "staff who perform research other than the Principal Investigator/Co-investigator" are to fill in the necessary information about the <u>relationships with the companies, etc., listed in advance in Form C</u>, and submit Form A and Form C to the Conflict of Interest Confirmation Department of their affiliated medical institution.

⑫ The "Manager and Contact Person for Statistical Analysis" and the "staff who perform research other than the Principal Investigator/Co-investigator" are to submit the confirmation result (Form D) from their affiliated facility.

⑬ The "Manager and Contact Person for Statistical Analysis" and the "staff who perform research other than the Principal Investigator/Co-investigator" are to confirm the content of Form A and Form D, create Form E, and notify the Study Representative Physician/Study Secretariat

Description in the protocol and explanatory documents of conflicts of interest

⑭ The Study Representative Physician/Study Secretariat will confirm the contents of Form A and Form E received from each study center, and if necessary, any conflicts of interest with drug manufacturers and distributors, etc. in this study (study COI) are to be accurately described in the protocol and explanatory documents.

Review by the Certified Clinical Research Review Board

⑮ The Study Representative Physician/Study Secretariat are to compile and submit Form E and the “List of Co-investigators” from all study centers to the Certified Clinical Research Review Board for review.

2) Procedures when new involvements with companies, etc. occur after the start of research

⑯ When new involvements with companies, etc. (study COI) occur after the start of research

a. If there are changes in the companies, etc. involved in this study described in Q1 of Form B, repeat the procedure of steps [2] to [15]. If it is necessary to add new study COI to the protocol and explanatory documents, submit it for review to the Certified Clinical Research Review Board together with the changes in the protocol content (see 11.1.3.).

b. If changes occur from Q2 to Q5 in Form B, the Principal Investigators from the participating facilities affected by the changes are to change the description in the corresponding part of Form B, to update Form E, and to send the changes to the Study Representative Physician/Study Secretariat. The Study Representative Physician/Study Secretariat who received the sent Form E will submit it to the Certified Clinical Research Review Board, along with any changes to the content of the protocol (see 11.1.3.), as appropriate, for review.

⑰ When new involvements with companies, etc. (individual COI) with a conflict of interest declarer occur after the start of research

Conflict of interest declarers are to repeat the procedure from [7] to [9] or [11] to [13]. However, if there are no changes in Form E, these procedures will be performed at each facility, but forms will not be sent to the Study Representative Physician/Study Secretariat. The Study Representative Physician/Study Secretariat who received the modified Form E will submit it to the Certified Clinical Research Review Board for review.

3) Procedures for periodic reporting

The Study Representative Physician/Study Secretariat will confirm the study COI and the individual COI once per year at the regular reporting time and will report to the Certified Clinical Research Review Board whether there are any changes.

13.10.2. COI with companies, etc. related to this research

Some medicines are provided free of charge by companies in this study. In addition, this study has received funding from a number of companies for the transportation of drugs to the study centers. Although this study may include researchers who have conflicts of interest with companies, conflicts of interest are appropriately managed in accordance with the Clinical Research Act, the Clinical Research Act Enforcement Regulations, and related notices. For the names of specific medicines and pharmaceutical companies, see “19. Appendix: List of medicines.”

13.11. Compensation

As this study complies with the Clinical Research Act, insurance coverage must be obtained, a system to provide medical care must be secured, and other necessary measures must be taken to compensate for the health damage that occurs during the implementation of this study and to provide medical care. Therefore, medical insurance is to be provided to make available appropriate medical treatment according to the medical condition that is equivalent to usual medical care for health hazards caused by participating in this study. In addition, this study will obtain clinical research insurance and will compensate for the following based on the terms of insurance, and these points are to be explained to patients to gain their understanding.

① Medical treatment costs
Among the treatment expenses required for treatment of health damage, the amount paid will be the amount paid by the patient less the benefit from health insurance, etc.

② Medical allowance
Various expenses other than medical expenses are paid for health hazards that require hospitalization.

The causal relationship between the protocol treatment of this study and the health hazard is to be based on the judgment by the Study Representative Physician.

13.12. Intellectual property

The results, data, and intellectual property rights that are obtained from this study for each drug cohort, and the ownership thereof, are described in the separately concluded contract following consultation between the National Cancer Research Center and the pharmaceutical company that provided the corresponding drug without charge.

Researchers from the medical institutions participating in this study are to notify pharmaceutical companies in advance when presenting research results. Regarding the specific handling, each pharmaceutical company and each study center are to discuss and decide with the Study Representative Physician and Study Secretariat playing a central role.

13.13. Disclosure of information relating to this research

A summary, the progress, and the main results from this study will be published on jRCT (https://jrct.niph.go.jp/). Registration to jRCT is performed by the medical institution to which the Study Representative Physician belongs.

In addition, since this research is conducted based on the patient-proposed healthcare service system, it is described as a [Patient-proposed healthcare service] in the “Supplementary items throughout” field when registering in jRCT.

14. Monitoring and audits

14.1. Periodic monitoring

Periodic monitoring is conducted in this study to confirm that the study is being conducted safely and according to the protocol, and that data are being collected correctly. Periodic monitoring will be centralized monitoring based on the data input to eCRFs collected via EDC system, and will be performed by data management staff. In principle, it will be conducted twice per year.

The data management staff are to submit a "monitoring report" that summarizes the results of the centralized monitoring to the Study Representative Physician/Study Secretariat. Specific procedures for centralized monitoring will be determined separately in the procedure manual.

Monitoring reports serve as reference materials for periodic reporting according to the Clinical Research Act, periodic reporting on patient treatments, performance reports and reports to pharmaceutical companies providing drugs without charge (see 13.6.).

14.1.1. Items monitored

The following items are to be described in the monitoring report.

① Research summary
② Enrollment status: number of enrollments - cumulative, by period, by facility, by drug
③ Input status: by facility
④ Background factors: by drug
⑤ During protocol treatment/by treatment completion, discontinuation/termination reason: By drug
⑥ Protocol compliance: eligibility review, possible deviation, possible noncompliances and major noncompliances - by facility
⑦ Safety report information: by facility, by drug
⑧ Best overall response in patients with measurable lesions up to 16 weeks after starting treatment: only patients with measurable lesions

Other issues related to study progress and safety (occurrence of noncompliance and response thereto, number of cases covered by compensation, number of diseases, etc. reported under Article 13 of the Clinical Research Act)

14.1.2. Eligibility for enrollment (eligibility, ineligibility)

Eligibility is classified according to the following definition for all enrolled patients. For monitoring, an example where data management personnel might be ineligible will be listed in the “Protocol Compliance Status” field of the monitoring report. The Study Representative Physician/Study Secretariat will provisionally confirm one of 1), 2) and 99). Ultimately, after review by the Study Representative Physician/Study Secretariat and prior to conducting the main analysis, one of 1), 2) and 99) will be determined.

1) Eligibility only is considered as an “eligible case,” and 2) Ineligibility and 99) Enrollment violation are considered as “ineligible cases.” This is a classification set from the viewpoint of setting the analysis set.

1) Eligibility

According to the methods and criteria defined in the protocol, the information generated prior to enrollment meets all patient selection criteria.

2) Ineligibility

None of the patient selection criteria are met either before or after enrollment.

99) Enrollment violation

When a patient who does not meet the selection criteria is intentionally (falsely) enrolled This constitutes to a false report and is handled as a serious problem.

- When a patient is enrolled and the protocol treatment is administered without the required informed consent.
- When the source documents used to determine eligibility cannot be verified (including instances of loss of the consent form).

14.1.3. Protocol deviations/violations

Protocol deviations are when the treatment, such as drug administration, clinical examination, or evaluation of toxicity and efficacy are not performed according to the provisions of the protocol.

Deviations during monitoring that exceed a certain allowable range established in advance for each study or between the data management staff and the Study Representative Physician/Study Secretariat after the start of the research are listed in the monitoring report as a "probable deviation," are temporarily confirmed as one of the following by the Study Representative Physician/Study Secretariat are classified into any of the following. Deviations will ultimately be definitively confirmed as one of the following prior to conducting the main analysis and after review by the Study Representative Physician/Study Secretariat.

1） Violation

Clinically inappropriate deviations from the protocol provisions that fall under multiple protocol items below and are caused by the Principal Investigator, Co-investigators, or the facility are considered as "violations."

① Substantial impact on the assessment of research endpoints
② Deliberate or systematic
③ The degree of risk or deviation is significant

As a rule, "Violations" describes the content of individual violations when disclosed in a publication.

2） Deviation

A deviation does not correspond to the violation in 1). If there are many specific deviations, it is desirable to describe them at the time of disclosure in a publication. These are classified in the monitoring report as any of the following.

① Deviations
② Deviation (unavoidable) - (example: postponement due to the year-end and New Year holidays, equipment failure, etc.)
③ Deviation (clinically appropriate)

※ Deviation does not always mean that there is a problem with the institution's Principal Investigator or Co-investigators. Patient safety is the first priority in a clinical trial, and so it is better that the Principal Investigator or Co-investigator would "deviate" on the basis of judgment if it is judged that it is hazardous to follow the protocol in view of the individual patient's condition. If it is judged as a clinically relevant deviation for the safety of the patient, it is recorded as the above [3] "Deviation (clinically appropriate)." It is not considered to be a particular problem if there are few clinically appropriate deviations, but with multiple occurrences there is a high possibility that the protocol specification will be inappropriate and it is thus necessary to consider protocol revisions. However, deviations that are performed with an intention other than safety (such as shortening of the treatment period outside the protocol specification) are not considered to be "clinically appropriate deviations."

14.2. Audits

From the viewpoint of securing reliability in clinical research and protecting subjects of clinical research, in this study we will conduct facility visit audits to ensure the reliability of the materials and information collected through the clinical research.

In a facility visit audit, an auditor appointed by the Study Representative Physician visits the study center, confirms the approval documents for that medical institution, confirms the list of Co-investigators who conduct the research, confirms the explanatory and consent documents, and confirms the data input to the CRFs matches medical records (direct reading of original materials), etc. Specific procedures for facility visit audits are specified in a separate procedure document.

The auditors are to prepare an "audit report" that summarizes the audit results, and submit this to the Study Representative Physician/Study Secretariat, and Principal Investigator.

14.3. Managing noncompliance

14.3.1. Noncompliance

According to the Clinical Research Act, noncompliance means "a situation in which the clinical research

does not comply with the Clinical Research Act Enforcement Regulations or the research protocol.” Noncompliances are categorized into minor noncompliances and major noncompliances.

If it is found that the facility is noncompliant (regardless of whether or not it is a major noncompliance) prior to the implementation of central monitoring and auditing, the Principal Investigator will promptly report this to the Study Representative Physician/Study Secretariat.

14.3.2. Minor noncompliance

Examples of "minor noncompliances" are shown below. Minor noncompliances should be reported by the principal investigator to the director of the study center by submitting a biannual monitoring report, or excerpts or a summary thereof describing the noncompliances.

- Items pertaining to 2) Ineligibility in 14.1.2. Eligibility for enrollment (eligibility, ineligibility)
- Items pertaining to 2) Deviation in 14.1.3. Protocol deviations/violations

14.3.3. Major noncompliance

A major noncompliance is one that affects the human rights and safety of the subject in a clinical study and affects the reliability of the research progress and results. Examples of “major noncompliance” are shown below. If there is a possibility of major noncompliances, the Study Representative Physician/Study Secretariat will promptly report to the Certified Clinical Research Review Board as soon as the situation is known.

- Items related to 99) Enrollment violation in 14.1.2. Eligibility for enrollment (eligibility/ineligibility)

-
- Items relating to 1) Violation in 14.1.3. Protocol deviations/violations
- Conducting the study prior to approval by the Certified Clinical Research Review Board or before approval by the Study Center Director
- Continuing the study without providing information that could affect a patient's intention to continue in the study
- Activities judged to be research fraud (data forgery, data falsification, etc.)
- Personal information leaks or human rights violations that have a significant impact on enrolled patients

14.3.4. Noncompliance exclusion items

The following items do not constitute a “noncompliance” in this study.

- Changes to the protocol and implementation plan due to the transfer of a principal investigator require review by the Certified Clinical Research Review Board and notification of the implementation plan to the Minister of Health, Labor and Welfare, and completion of this series of procedures will take some time. Furthermore, it is often difficult to complete the change procedures before the transfer because the transfer may not be announced until just before the transfer is made. Therefore, even if the principal investigator is absent for a period of time due to a transfer, this will not constitute a “noncompliance” as long as the research management system is being maintained by the co-investigators and the medical treatment system for surviving patients is being secured.

- In instances of 2) Ineligibility in 14.1.2. Eligibility for enrollment (eligibility, ineligibility), “Ex post ineligibility” means failure to meet any of the patient inclusion criteria due to information having come to light after enrollment, or failure to meet any of the inclusion criteria due to information known before enrollment but through means or criteria other than those stipulated by the protocol. “Ex post ineligibility” does not constitute noncompliance with the protocol, and therefore will not be treated as “noncompliance” under the Clinical Research Act in this study.

Examples of ex-post ineligibility

(1) In the study for stage II-III patients, bone metastasis was found and stage IV was diagnosed upon performing bone scintigraphy immediately after enrollment. Protocol treatment was discontinued.

(2) In a study on early-stage gastric cancer, blood stool was observed after enrollment and colonoscopy was performed. Advanced colorectal cancer (synchronous multiple cancer) was found. The protocol was discontinued and colon resection was performed.

(3) In a study on gastric cancer (adenocarcinoma), pathological diagnosis at the facility was changed to malignant lymphoma after enrollment.

15. Special notes

15.1. Additional study centers

In this study, the National Cancer Center Hospital will assume responsibility for the Study Representative Physician and the overall coordination office, and the cancer genome-based healthcare core base hospitals will participate as multi-institutional joint research facilities recognized as collaborative medical institutions for the patient-proposed healthcare services.

15.1.1. Requirements for study centers

This study corresponds to the off-label use of medicines that have not been confirmed to be safe for administration to subjects in Japan, and for which pharmaceutical approval has not been obtained. Therefore, it is necessary to have a medical treatment system capable of responding promptly to adverse events, and to maintain a system for consulting an expert panel regarding known genetic abnormalities. Therefore, only medical institutions that meet all the following conditions can participate in this study.

1) Cancer genome-based healthcare core base hospitals.
2) Having a system in place to respond appropriately 24 hours a day, 365 days a year when a serious adverse event occurs
3) Having a system that can register information in C-CAT

Furthermore, if a patient who wishes to participate in this research visits a medical institution other than the medical institution where the research is conducted, it is recommended to explain the purpose of the patient-proposed healthcare service system and have the patient make a medical visit to one of the study centers.

*Pediatric patients (under 15 years old) may be enrolled if conditions 1) through 3) are met and the medical institution is a central organization for pediatric cancer or a base hospital for pediatric cancer.

15.2. Determination of whether or not to continue with patient-proposed healthcare service

When the Study Representative Physician/Study Secretariat receives information that affects the implementation of the patient-proposed healthcare service, such as when a company starts clinical trials due to changes in the development status of the drug in Japan after the start of this research, etc., the opinion of the Study Representative Physician/Study Secretariat concerning this status change and the corresponding policy is to be promptly reported in writing or via email to the Efficacy and Safety Evaluation Committee with a request for the opinion of the Committee on whether or not it is possible to continue the study or if the situation requires a change of the eligibility criteria, etc. In addition, these results will be reported to the Patient-proposed Healthcare Service Evaluation Committee to request a determination as to whether or not to continue the patient-proposed healthcare service.

16. Research organization

16.1. Source of funding for this research

According to the patient-proposed healthcare service system, of the medical expenses paid to the study center by the enrolled patients, the operating expenses to be remitted from the study center to the National Cancer Research Center are based on the contract.

- Ministry of Health, Labor, and Welfare's Health Labor Sciences Research Grant (Comprehensive Research Project for the Promotion of Cancer Control)
- Japan Agency for Medical Research and Development (AMED) Research Grant (Research Project for Practical Application of Innovative Cancer Treatments)
- Research grant offered by Ono Pharmaceutical Co., Ltd. (costs of drug transport)
- Research grant offered by Otsuka Pharmaceutical Co., Ltd (cost of drug transport)

16.2. Study Representative Physician

Noboru YAMAMOTO
Department of Experimental Therapeutics, National Cancer Center Hospital
5-1-1, Tsukiji, Chuo-ku, Tokyo, JAPAN 104-0045
Tel: 03-3542-2511 (direct line 7319)
Fax: 03-3542-3567
E-mail: nbryamam@ncc.go.jp

16.3. Study secretariat

Tatsunori SHIMOI
Department of Medical Oncology, National Cancer Center Hospital
5-1-1, Tsukiji, Chuo-ku, Tokyo, JAPAN 104-0045
Tel: 03-3542-2511 (direct line 2331)
Fax: 03-3542-3567
E-mail: tshimoi@ncc.go.jp

Kuniko SUNAMI
Department of Pathology and Clinical Laboratories, National Cancer Center Hospital
5-1-1, Tsukiji, Chuo-ku, Tokyo, JAPAN 104-0045
Tel: 03-3542-2511 (direct line 7751)
Fax: 03-3542-3567
E-mail: ksunami@ncc.go.jp

16.4. Participating facilities (study centers)

Facilities participating in this study are:

Study Center Name	Name of the department	Principal Investigator	Enrollment of pediatric patients*
National Cancer Center Hospital	Department of Experimental Therapeutics	Noboru YAMAMOTO	○
Hokkaido University Hospital	Department of Medical Oncology	Ichiro KINOSHITA	○
Tohoku University Hospital	Department of Medical Oncology	Chikashi ISHIOKA	○

National Cancer Center Hospital East	Department of Medical Oncology	Tooru MUKOUHARA	-
Keio University Hospital	Cancer Center	Hiroshi NISHIHARA	-
The University of Tokyo Hospital	Department of Genomic Medicine	Katsutoshi ODA	-
Shizuoka Cancer Center	Respiratory Internal Medicine	Hirotomo KENMOCHI	-
Nagoya University Hospital	Department of Advanced Medicine	Satoshi NISHIWAKI	○
Kyoto University Hospital	Department of Clinical Oncology	Manabu MUTO	○
Osaka University Hospital	Center for Cancer Genomics and Personalized Medicine	Naohiro Nishida	-
Okayama University Hospital	Clinical Cancer Center	Masahiro TABATA	-
Kyushu University Hospital	ARO Next Generation Medical Center	Eishi BABA	○

- Pediatric patients (under 15 years old) may be enrolled only in facilities marked with ○.

16.5. Matters concerning persons engaged in clinical research other than the Principal Investigator

16.5.1. Data Management Supervisor

Director
Data Management Office, Data Management Section, Clinical Research Support Office, National Cancer Center Hospital
Data Management Office Chief　　Yukari HOSHINA

16.5.2. Monitoring Specialist

Data Management Section, Clinical Research Support Office, National Cancer Center Hospital
Data Management Section Head　　Haruhiko FUKUDA

16.5.3. Audit Director

Auditing Section, National Cancer Center
Hidekazu ARAI

16.5.4. Statistical Analysis Responsible Person

Biostatistics Division, Center for Research Administration and Support, National Cancer Center
Section Head　　Taro SHIBATA

16.5.5. Research and Development Planning Support Supervisor

Department of Medical Oncology, National Cancer Center Hospital
Tatsunori SHIMOI

Department of Pathology and Clinical Laboratories, National Cancer Center Hospital
Kuniko SUNAMI

16.5.6. Coordination/Management Responsible Party

Clinical Research Support Office, National Cancer Center Hospital
Yayoi ANDO, Kanako KONDO, Sachie KAWABATA, Takako HITOMI, Natsuko

OKITA, Ken-ichi NAKAMURA (manager)

16.5.7. Efficacy and Safety Evaluation Committee

Clinical Investigation and Research Unit, Aichi Cancer Center Research Institute	Masashi ANDO
Department of Respiratory Oncology, Kansai Medical University Hospital	Takayasu KURATA
Department of Hematology, NTT Medical Center Tokyo	Kensuke USUKI

16.5.8. Privacy Protection Management Director

Data Management Section, Clinical Research Support Office, National Cancer Center Hospital

Data Management Section Head　　Haruhiko FUKUDA

16.5.9. Privacy Protection Supervisor

Data Management Office, Data Management Section, Clinical Research Support Office, National Cancer Center Hospital

Data Management Office Chief　　Yukari HOSHINA

17. Development of research results and completion of the study

17.1. Publications and presentations at professional conferences

The main publications will be published in English.

Presentations at academic conferences and publications (review articles) for the purpose of introducing research that does not include the analysis results from the study endpoints, and the presentation on the distribution of patient background and safety data at academic conferences and publications after enrollment has been completed can be done with permission from the Study Representative Physician.

When the results of the primary analysis of the drug cohort are reported in a paper as the first report through the study (the first publication to disclose results for the primary endpoint), the Study Secretariat will serve as the lead author. As a general rule, the lead author of the main publication disclosing the research results for each drug cohort (the first publication to disclose the primary endpoint results) other than the above-mentioned first report will be the principal investigator or co-investigator of the study center with the largest number of enrolled patients, and the Study Secretariat and the Statistical Analyst will be secondary authors. Below that, in accordance with any restrictions imposed by the manuscript submission regulations, the coauthors will be named in descending order of the number of enrollments, and the final author and corresponding author will be the Study Representative Physician.

All coauthors should review the content of the manuscript before submission, and only be those who have agreed to the content of the publication.

The lead presenter of main conference presentations for each drug cohort (the first conference presentation of the primary endpoint results) should in principle be the principal investigator or co-investigator of the study center with the second largest number of patients enrolled. Other conference presentations can take place more than once, so the presenters will be drawn from among the Study Secretariat, the Study Representative Physician, and the Principal Investigator or the Co-investigators of the study center with the most enrollments. The presenter will be decided by the Study Representative Physician in consultation with the Principal Investigator at the study center.

Note that, because this study involves a large number of drugs, there may be cases in which it is difficult to make decisions based on the above provisions; in such cases, the Study Representative Physician will make the decision with the above regulations and policies as a guide.

In addition, when making external publications related to this study, regardless of whether it is in the form of a paper or a presentation at an academic conference, the necessary exchange of data, analysis results and information must take place through communication from the principal investigator of the medical institution responsible for the presentation to the Study Secretariat, and no data, analysis results or information may be exchanged with other concerned parties without going through the Study Secretariat. The contents of external publications related to this study must be confirmed by the Study Secretariat, Statistical Analyst, and principal investigator prior to the review by all co-authors. The contents of external publications related to this study must be confirmed by the Study Secretariat, the Statistical Analyst, and the Study Representative before review by all co-authors.

In addition, the Study Secretariat will notify the pharmaceutical company that provided the relevant medication(s) concerning the presentation in accordance with the terms of the contract concluded between the National Cancer Center and each pharmaceutical company.

17.2. Primary endpoint report/Final Report

Within 1 year from the date of the final analysis report, the Study Representative Physician is to prepare the primary endpoint report and the Final Report and its summary for this study in its entirety, and along with the final analysis report, submit these to the Certified Clinical Research Review Board and the Hospital Director of the National Cancer Center Hospital.

The Study Representative Physician/Study Secretariat are to register the summary of the Final Report (Rule Article 24 Attached Form 1, Completion Notification Form) with jRCT within one month after approval by the Certified Clinical Research Review Board is obtained. (The summary need not be published in jRCT if the manuscript has not been published, but this should be done promptly after publication of the manuscript). Together with the publication in jRCT, a summary of the report is to be

submitted to the Minister of Health, Labour and Welfare, accompanied by the protocol and the informed consent documents. The approved summary report will be submitted to the Study Center Directors of each study center via the Principal Investigator at each facility.

The date on which the summary report is registered in jRCT is taken as the study completion date.

17.3. Completing the study

After receiving the final analysis report, the Study Representative Physician/Study Secretariat will report the summary of the results to the research staff at the participating facilities together with the report that the study has been completed. The timing of distribution of the final analysis reports to the participating facilities is determined by the Study Representative Physician/Study Secretariat in consideration of the timing of publication of the main results, etc., and these are to be distributed to the research staff at the participating facilities.

The Principal Investigator who received the report on the completion of the study will report the completion of the study and the summary of the results in writing to the Study Center Director without delay. The summary of the results can be reported using the “Final Report” prepared by the Study Representative Physician/Study Secretariat after the final analysis is completed.

Furthermore, for facilities in which no patient enrollment was carried out, the date of enrollment completion can also be taken as the date of study completion for those corresponding facilities.

18. Literature citations

1. Rare Cancer Medical Services/Support Study Group. 2015 Rare Cancer Medical Services/Support Study Group Report.
2. Greenlee RT, Goodman MT, Lynch CF, Platz CE, Havener LA, Howe HL. The occurrence of rare cancers in U.S. adults, 1995-2004. Public health reports (Washington, DC : 1974). 2010;125(1):28-43.
3. Tamaki T, Dong Y, Ohno Y, Sobue T, Nishimoto H, Shibata A. The burden of rare cancer in Japan: application of the RARECARE definition. Cancer epidemiology. 2014;38(5):490-5.
4. Boyd N, Dancey JE, Gilks CB, Huntsman DG. Rare cancers: a sea of opportunity. The Lancet Oncology. 2016;17(2):e52-e61.
5. List of cancer genome-based healthcare core base hospitals and cancer genome-based healthcare cooperating hospitals (as of October, 2018).
6. Clinical practice guidance for cancer diagnosis and treatment based on gene panel tests using a next-generation sequencer, etc. (Version 1.0).
7. Le DT, Uram JN, Wang H, Bartlett BR, Kemberling H, Eyring AD, et al. PD-1 Blockade in Tumors with Mismatch-Repair Deficiency. The New England journal of medicine. 2015;372(26):2509-20.
8. Cocco E, Scaltriti M, Drilon A. NTRK fusion-positive cancers and TRK inhibitor therapy. Nature reviews Clinical oncology. 2018;15(12):731-47.
9. Zehir A, Benayed R, Shah RH, Syed A, Middha S, Kim HR, et al. Mutational landscape of metastatic cancer revealed from prospective clinical sequencing of 10,000 patients. Nature medicine. 2017;23(6):703-13.
10. Clinical practice guidance for cancer diagnosis and treatment based on gene panel tests using a next-generation sequencer, etc. (ver. 2.1)
11. Mody R, Wu Y, Lonigro R et al. Integrative Clinical Sequencing in the Management of Refractory or Relapsed Cancer in Youth. JAMA. 2015;314(9):913-25.
12. Chuk M, Mulugeta Y, Roth-Cline M et al. Enrolling Adolescents in Disease/Target-Appropriate Adult Oncology Clinical Trials of Investigational Agents. Clin Cancer Res. 2017;23(1):9-12.
13. Japanese translation JCOG version of the NCI Common Terminology Criteria for Adverse Events v 5.0 (CTCAE v5.0)".
14. Johnson KR, Mascall GC, Howarth AT. Differential laboratory diagnosis of hypercalcemia. Critical

reviews in clinical laboratory sciences. 1984;21(1):51-97.
15. Eisenhauer EA, Therasse P, Bogaerts J et al. New response evaluation criteria in solid tumours: Revised RECIST guideline (version 1.1). Eur J Cancer 2009; 45: 228-247.

19. Appendices

- Explanatory Document/Informed Consent Form
- Medicinal product list
- Pediatric Clinical Reference Ranges
- CTCAE Grade Definition Table for Pediatric Clinical Reference Ranges
- Performance Status Scales/Scores
