# Supplementary material for: Dabrafenib and trametinib administration in patients with BRAF V600E/R or non-V600 BRAF mutated advanced solid tumours (BELIEVE, NCCH1901): a multicentre, open-label, and single-arm phase II trial
Source: eClinicalMedicine. 2024 Feb 2;69:102447. doi: 10.1016/j.eclinm.2024.102447 (PMC10850114; doi:10.1016/j.eclinm.2024.102447)
Supplement: Supplementary Tables S1 — and S2 [file mmc2.docx]

**Supplementary Table 1. The cause of discontinuation due to adverse events (n = 3).**

| **Adverse events** | **Grade** |
| --- | --- |
| Pneumothorax | Grade 3 |
| Erythema nodosum | Grade 2 |
| Autoimmune disorder (adult Still's disease) | Grade 3 |

**Supplementary Table 2. Dose reduction and the corresponding cause**

| **Dose reduction** | **Number of cases** |
| --- | --- |
| Number of patients | 10 / 57 (17.5%) |
| Dose reduction frequency | 30 times |
| Cause of dose reduction |  |
| Skin rash | 5 |
| Fever | 3 |
| Increased aspartate aminotransferase | 2 |
| Increased alanine transaminase | 2 |
| Elevated creatine kinase | 2 |
| Decreased neutrophil count | 2 |
