# Supplementary material for: Dabrafenib and trametinib administration in patients with BRAF V600E/R or non-V600 BRAF mutated advanced solid tumours (BELIEVE, NCCH1901): a multicentre, open-label, and single-arm phase II trial
Source: eClinicalMedicine. 2024 Feb 2;69:102447. doi: 10.1016/j.eclinm.2024.102447 (PMC10850114; doi:10.1016/j.eclinm.2024.102447)
Supplement: Translational abstract [file mmc3.docx]

The following translations in Japanese were submitted by the authors and we reproduce them as supplied. They have not been peer reviewed. Our editorial processes have only been applied to the original abstract in English, which should serve as reference for this manuscript.

**翻訳抄録**

背景 *BRAF* V600遺伝子変異は、黒色腫、甲状腺癌、非小細胞肺癌によく認められる。ダブラフェニブとトラメチニブは特定のがんに対する標準治療薬であるにもかかわらず、様々な固形癌における有効性は未だ十分には検証されていない。BELIEVE試験では、*BRAF* V600E/Rまたは非V600の*BRAF*変異を有する固形がんにおけるダブラフェニブとトラメチニブの有効性を評価した。

方法 大腸がん、メラノーマ、非小細胞肺がん以外の*BRAF* V600E変異固形がん症例を対象とした。対象患者には、病勢進行または忍容できない毒性が認められるまで、ダブラフェニブ（150mg）を1日2回、トラメチニブ（2mg）を1日1回投与した。主要評価項目は客観的奏効割合（ORR）、副次的評価項目は無増悪生存期間（PFS）、6ヵ月PFS、全生存期間（OS）であった。ベイズ解析は、30％の奏効割合が期待される事前分布[Beta (0.6, 1.4)]を用いて行われた。

所見： 主に*BRAF* V600E変異（94％の症例）を有する測定可能病変を有する患者47人と、V600E以外の*BRAF*変異（V600R、G466A、N486_P490del）を有する患者3人が主要評価項目の対象症例として登録された。主な原発部位は甲状腺、中枢神経系、肝臓、胆管、大腸、膵臓であった。確認されたORRは28.0％であった。事後分布の期待値［Beta(14,6, 37.4)］は28.1％で、主要評価項目は達成したものの、ベイズ解析で得られた60％という予想外に高い奏効率には至らなかった。病勢コントロール率（DCR）は84.0％であった。PFS中央値は6.5ヵ月（95％信頼区間[CI]；4.2～7.2ヵ月、6ヵ月時87.8％）であった。奏効は7つのがん種で観察された。OS中央値は9.7ヵ月（95％信頼区間[CI]、7.5-12.2ヵ月）であった。測定可能な病変のない追加患者のPFS中央値は4.5ヵ月であった。有害事象（AE）は過去の報告と一致しており、45.6％の患者がグレード3以上のAEを経験した。

解釈 本試験では、*BRAF* V600変異腫瘍に対する有望な有効性が報告された。ダブラフェニブとトラメチニブは、高悪性度グリオーマ、胆道がん、甲状腺がんなどの希少がんに対する新たな治療選択肢を提供するであろう。

資金提供 本研究は、日本医療研究開発機構（20ck0106622h0001）および厚生労働科学研究費補助金（19EA1008）の助成を受けた。

キーワード ベイズ統計、プラットフォーム臨床試験、BRAF阻害剤、MEK阻害剤、希少がん
