# Supplementary material for: Dabrafenib and trametinib administration in patients with BRAF V600E/R or non-V600 BRAF mutated advanced solid tumours (BELIEVE, NCCH1901): a multicentre, open-label, and single-arm phase II trial
Source: eClinicalMedicine. 2024 Feb 2;69:102447. doi: 10.1016/j.eclinm.2024.102447 (PMC10850114; doi:10.1016/j.eclinm.2024.102447)
Supplement: Supplementary Figure S1 and S2 [file mmc4.pptx]

## Slide 1
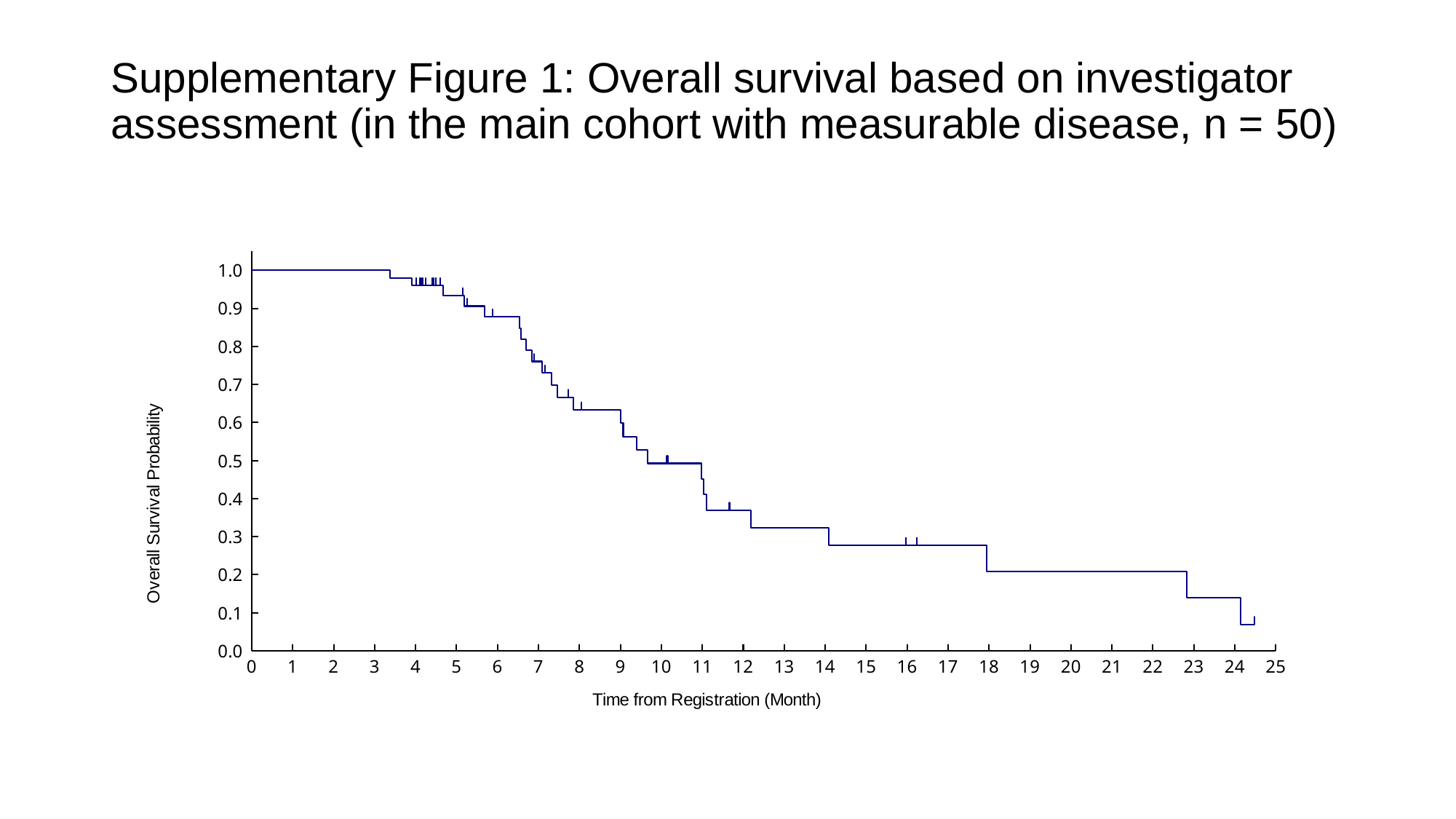

# Supplementary Figure 1: Overall survival based on investigator assessment (in the main cohort with measurable disease, n = 50)
### Chart
| Category | | |
|---|---|---|

## Slide 2
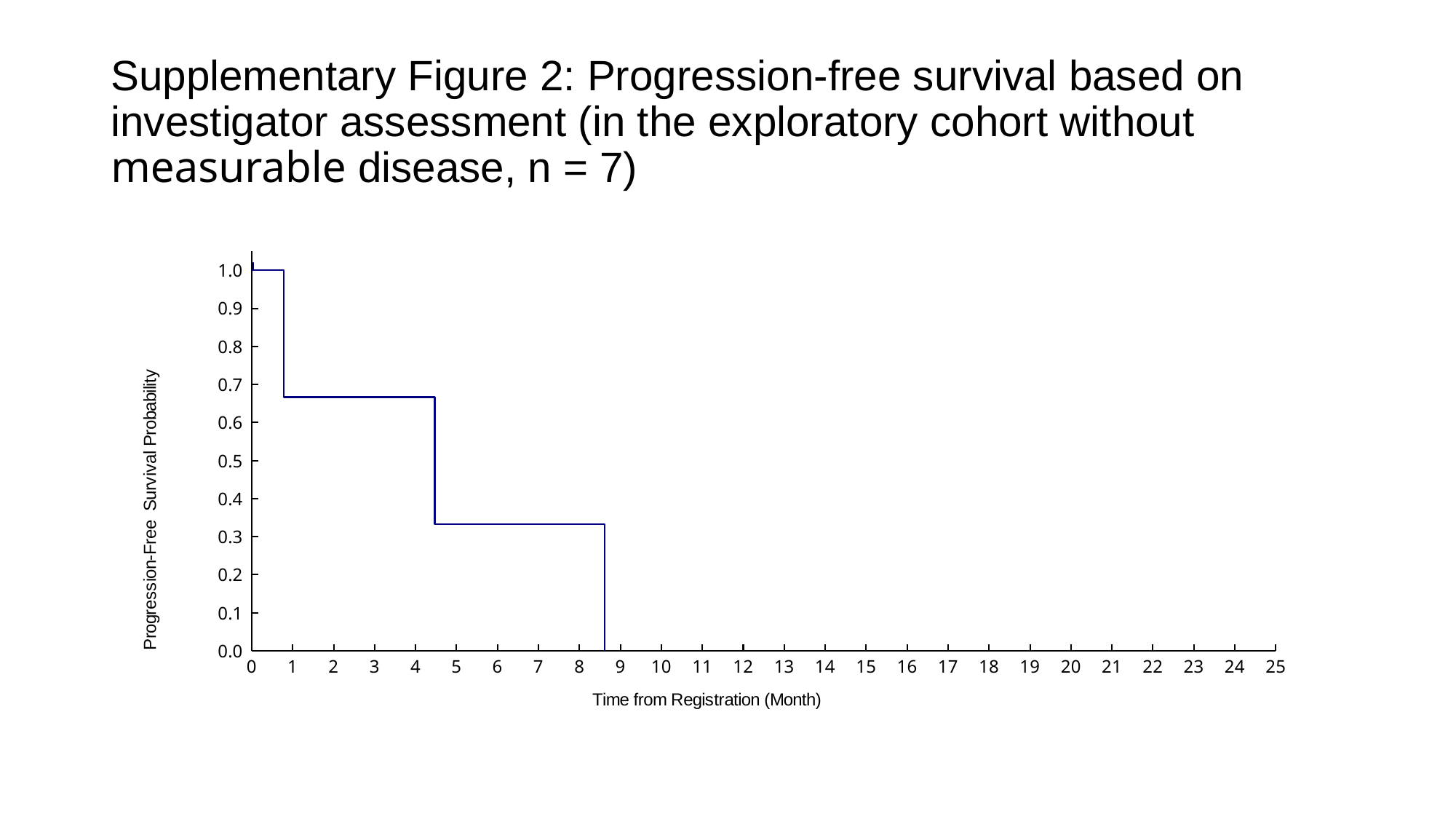

# Supplementary Figure 2: Progression-free survival based on investigator assessment (in the exploratory cohort without measurable disease, n = 7)
### Chart
| Category | | |
|---|---|---|
